# Orbital Hall conductivity and relaxation in thin films with variable disorder

**DOI:** 10.1038/s41567-026-03334-z

**Published:** 2026-06-15

**Authors:** Min-Gu Kang, Federica Nasr, Giacomo Sala, Richard Schlitz, Shilei Ding, Stefan Bartsch, Marta D. Rossell, Santos F. Alvarado, Pietro Gambardella

**Affiliations:** 1 Department of Materials, ETH Zurich, Zurich, Switzerland; 2 Department of Quantum Matter Physics, University of Geneva, Geneva, Switzerland; 3 Department of Physics, University of Konstanz, Konstanz, Germany; 4 Electron Microscopy Center, Empa – Swiss Federal Laboratories for Materials Science and Technology, Dubendorf, Switzerland

**Keywords:** Spintronics, Magnetic properties and materials

## Abstract

Electric-field-induced orbital angular momentum has emerged as a fundamental electronic degree of freedom in solid-state devices. It complements the spintronic functionalities used to sense and manipulate magnetic states. However, how orbital angular momentum evolves and relaxes in conductors with various degrees of structural and thermal order remain poorly understood. Here we demonstrate that the orbital Hall effect and orbital relaxation are robust and quantifiable in strongly disordered Mn thin films up to single-crystalline α-Mn. Using orbital Hanle magnetoresistance as a probe, we find that the orbital Hall conductivity scales linearly with electrical conductivity in the hopping-dominated regime from amorphous to polycrystalline films. This reveals a scaling behaviour analogous to the anomalous and spin Hall effects in bad metals. The orbital relaxation time, of the order of picoseconds, decreases with increasing crystalline order. Temperature-dependent measurements identify distinct relaxation channels. We observe in disordered films a crossover from on-site orbital dephasing to interorbital hopping with increasing temperature. In single-crystalline α-Mn, orbital relaxation is dominated by Elliott–Yafet-type scattering. Our findings establish orbital angular momentum as a structurally resilient degree of freedom and elucidate its relaxation dynamics across crystalline and disordered systems, thus enabling orbital control in different classes of materials.

## Abstract (web summary)

The role of disorder in orbital angular momentum transport induced by electric fields is not well understood. Now orbital Hall conductivity and orbital relaxation are shown to be robust even in strongly disordered thin films.

## Main

The coupling of charge, spin and orbital degrees of freedom underpins fundamental electron transport processes in condensed matter, with practical implications for realizing efficient spintronic and orbitronic devices. Whereas spin transport is well established and widely exploited in magnetic sensing and data storage applications^1–3^, the generation and control of orbital angular momentum (OAM) currents and their role in mediating electronic and magnetic interactions have only recently gained attention. Theoretically, orbital currents can emerge in crystalline materials under an applied electric field, even in the absence of spin–orbit coupling or inversion symmetry breaking^4–7^. This effect arises from the momentum-dependent superposition of Bloch wavefunctions with different orbital characters, known as orbital texture, which enables an orbital Hall effect as a transverse response to an applied electric field, analogous to the spin Hall effect^5,7^. Recent experiments have demonstrated electric-field-driven OAM generation in non-magnetic light metals, which results in current-induced orbital accumulation^8,9^, orbital torque^10–16^ and different types of orbital magnetoresistance^17–21^. Reciprocal phenomena, such as the pumping and dissipation of OAM from a magnetic layer into a non-magnetic metal^22–24^, further establish the orbital degree of freedom as an essential ingredient in the description of magnetotransport, alongside spin and charge.

Despite these developments, fundamental questions remain regarding the generation, propagation and relaxation of OAM in materials with various degrees of crystalline and thermal disorder. Our current understanding of the orbital Hall effect is based on ideal crystalline systems^4–7^, in which the effects of the intrinsic band structure dominate. Theoretical models including disorder as a weak perturbation predict that the intrinsic orbital Hall conductivity ${\sigma }_{{\rm{L}}}$ is robust against structural and thermal disorder in ‘good metals’ with intermediate conductivity ${10}^{4}\le \sigma \le {10}^{6}\,{\Omega }^{-1}\,{\rm{c}}{{\rm{m}}}^{-1}$ (refs. ^25–29^), whereas impurity scattering may lead to a large extrinsic orbital Hall effect in ultraclean systems^30,31^. By contrast, the regime of strong disorder, where the conductivity is thermally activated, remains unexplored, both theoretically and experimentally.

The question of OAM transport is also debated. Whereas tight-binding calculations predict long-range orbital propagation along specific momentum directions coinciding with band degeneracy ‘hotspots’^15,32^, considerations based on crystal-field dephasing severely restrict coherent orbital transport in ordered crystals and polycrystals^25,27,33^. Experiments relying on orbital torque measurements to extract OAM parameters find orbital diffusion lengths that depend on the chemical order in magnetic alloys, in agreement with the hotspot theory^15^, but also widely differing orbital diffusion lengths exceeding 10 nm in polycrystalline light metals^8,9,12,14^. These large orbital diffusion lengths imply long orbital relaxation times, which are at odds with orbital quenching arguments.

Understanding orbital relaxation is, thus, crucial to elucidating OAM transport and accumulation phenomena. By analogy with spin relaxation^1^, orbital relaxation in crystalline systems can occur via Elliott–Yafet (EY)-like orbital-flip scattering or Dyakonov–Perel (DP)-like dephasing due to orbital precession induced by crystal fields^34^. As electron scattering events involve large momentum transfer and the mixing of orbital wavefunctions, EY relaxation is expected to dominate in centrosymmetric systems, leading to orbital relaxation times ${\tau }_{{\rm{L}}}$ proportional to the momentum scattering time *τ* and, hence, to the conductivity *σ* (ref. ^25^). However, recent theoretical work predicts a crossover to DP-like relaxation at sufficiently long *τ*, even in centrosymmetric systems, due to OAM precession induced by the orbital texture, resulting in ${\tau }_{{\rm{L}}}\propto {\tau }^{-1}$ (ref. ^34^). Experimental indications of such DP-like orbital relaxation have been obtained by spin–orbit torque measurements of ${\sigma }_{{\rm{L}}}$ in magnetic/non-magnetic multilayer heterostructures^35^. Yet torque measurements are typically restricted to a narrow range of rather high conductivity, complicated by shunting effects through different metal layers, and require the disentangling of spin and orbital effects in intervening metal spacers and magnetic layers with large spin–orbit coupling.

In this work we present a different approach to studying orbital generation and relaxation in a single-element metal layer with vastly different degrees of crystalline order and conductivity that span the so-called bad-metal to good-metal regime (*σ* ≈ 10^2^–10^4^ Ω^−1^ cm^−1^). We focus on Mn thin films using the orbital Hanle magnetoresistance (HMR) as a direct probe of orbital relaxation^17,21,36^, which is analogous to spin relaxation^37,38^. Mn provides a unique elemental platform for investigating orbital physics. In addition to its weak spin–orbit coupling, its half-filled 3*d* bands favour an orbital-singlet ground state^39^ that further suppresses spin–orbit-driven charge–spin conversion processes such as the spin Hall effect and isolates the orbital Hall effect as the dominant charge-to-angular-momentum conversion mechanism. Moreover, the ability of Mn to stabilize a variety of ordered and disordered structural phases^39^ makes it particularly versatile for tuning orbital responses.

## Mn thin films with tunable structural order

To systematically investigate how crystalline order governs orbital current generation and relaxation, we prepared a series of 10-nm-thick Mn films with various degrees of structural order, ranging from single-crystalline epitaxial layers to textured, polycrystalline and nanocrystalline-amorphous films. Single-crystal, textured and polycrystalline films were grown by molecular beam epitaxy (MBE). Crystalline quality was controlled via the choice of substrate and growth temperature, which enabled access to textured and polycrystalline phases with intermediate order (Methods). Nanocrystalline-amorphous films were grown by magnetron sputtering. We changed the Ar sputtering pressure to vary the degree of disorder. A higher pressure resulted in higher resistivity and enhanced structural inhomogeneity characterized by the coexistence of nanocrystalline and amorphous phases and increased surface roughness (Supplementary Note 1). This structural tuning was the primary control parameter in our study.

**Fig. 1:**
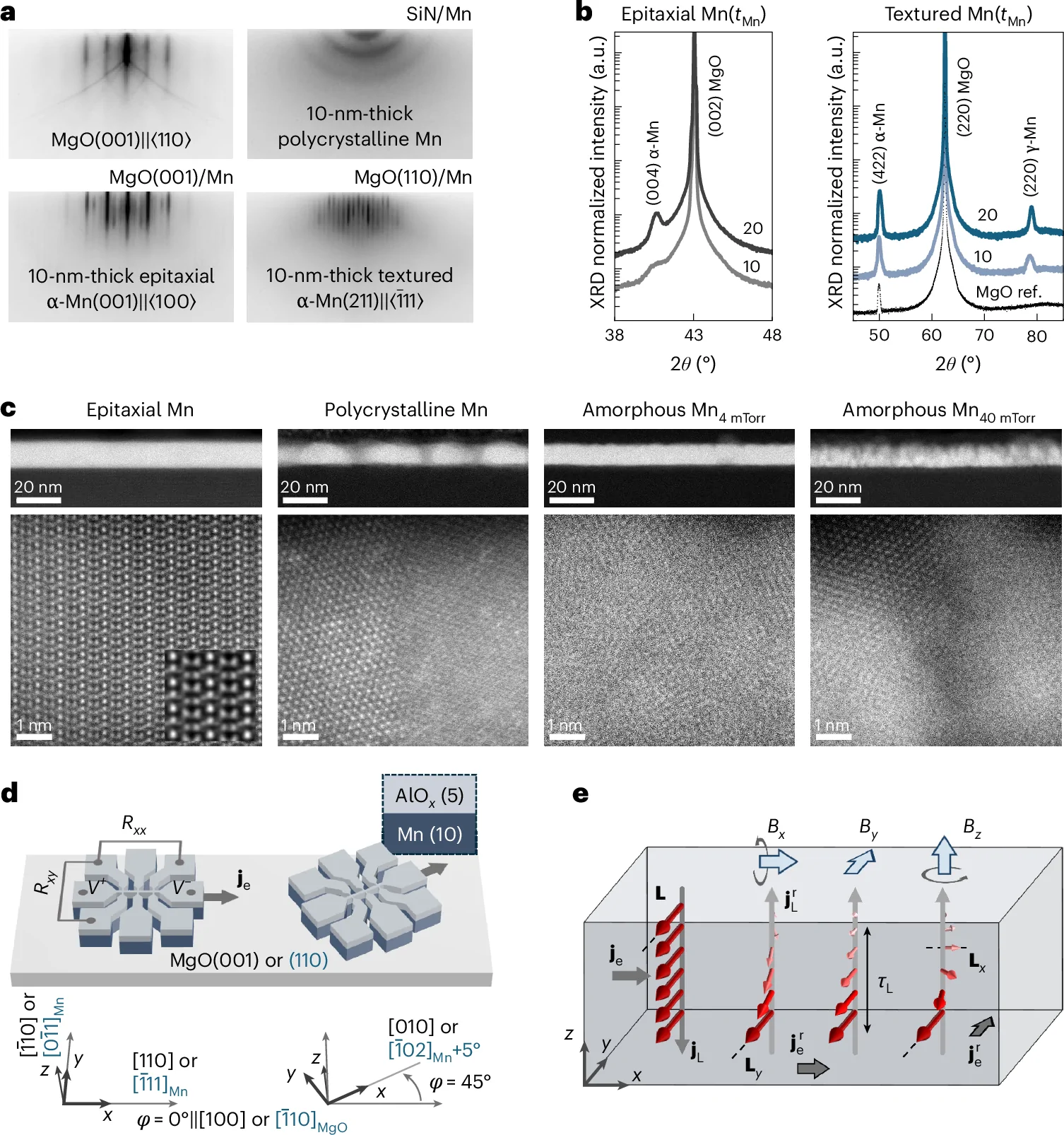
Structural characterization and device geometry of Mn thin films with various degrees of crystallinity. **a**, RHEED patterns of the MgO(001) substrate and of 10-nm-thick epitaxial α-Mn(001) on MgO(001), polycrystalline Mn on Si_3_N_4_, and textured Mn(211) on MgO(110). The data were acquired along the 〈110〉 and 〈$\bar{1}$10〉 azimuth of MgO(001) and (110), respectively, with an electron beam energy of 30 keV. **b**, XRD *θ/*2*θ* scans of MgO(001)/Mn(10, 20 nm) and MgO(110)/Mn(10, 20 nm) films, compared with the bare MgO(110) substrate. **c**, Cross-sectional microstructure of Mn thin films. Top: low-magnification HAADF-STEM images showing film thicknesses and surface morphology. Bottom: atomically resolved HAADF-STEM images. The epitaxial film, viewed along the [011] α-Mn zone axis, confirms an out-of-plane [001] film orientation. Inset: details of the atomic structure. **d**, Schematics of two Hall bar devices providing different current injection directions with superimposed electrical connections and coordinate systems. **e**, Schematic representation of the orbital Hanle effect. Source data

The degree of crystallinity was characterized in situ using reflection high-energy electron diffraction (RHEED) and ex situ via X-ray diffraction (XRD) and transmission electron microscopy. As shown in Fig. 1a, the sharp streaks in the RHEED patterns of epitaxial and textured Mn indicate smoothness and high crystalline order, whereas the ring patterns of polycrystalline Mn are indicative of random crystallite orientations. XRD measurements (Fig. 1b) identified the α-Mn phase with distinct (004) and (422) reflections from the epitaxial and textured films, respectively, in agreement with the RHEED analysis (Supplementary Note 1). Cross-sectional high-angle annular dark-field scanning transmission electron microscopy (HAADF-STEM) imaging confirmed the structural hierarchy across the Mn films (Fig. 1c). The epitaxial film had a high degree of crystalline order, with uniform thickness and atomically resolved lattice fringes characteristic of α-Mn viewed along the [011] zone axis. The polycrystalline film consisted of grains with lateral dimensions larger than the film thickness and separated by well-defined grain boundaries. By contrast, the sputtered films lacked long-range atomic order, displaying only nanoscale structural correlations within an otherwise disordered matrix. These films are referred to as amorphous in the following, although their fine structure depends on the sputtering pressure. Films deposited at 4 mTorr contained a relatively narrow distribution of nanocrystallites centred at ~1 nm, whereas films grown at 40 mTorr showed a broader (~1–10 nm) size distribution and increased roughness. These structural trends are fully consistent with the disorder evolution inferred from thickness- and temperature-dependent resistivity measurements (Supplementary Note 1).

## HMR in amorphous to crystalline Mn

To measure the HMR, samples were patterned as Hall bars oriented along different in-plane crystallographic axes for the epitaxial and textured Mn, as illustrated in Fig. 1d. Here the *x* axis is defined by the current injection direction, and *φ* denotes the angle between the current and the [110] and $[\bar{1}11]$ in-plane directions of epitaxial and textured Mn, respectively. We probed the longitudinal magnetoresistance ${R}_{xx}$ and transverse magnetoresistance ${R}_{xy}$ of the Mn films as a function of temperature while sweeping the magnetic field along three main axes (*x*, *y* and *z*). In paramagnetic Mn films with a thickness of 10 nm, the orbital HMR dominated the magnetoresistive response, outweighing contributions from the Lorentz magnetoresistance and ordinary Hall effect^17^. Figure 1e provides a conceptual schematic of the orbital HMR. The injection of an electric current ${{\bf{j}}}_{{\rm{e}}}$ into the Mn layer generates an orbital current (${{\bf{j}}}_{{\rm{L}}}$) along the *z* direction via the orbital Hall effect, which is partially reflected at the Mn boundaries (${{\bf{j}}}_{{\rm{L}}}^{{\rm{r}}}$). The non-equilibrium OAM ${\bf{L}}$ carried by the ${{\bf{j}}}_{{\rm{L}}}^{{\rm{r}}}$ is reconverted into a charge current (${{\bf{j}}}_{{\rm{e}}}^{{\rm{r}}}$) by the inverse orbital Hall effect, effectively lowering *R*_*xx*_. The non-equilibrium OAM is unaffected by an external magnetic field **B** ∥ **L** and reduced by a field ${\bf{B}}\perp {\bf{L}}$, which induces orbital precession and dephasing. The degree of resistance modulation thus depends on the alignment between the magnetic field and OAM, with maximum resistance observed for **B** ⊥ **L** and no resistance change for **B** ∥ **L**. This effect is like the spin HMR^37^. The strength of the orbital Hanle effect is determined by the orbital Hall conductivity ${\sigma }_{{\rm{L}}}$ and orbital relaxation time ${\tau }_{{\rm{L}}}$, in addition to the applied magnetic field. Thus, the orbital HMR provides insights into the generation and relaxation of OAM intrinsic to non-magnetic materials, without accounting for intermediate orbital–spin conversion steps as required in non-magnetic/magnetic bilayers.

**Fig. 2:**
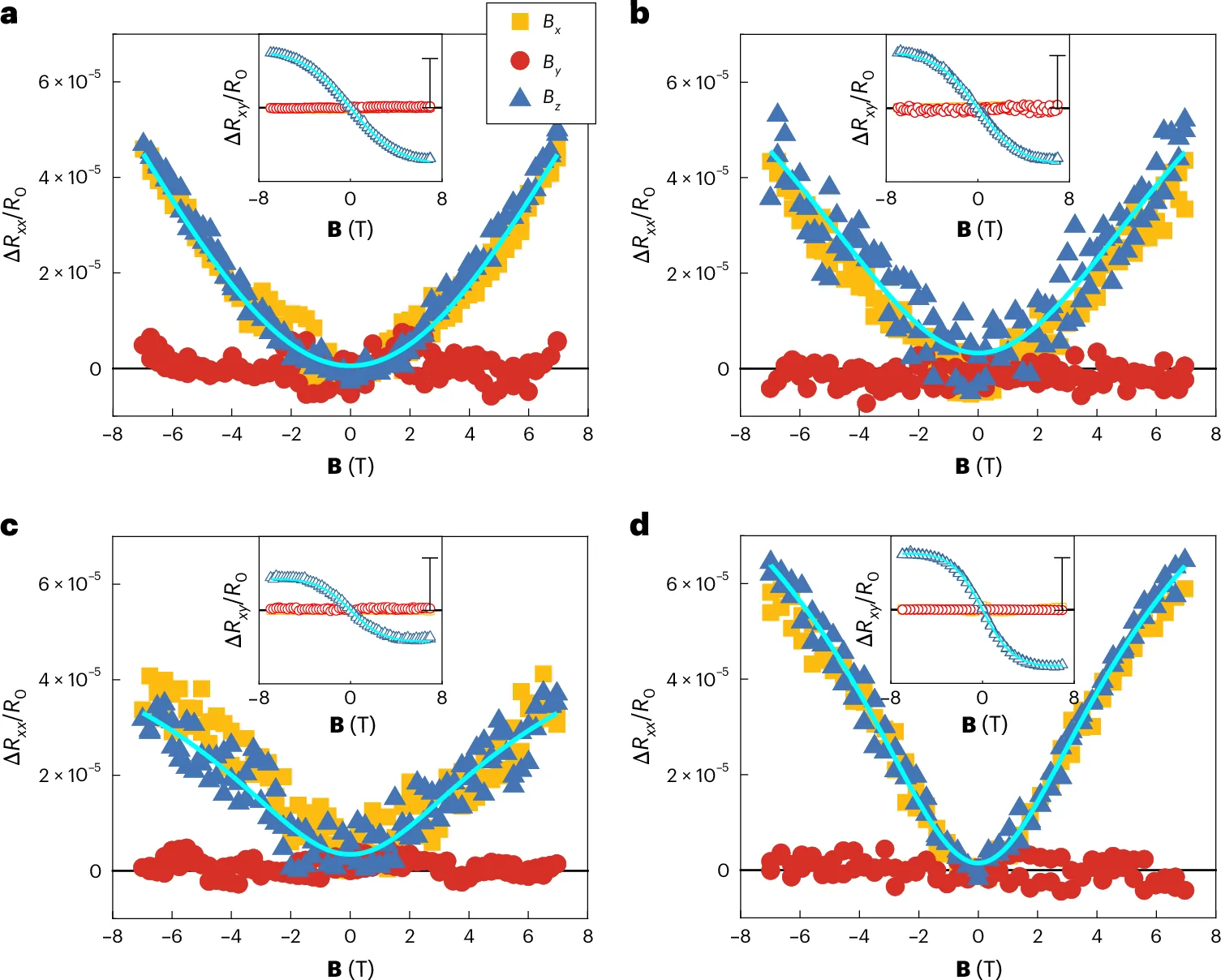
Orbital HMR in Mn thin films with varying crystallinity. **a**–**d**, Longitudinal magnetoresistance $\Delta {R}_{xx}/{R}_{0}$ and transverse magnetoresistance $\Delta {R}_{xy}/{R}_{0}$ (insets) measured at room temperature under magnetic fields oriented along *x* (yellow squares, ${B}_{x}$), *y* (red circles, ${B}_{{y}}$) and *z* (blue triangles, ${B}_{z}$) in epitaxial Mn(001)[110] (**a**), textured Mn(211) (**b**), polycrystalline Mn (**c**) and amorphous Mn (**d**). Solid lines represent fits to the orbital HMR equations (Methods). Source data

Figure 2 shows the magnetotransport signatures of orbital HMR at room temperature for Mn films with various degrees of crystalline order. The magnetoresistance is defined as ${\Delta R}_{xx,xy}/{R}_{0}=[{R}_{xx,xy}\left(B\right)-{R}_{xx,xy}\left(0\right)]/{R}_{xx}\left(0\right)$, where ${R}_{0}$ is the resistance at *B* = 0. In all samples, the longitudinal magnetoresistance ${\Delta R}_{xx}/{R}_{0}$ increases monotonically with the magnetic field when **B** ∥ ${{x}}$, *z* and vanishes for **B** ∥ *y* (∥ **L**). The transverse magnetoresistance (${\Delta R}_{xy}/{R}_{0}$), instead, vanishes for **B** ∥ *x*, *y* and follows a sigmoidal curve for **B** ∥ *z*, consistent with the fingerprints of orbital HMR^17,21^. In fact, when **B** ∥ *z*, the OAM precession generates a net ${{\bf{j}}}_{{\rm{e}}}^{{\rm{r}}}$ ∥ *y*, which induces the transverse HMR (Fig. 1d). The magnitude of the orbital HMR, calculated as ${\Delta R}_{xx}(7\,{\rm{T}})/{R}_{0}$, ranged from ~3.0 to 6.0 × 10^−5^. The HMR signal exhibited nearly isotropic behaviour with respect to the current orientation (*φ* = 0°, 45°, and 90º) in both epitaxial and textured Mn, consistent with a cubic cyrstal structure. The persistence of clear HMR signatures across all structural phases, including amorphous Mn, indicates that orbital currents emerge even in strongly disordered systems in the absence of crystalline order.

The curvature of the HMR depends on Hanle precession, resulting in ${\Delta R}_{xx,xy}\left(B\right)\propto 1/[1+{\left({\omega }_{{\rm{L}}}{\tau }_{{\rm{L}}}\right)}^{2}]$, where ${{\rm{\omega }}}_{{\rm{L}}}={\mu }_{{\rm{B}}}B/\hslash$ is the Larmor frequency of the OAM, *μ*_B_ is the Bohr magnetron and *ℏ* is the reduced Planck constant. The measurements in Fig. 2 show that the magnetic-field-dependent line shape of the HMR evolves with the crystallinity. Crystalline and textured samples display broader Hanle curves, consistent with fast orbital dephasing. By contrast, polycrystalline and amorphous samples exhibit sharper Hanle-like dips, indicative of longer orbital relaxation times. These measurements indicate that orbital dephasing scales with the degree of structural order. This trend can be qualitatively understood by considering the local crystal-field environment governing orbital dynamics: ordered crystalline films induce fast orbital precession and, thus, faster dephasing, whereas structural disorder and hopping tend to average out the crystal field, thus extending the orbital relaxation time. To extract quantitative parameters characterizing orbital generation and relaxation in Mn thin films, we fitted the longitudinal and transverse magnetoresistances using a model of the HMR originally derived for the spin Hall effect^37^. Simultaneous fits of $\Delta {R}_{xx}/{R}_{0}$ and $\Delta {R}_{xy}/{R}_{0}$ yielded two sets of parameters, ${\sigma }_{{\rm{L}}}$ and ${\tau }_{{\rm{L}}}$, which we relate to the degree of static and thermal disorder in our films (Methods).

## Conductivity, orbital Hall conductivity and relaxation versus structural disorder

**Extended Data Fig. 1:**
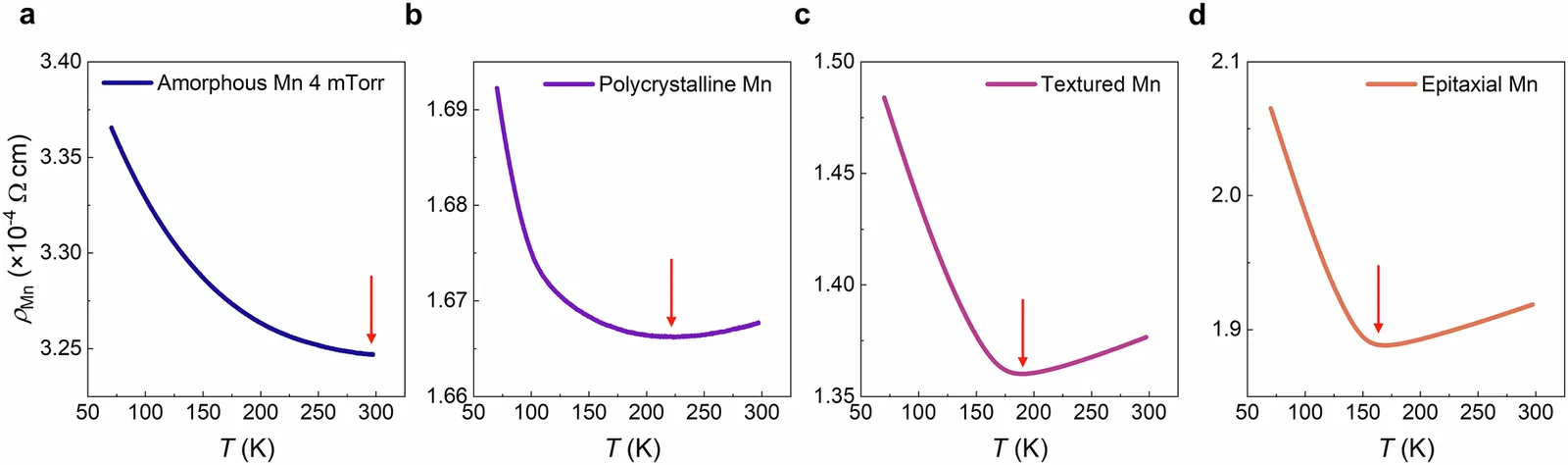
Electrical properties of Mn films. Temperature-dependent resistivity (${\rho }_{{\rm{Mn}}}$) of 10-nm-thick (**a**) amorphous Mn (4 mTorr of Ar sputtering pressure), (**b**) room temperature polycrystalline Mn, (**c**) textured Mn(211), and (**d**) epitaxial Mn(001). The red arrows serve as a guide to the eye to identify the resistivity minima. Source data

The amorphous samples present the lowest conductivity, reaching deep into the bad-metal regime, where the conductivity is activated and determined by phonon-assisted hopping^40^ or disorder-enhanced polaron formation^41^. The resistivity curves of the amorphous Mn films all show negative temperature coefficients of resistance (TCRs), consistent with disorder-dominated charge transport (Extended Data Fig. 1). The polycrystalline (high-temperature-grown), textured and epitaxial films, instead, have positive TCRs at room temperature, as expected for metals in the semi-classical Boltzmann transport regime, and conductivities close to 10^4^ Ω^−1^ cm^−1^. The corresponding resistivities of these films, ranging from 130 μΩ cm to 190 μΩ cm, approach but remain below the Mott–Ioffe–Regel limit ${\rho }_{\mathrm{MIR}}=3\hslash a/{e}^{2}\approx 300$ μΩ cm, which defines the maximum resistivity compatible with coherent quasiparticle transport, where the electron mean free path becomes comparable with the interatomic distance a (ref. ^42^). This behaviour is consistent with the intrinsically high resistivity of α-Mn, even in ordered bulk crystals ($\rho \approx 150$ μΩ cm), making it the only transition-metal element that exhibits resistivity saturation well below room temperature^42^. The unusual transport properties of α-Mn are attributed to its highly complex unit cell, which comprises 58 atoms occupying four inequivalent lattice sites^39^. This structural complexity gives rise to several electronic bands with large effective masses crossing the Fermi level, resulting in pronounced contributions from interband transitions and electron–phonon scattering to the resistivity, beyond semi-classical models^42,43^. Accordingly, epitaxial α-Mn films have a higher resistivity compared with polycrystalline and textured films, which contain other crystal phases, such as γ-Mn, that have a lower bulk resistivity compared with α-Mn^44^.

**Fig. 3:**
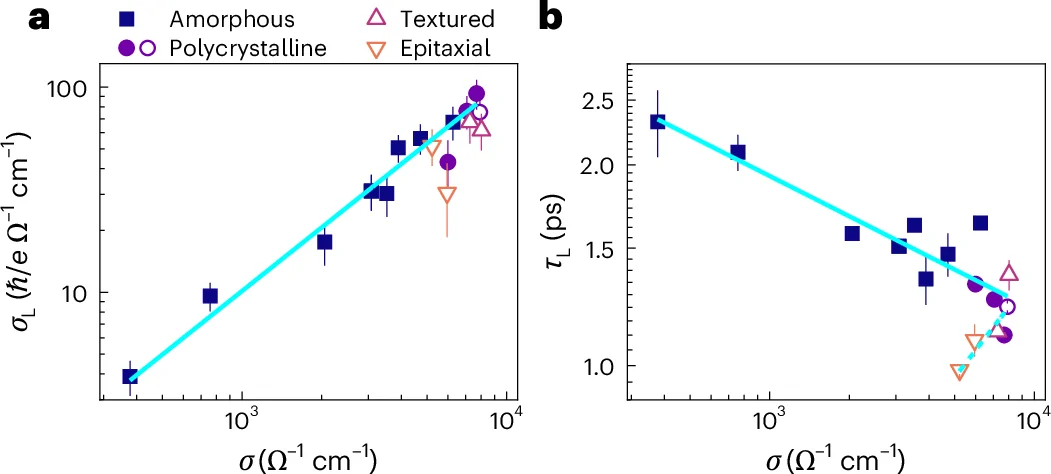
Scaling of orbital Hall conductivity and relaxation time with electrical conductivity in films with different degrees of static disorder. **a**,**b**, Orbital Hall conductivity ${\sigma }_{{\rm{L}}}$ (**a**) and relaxation time ${\tau }_{{\rm{L}}}$ (**b**) as functions of Mn film conductivity *σ* at room temperature. The data points span amorphous (squares), polycrystalline (circles), textured (triangles) and epitaxial (inverted triangles) films. Filled (open) symbols denote films with negative (positive) TCRs at room temperature for epitaxial, textured and high-temperature-grown polycrystalline Mn. Solid cyan lines represent least-squares linear fits of ${\sigma }_{{\rm{L}}}$ and ${\tau }_{L}$ measured for samples with negative TCRs. The dashed line in **b** is a power-law fit of ${\tau }_{{\rm{L}}}$ measured for samples with positive TCRs. All data points were extracted from orbital HMR measurements (Fig. 2). Error bars indicate uncertainties from nonlinear least-squares fits of the HMR and were calculated using standard error propagation. Each data point corresponds to an independent device. Source data

One of our main findings is that ${\sigma }_{{\rm{L}}}$ increases linearly with σ in the amorphous and polycrystalline samples due to thermally assisted conductivity, spanning nearly two orders of magnitude of conductivity at room temperature, as shown Fig. 3a. Despite the diversity of structures and conductivity of these films, ${\sigma }_{{\rm{L}}}$ can be fitted by a simple power law ${\sigma }_{{\rm{L}}}\propto {\sigma }^{1.0\pm 0.2\,}$ (solid line in Fig. 3a), which shows that the orbital Hall effect follows a similar universal scaling as the anomalous Hall and spin Hall effect in the low-conductivity regime ($\sigma \le {10}^{4}\,{\Omega }^{-1}\,{\rm{c}}{{\rm{m}}}^{-1}$). Theory predicts ${\sigma }_{xy}\propto {\sigma }^{\gamma }$ with *γ* = 1.3–1.7 for different hopping processes^45^, and experiments yield *γ* = 1.0–1.8 for different material systems^46,47^. Our data, thus, show that robust orbital generation occurs in strongly disordered films and increases with increasing conductivity across different crystal structures. Moreover, ${\sigma }_{{\rm{L}}}$ deviates from the intrinsic scaling ${\sigma }_{{\rm{L}}}\propto {\sigma }^{0\,}$ predicted for the orbital Hall effect in the good-metal regime, highlighting the strong influence of scattering and calling for further investigations of the orbital Hall effect close to and above the Mott–Ioffe–Regel limit. In this strongly disordered regime, charge transport occurs via thermally activated hopping between localized electron states. The orbital Hall response is, therefore, interpreted as a hopping-mediated transverse accumulation of orbital momenta, analogous to the hopping-induced anomalous and spin Hall effects^45–47^, rather than in terms of the skew or side-jump scattering of Bloch electrons.

Figure 3b provides insight into the orbital relaxation mechanism. In all samples, ${\tau }_{{\rm{L}}}$ was short, varying between 1 ps and 2.5 ps. These values are substantially smaller than the spin relaxation times reported for transition-metal thin films in the diffusive transport regime^48^, indicating that orbital relaxation in Mn is intrinsically faster than spin relaxation. The net decrease of ${\tau }_{{\rm{L}}}$ with increasing crystalline order indicates that the crystal-field-induced precession of OAM is the primary cause of orbital decoherence across different structural phases. Additionally, two different relaxation trends emerge from our analysis. In amorphous and polycrystalline films with activated conductivity (negative TCRs at room temperature), ${\tau }_{{\rm{L}}}$ follows an inverse power-law dependence ${\tau }_{{\rm{L}}}\propto {\sigma }^{-0.20\pm 0.03}$ (solid line in Fig. 3b). By contrast, films with positive TCRs (open symbols in Fig. 3), including epitaxial, textured and high-temperature-grown polycrystalline Mn, show a direct scaling ${\tau }_{{\rm{L}}}\propto {\sigma }^{0.51\pm 0.07}$ (dashed line), consistent with EY relaxation. These different trends indicate a crossover between EY-type relaxation in epitaxial Mn to a different relaxation process in amorphous Mn. Despite the inverse scaling of ${\tau }_{{\rm{L}}}$ versus *σ*, this process is not associated with conventional DP relaxation, as the latter is expected only in the limit of large *σ* (refs. ^34,49^).

## Orbital Hall conductivity and relaxation versus thermal disorder

**Fig. 4:**
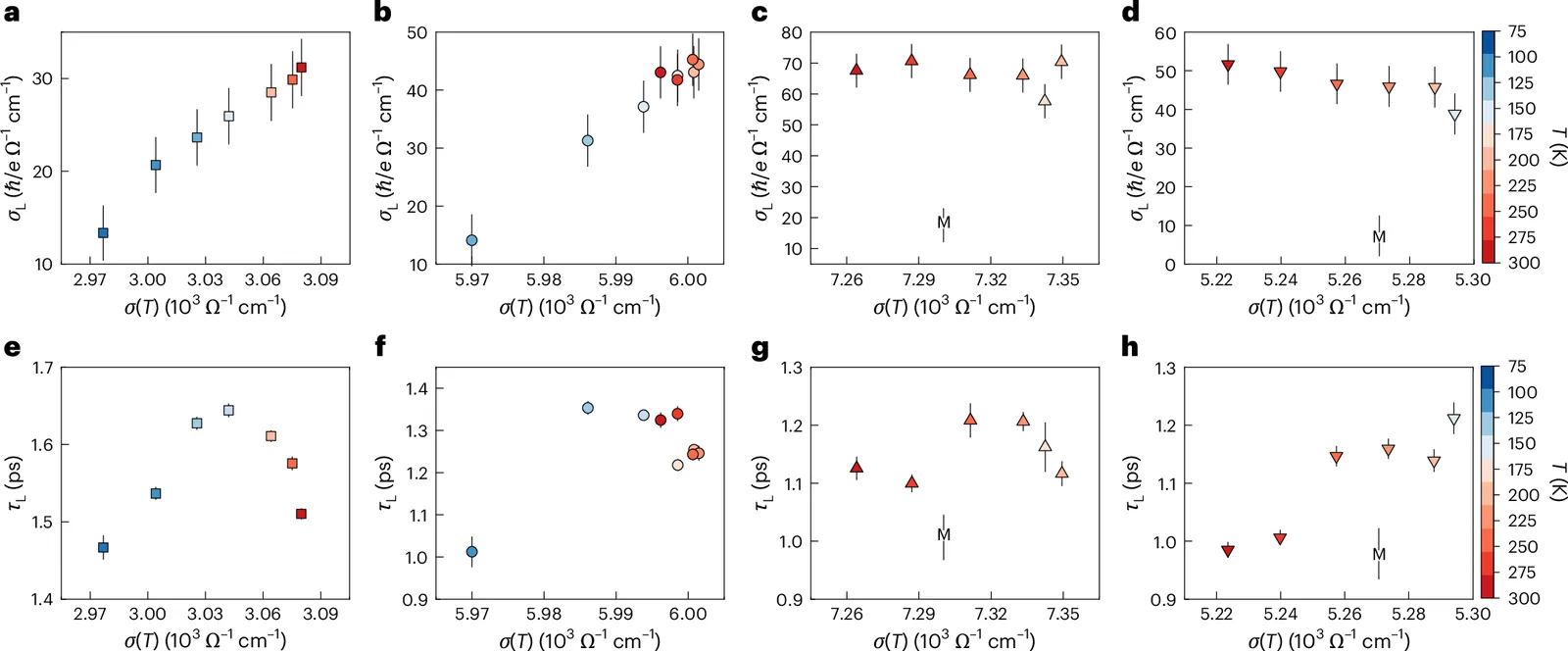
Temperature dependence of orbital Hall conductivity and relaxation time. **a**–**h**, Temperature evolution of the orbital Hall conductivity ${\sigma }_{{\rm{L}}}(T)$ (**a**–**d**) and orbital relaxation time ${\tau }_{{\rm{L}}}(T)$ (**e**–**h**) for amorphous (**a**,**e**), polycrystalline (**b**,**f**), textured (**c**,**g**) and epitaxial (**d**,**h**) Mn films. The colour encodes the temperature from 300 K (dark red) to 75 K (dark blue), as indicated by the colour bar. All data points were extracted from orbital HMR measurements. Data were obtained from measurements on the same device as a function of temperature (*n* = 1 device per dataset). Error bars indicate uncertainties from nonlinear least-squares fits of the HMR and were calculated using standard error propagation. Source data

To better understand orbital relaxation, we compared the temperature dependence of ${\sigma }_{{\rm{L}}}(T)$ and ${\tau }_{{\rm{L}}}(T)$ in Mn films with selected amounts of structural disorder (Fig. 4). Our analysis was restricted to the paramagnetic regime, where the transverse HMR retains its characteristic sigmoidal *B*-field dependence, an established fingerprint of orbital HMR. In contrast to Fig. 3, where *σ* was varied over nearly two orders of magnitude by controlling the static disorder, temperature induces only minor percentage changes in σ(T). Despite this limited conductivity range, both ${\sigma }_{{\rm{L}}}(T)$ and ${\tau }_{{\rm{L}}}(T)$ exhibited substantial changes, indicating that orbital generation and relaxation are highly sensitive to phonon scattering. In amorphous and polycrystalline Mn, ${\sigma }_{{\rm{L}}}(T)$ increased with σ(T) as the temperature rose (Fig. 4a,b). This monotonic trend, also reflected in the gradual progression of data points from blue (low *T*) to red (high *T*), is consistent with the ${\sigma }_{{\rm{L}}}\propto \sigma$ scaling observed at room temperature (Fig. 3a). Together, these results indicate that faster hopping leads to a smaller loss of orbital coherence and larger OAM generation. By contrast, ${\sigma }_{{\rm{L}}}(T)$ in textured and epitaxial Mn films showed a weak temperature dependence, as expected for the intrinsic orbital Hall effect (Fig. 4c,d). At temperatures below ~125 K, however, ${\sigma }_{{\rm{L}}}(T)$ exhibited a sharp drop in both epitaxial and textured α-Mn (points marked by M). This suppression is linked to the onset of antiferromagnetic order, as shown by the emergence of negative and anisotropic magnetoresistance at and below 125 K (Supplementary Note 3). The sudden reduction of ${\sigma }_{{\rm{L}}}(T)$ close to the Néel temperature may reflect the lifting of band degeneracies and lattice distortion that occur in Mn upon spin ordering^39^, as these alter the orbital texture and lead to a loss of symmetry and the suppression of the orbital Hall effect.

The temperature dependence of ${\tau }_{{\rm{L}}}(T)$ also reveals striking differences between disordered and ordered films. In amorphous and polycrystalline Mn, ${\tau }_{{\rm{L}}}(T)$ displayed a non-monotonic behaviour, increasing (decreasing) with σ(T) at low (high) temperature (Fig. 4e,f). Here we argue that the increase of ${\tau }_{{\rm{L}}}(T)$ with σ(T) at low temperature is a signature of on-site orbital relaxation in the low-conductivity regime where thermally activated hopping is slow. In the regime of thermally activated conductivity, the characteristic transport timescale is the hopping time ${\tau }_{{\rm{hop}}}\propto {\sigma }^{-\nu }$ (with ν>0; ref. ^50^), which represents the average localization time of electrons on a given site. A longer ${\tau }_{{\rm{hop}}}$, thus, allows for more effective dephasing of the electron OAM through precession in the local crystal field. Conversely, a shorter ${\tau }_{{\rm{hop}}}$ leads to partial averaging out of the random crystal fields at different sites, thereby increasing ${\tau }_{{\rm{L}}}$, akin to the motional narrowing of spin relaxation in organic semiconductors^51,52^. As the temperature increases, the hopping rate accelerates, and hopping-induced orbital relaxation via mixing of orbital wavefunctions begins to dominate over local dephasing. This crossover produces the observed downturn of ${\tau }_{{\rm{L}}}(T)$ with increasing σ(T) at high temperature, mirroring spin relaxation in disordered materials lacking coherent charge transport^53,54^. This behaviour contrasts sharply with spin and orbital relaxation in good metals, for which a transition from scattering-driven EY relaxation to crystal-field-driven DP dephasing occurs upon increasing the conductivity^34,49^.

In crystalline conductors, the relation between OAM relaxation and conductivity is inverted relative to the hopping regime. The relevant transport timescale is *τ*, the time during which an electron propagates coherently without scattering. Thus, DP dephasing scales as ${\tau }_{{\rm{L}}}^{-1}\propto \tau \propto \sigma$ and is expected to dominate at low temperatures in the high-conductivity limit, whereas EY relaxation scales as ${{\tau }_{{\rm{L}}}^{-1}\propto {\tau }^{-1}}\propto \,{\sigma }^{-1}$ (ref. ^34^). For epitaxial α-Mn (Fig. 4h), we observed an approximately linear increase of ${\tau }_{{\rm{L}}}(T)$ with σ(T), which is indicative of EY-type OAM relaxation. The DP regime could not be reached in our samples due to the intrinsically high resistivity of the crystalline α-Mn phase. In textured Mn (Fig. 4g), ${\tau }_{{\rm{L}}}(T)$ varied weakly with σ(T), indicating a balance between competing temperature-dependent transport mechanisms across grain boundaries and different Mn phases. In both epitaxial and textured Mn films, ${\tau }_{{\rm{L}}}$ sharply decreased below ~125 K with the onset of antiferromagnetic order, concurrently with the suppression of ${\sigma }_{{\rm{L}}}$, confirming that OAM is highly sensitive to magnetic correlations.

## Discussion

Our results provide a comprehensive description of electric-field-induced OAM generation and relaxation in a single-element transition metal with various degrees of static and thermal disorder, ranging from fully amorphous to single-crystalline α-Mn. Finite OAM generation, quantified by ${\sigma }_{{\rm{L}}}$, emerged even in highly disordered samples, which exhibited hopping-like conductivity, and it increased proportionally with *σ* in amorphous and polycrystalline Mn. This scaling shows that the orbital Hall effect follows a universal behaviour analogous to the anomalous Hall effect and spin Hall effect in the bad-metal regime. Phonon scattering enhances ${\sigma }_{L}$ in amorphous Mn, consistent with a hopping-mediated orbital Hall effect, whereas it has little influence on ${\sigma }_{{\rm{L}}}$ in textured and epitaxial Mn, as expected for the intrinsic orbital Hall effect of crystalline metals. OAM relaxation, characterized by ${\tau }_{{\rm{L}}}$, is generally fast and negatively impacted by crystalline order. It ranged from to 2.5 ps in amorphous Mn to 1 ps in epitaxial Mn at room temperature. The relaxation dynamics differ markedly between films exhibiting hopping-like and metallic conductivity. In amorphous and polycrystalline films, where conduction is thermally activated, electron localization promotes on-site OAM dephasing in local crystal fields at low temperature. As the temperature and hopping rate increase, ${\tau }_{{\rm{L}}}$ initially grows due to motional narrowing in a random crystal field and subsequently decreases once non-conserving OAM hopping processes dominate. In textured and epitaxial Mn, OAM relaxation gradually transitions to EY-type dynamics, where ${\tau }_{{\rm{L}}}$ increases with *σ*, in agreement with the recent theory of orbital transport in crystalline metals^34^.

The generation and transport of non-equilibrium OAM are, therefore, universal processes that do not require long-range translational symmetry. However, efficient and competing channels are available for OAM relaxation, even in systems lacking spin–orbit coupling, well-defined symmetry and a periodic crystal-field potential. The opposite trends of ${\sigma }_{{\rm{L}}}$ and ${\tau }_{{\rm{L}}}$ with static crystalline order show that OAM generation induced by the orbital texture competes with crystal-field-induced relaxation. As a result, OAM accumulation, proportional to ${\sigma }_{{\rm{L}}}\sqrt{{\tau }_{{\rm{L}}}}$, may be enhanced in disordered conductors. Beyond fundamental insights, our findings show that orbitronic functionalities extend to non-crystalline and technologically relevant disordered materials, thus establishing OAM as a structurally tolerant, tunable channel for next-generation spin–orbitronic applications.

## Methods

### Sample preparation

α-Mn thin films with systematically varied crystallinity were fabricated using two distinct deposition techniques. Epitaxial Mn(001) and textured Mn(211) films were grown by MBE on MgO(001) and MgO(110) substrates, respectively. Before deposition, the substrates were precooled to −180 °C and transferred to the deposition chamber within 5 min, which reduced the roughness and increased the crystallite size. The substrates were degassed in an ultra-high vacuum at about 500 °C before Mn was deposited. Mn films were subjected to post-growth annealing up to about 300 °C to favour crystallization and smoothening. The base pressure of the MBE chamber was maintained below 2 × 10^−9^ mbar during growth. Low-temperature (high-temperature) polycrystalline Mn films were deposited on Si/SiN by precooling (preheating) the substrates to −180 °C (500 °C) and transferring them to the deposition chamber within 5 min. All the polycrystalline Mn films were annealed up to about 100 °C after growth. Amorphous Mn films were deposited by magnetron sputtering on Si/SiN substrates in an Ar atmosphere at pressures ranging from 2 mTorr to 40 mTorr. Increasing the Ar pressure led to increased film disorder and surface roughness, which gave us controlled access to different disorder regimes. To prevent surface contamination of Mn, MBE-grown films were protected by an Al capping layer (~3–4 nm) deposited in situ immediately after growth, which was subsequently oxidized under ambient conditions. After device fabrication, further O_2_ treatment was applied to ensure complete oxidation of the Al layer to form a stable AlO_*x*_ cap. For sputtered Mn films, an ~8-nm SiN capping layer was deposited by radio-frequency sputtering at an Ar pressure of 10 mTorr immediately after Mn growth. All films were grown with a nominal thickness of 10 nm unless otherwise specified. The structural properties of the films were characterized in situ using RHEED and ex situ using XRD and a superconducting quantum interference device (Supplementary Notes 1 and 2).

### HMR measurements

For electrical transport studies, all Mn films were patterned into Hall bar devices using photolithography and Ar ion milling. The Hall bars were oriented along specific crystallographic axes of the underlying Mn films. For the epitaxial and textured samples, the current injection direction was aligned along the in-plane [110] and $\left[\bar{1}11\right]$ directions, respectively, as determined by substrate orientation and verified by RHEED. Magnetoresistance measurements were carried out in a variable-temperature cryostat from 10 K to 300 K. Longitudinal and transverse resistance components were recorded under magnetic field sweeps up to ±7 T applied along three orthogonal axes (*x, y, z*). A low-frequency a.c. (100 μA at 17 Hz) was applied using a lock-in technique to minimize thermal drift. Temperature-dependent measurements were performed during heating. At each set temperature, the sample was stabilized for at least 10 min with no current applied and 20 min with current before starting the measurement to ensure thermal equilibrium. Magnetic-field sweeps were performed in both directions to verify the absence of thermal drift.

The longitudinal HMR and transverse HMR were fitted using the following equations^17,21,37^:

1 $$\frac{\Delta {R}_{xx}}{{R}_{0}}=2{\left(\frac{{\sigma }_{{\rm{L}}}}{\sigma }\right)}^{2}\left[\frac{{\lambda }_{{\rm{L}}}}{t}\tanh \left(\frac{t}{2{\lambda }_{{\rm{L}}}}\right){\mathfrak{-}}{\mathfrak{R}}e\left\{\frac{\varLambda }{t}\tanh \left(\frac{t}{2\varLambda }\right)\right\}\right],$$

2 $$\frac{\Delta {R}_{xy}}{{R}_{0}}=2{\left(\frac{{\sigma }_{{\rm{L}}}}{\sigma }\right)}^{2}\frac{w}{l}{\mathfrak{I}}m\left\{\frac{\varLambda }{t}\tanh \left(\frac{t}{2\varLambda }\right)\right\},$$

where *t* is the film thickness, *l* and *w* are the length and width of the Hall bars, *σ* is the electrical conductivity and ${\varLambda }^{-1}=\sqrt{1/{\lambda }_{{\rm{L}}}^{2}{+}{\rm{i}}{/\lambda }_{{\rm{m}}}^{2}}$ is a characteristic length scale that captures the combined effects of orbital diffusion and Hanle precession of the current-induced OAM. The orbital diffusion length is given by ${\lambda }_{{\rm{L}}}=\sqrt{D{\tau }_{{\rm{L}}}}$, and the precessional length is defined as ${\lambda }_{{\rm{m}}}=\sqrt{D/{\omega }_{{\rm{L}}}}$, where *D* is the orbital diffusion coefficient. Equations (1) and (2) contain three free parameters: ${\sigma }_{{\rm{L}}}$, ${\tau }_{{\rm{L}}}$ and *D*. To further reduce the number of fitting parameters, we used the Einstein relation for metals ${D}={a}\sigma$ (ref. ^55^), where the proportionality constant *a* is a global parameter used to fit the magnetoresistance of each sample at different temperatures. The fits of $\Delta {R}_{xx}/{R}_{0}$ and $\Delta {R}_{xy}/{R}_{0}$ thus yield two sets of parameters, ${\sigma }_{L}$ and ${\tau }_{L}$, which we relate to the degree of static and thermal disorder in our films.

We note that this analysis is based on the correction of the resistivity tensor due to orbital accumulation at the edges of the sample within a drift–diffusion approach analogous to the spin HMR^37^. The validity of drift–diffusion models extends also to systems with hopping-like conductivity, provided that a local chemical potential and a continuous carrier density can be defined, and where the applied fields and carrier density vary slowly compared with the correlation length of disorder, leading to Ohmic transport^56^. These conditions are fulfilled in all our samples due to their high electron density (~5–30 × 10^22^ cm^−3^) and short intersite spacing. We also note that drift–diffusion models are commonly employed to analyse charge^57–59^ and spin^60–63^ transport and relaxation in disordered materials.

### STEM

Cross-sectional specimens for our STEM investigations were prepared using a focused gallium (Ga^+^) ion beam in an FEI Helios 660 G3 UC dual-beam system operated at acceleration voltages of 30 kV and 5 kV. Before milling, protective carbon (C) and platinum (Pt) layers were deposited to minimize ion-beam-induced damage. HAADF-STEM imaging was performed using an FEI Titan Themis microscope operated at 300 kV and equipped with a CEOS DCOR spherical aberration probe corrector. HAADF-STEM images were acquired using a probe semi-convergence angle of 24 mrad and an annular detector semi-collection angle of 90–200 mrad.

## Online content

Any methods, additional references, Nature Portfolio reporting summaries, source data, extended data, supplementary information, acknowledgements, peer review information; details of author contributions and competing interests; and statements of data and code availability are available at https://doi.org/10.1038/s41567-026-03334-z.

## Supplementary information

Supplementary Information Supplementary Figs. 1–12, Table 1 and Notes 1–4.

Peer Review File

## Source data

Source Data Fig. 1 XRD intensity as a function of 2*θ* for epitaxial and textured Mn films.

Source Data Fig. 2 Orbital HMR as a function of magnetic field with fitted curves for Mn thin films with various degrees of crystallinity.

Source Data Fig. 3 Orbital Hall conductivity and relaxation time as a function of electrical conductivity with fitted curves for Mn films with various levels of static disorder.

Source Data Fig. 4 Orbital Hall conductivity and relaxation time as a function of electrical conductivity, with temperature as a tuning parameter, for Mn thin films.

Source Data Extended Data Fig. 1 Electrical resistivity as a function of temperature for Mn films with various degrees of crystallinity.

## Data Availability

The data that support the findings of this study will be made available on the ETH Research Collection repository (https://doi.org/10.3929/ethz-c-000785607). Source data are provided with this paper.

## Code availability

No custom code was used in this study.
