# Supplementary material for: Orbital Hall conductivity and relaxation in thin films with variable disorder
Source: Nat Phys. 2026 Jun 15;22(8):1238–44. doi: 10.1038/s41567-026-03334-z (PMC13731138; doi:10.1038/s41567-026-03334-z)
Supplement: Supplementary file 1 [file 41567_2026_3334_MOESM1_ESM.pdf]

nature physics

Supplementary information

https://doi.org/10.1038/s41567-026-03334-z

Orbital Hall conductivity and relaxation in thin films with variable disorder

In the format provided by the authors and unedited

Supplementary Note 1. Sample growth and characterization

X-ray diffraction (XRD) and reflection high-energy electron diffraction (RHEED) of textured Mn films

The crystalline quality of the MBE-grown films was assessed by in situ RHEED and ex situ XRD. The analysis of the single-crystal epitaxial α-Mn layers is reported in the main text. Details of the phase analysis of the textured Mn films are reported below.

We performed *ex situ* XRD $\theta/2\theta$ scans, which clearly show that textured 10- and 20-nm-thick Mn films exhibit multi-phase growth (α- plus γ-phase) (see **Fig. 1b**). The diffractograms present two Mn peaks located at 2θ = 49.876° and 78.485° which we attribute to the (422) α-Mn and the (220) γ-Mn peaks, respectively [1,2]. Note that, to account for possible measurement errors due to sample misalignment, the XRD 2θ angle was calibrated from the (220) MgO substrate peak which, according to the MgO bulk lattice constant a = 4.212 Å issued by the substrate provider (equivalent to an interplanar distance of $d_{220} = a/(2\sqrt{2})$ = 1.489 Å), must be centered at 2θ = 62.306°. A diffraction peak coming from the substrate overlaps with the one from (422) α-Mn. Therefore, to perform a semi-quantitative phase analysis of Mn(10 nm), after removing the respective baselines, we subtracted the diffractogram of the MgO(110) reference (as received) to the one of MgO(110)/Mn(10 nm) (see **Fig. S1a**). We then fitted the residual Mn peaks using Lorenz functions and, from the peak center positions, we calculated a lattice constant of 8.960 Å for α-Mn and of 3.444 Å for γ-Mn. The latter value is in excellent agreement with the calculated lattice constants of non-magnetic face-centered cubic (fcc) γ-Mn (3.47 Å in [3] and 3.50 Å in [4]).

γ-Mn is one of the high-temperature Mn allotropes, which has a fcc crystal structure and can be stabilized by heating the room-temperature α-phase above 1368 K [5]. γ-Mn can be (meta-)stabilized at room temperature via rapid quenching of appropriate Mn-based alloys [6], e.g., Mn-Cu [7] and Mn-Fe [8]. These solute-stabilized alloys can distort into a face-centered tetragonal structure (fct) [9,10], which stems from the onset of magnetism [11]. Another method to stabilize γ-Mn at room temperature is by epitaxial growth on suitable substrates, e.g., fct γ-Mn on Cu(100) [12], fcc or fct γ-Mn on Cu_3Au(100) [13,14], and fcc γ-Mn on GaAs [15], the latter having a lattice constant of 3.62 Å. Calculated diffractograms for room temperature γ-Mn provided by the Powder Diffraction File (PDF) database are based on fct crystal structures retrieved from electrolytic Mn deposits [16] or Mn-Fe alloys with small amount of Cu or C impurities [17], presenting larger lattice constants than the high temperature fcc γ-Mn (a = 3.77(2) Å, c = 3.533(2) Å in [16] and a = 3.796 Å, c = 3.592 Å in [17]). The larger lattice constants determine a shift of the (220) γ-Mn peak to smaller 2θ angles (70.606° in [16] and 70.051° in [17]) which could explain why we did not find any match with reference diffractograms in PDF database.

To continue with the analysis, we used the full width at half maximum (FWHM) obtained from the Lorenz fits to estimate the out-of-plane crystallite size using the Scherrer equation (with Scherrer constant set to 0.94), obtaining 11.5 nm for α-Mn and 9.2 nm for γ-Mn. Finally, we attempted estimating the relative weight fractions of the two crystalline phases using the relative peak heights normalized by the respective peak intensities provided in reference spectra, obtaining that γ-Mn constitutes approximately 16% of the film. We highlight that this

approach provides only semi-quantitative results since it assumes that the two reference spectra have comparable normalization factors which is, in fact, incorrect. Given the reduced symmetry (higher number of inequivalent diffracting planes) of the complex body-centered cubic (bcc) structure of α-Mn compared to the fcc γ-Mn, the weight of the α-phase is likely overestimated. At the same time, the high roughness of the γ-phase could induce a sharpening of the (220) XRD peak, leading again to an overestimation of the weight (as well as of the out-of-plane crystallite size).

To corroborate these findings, we performed *in situ* RHEED of Mn(20 nm) which, contrary to Mn(10 nm), presents additional diffraction spots generated by the secondary phase of Mn. These spots are superimposed on the elongated diffraction streaks from α-Mn(211), which clearly indicates the crystal texture of the film. The absence of the secondary spots in Mn(10 nm) (see **Fig. 1a**), suggests that the latter consists primarily of (211)-oriented α-Mn crystallites, consistent with the semi-quantitative XRD phase analysis. The RHEED patterns of the MgO(110) substrate before Mn growth and of the 20-nm-thick Mn film after the post-deposition annealing are shown in **Fig. S1b**. From the vertical spacing between the secondary reflexes along MgO$\langle\bar{1}10\rangle$, we estimated an out-of-plane interatomic distance of $d_\perp \approx (2.39 \pm 0.44)$ Å which, for a (110)-oriented fcc crystal, corresponds to a lattice constant of $d_\perp\sqrt{2} \approx (3.38 \pm 0.62)$ Å. This value is in line with the lattice constant estimated from the XRD analysis, within the uncertainty band. Note that the uncertainty in the $d_\perp$ value comes from the broadening of the secondary reflexes. From the symmetry of the secondary diffraction spots (with respect to the azimuthal rotation of the electron beam) we can reconstruct a surface unit cell having fourfold symmetry, which is in contrast with the twofold symmetry of the surface unit cell expected for γ-Mn(110). This discrepancy can be possibly resolved if we consider the presence of crystal twinning. Furthermore, from the lack of elongation of the secondary reflexes (which resemble 3D transmission spots), we can conclude that the γ-Mn(110) crystallites present high surface roughness.

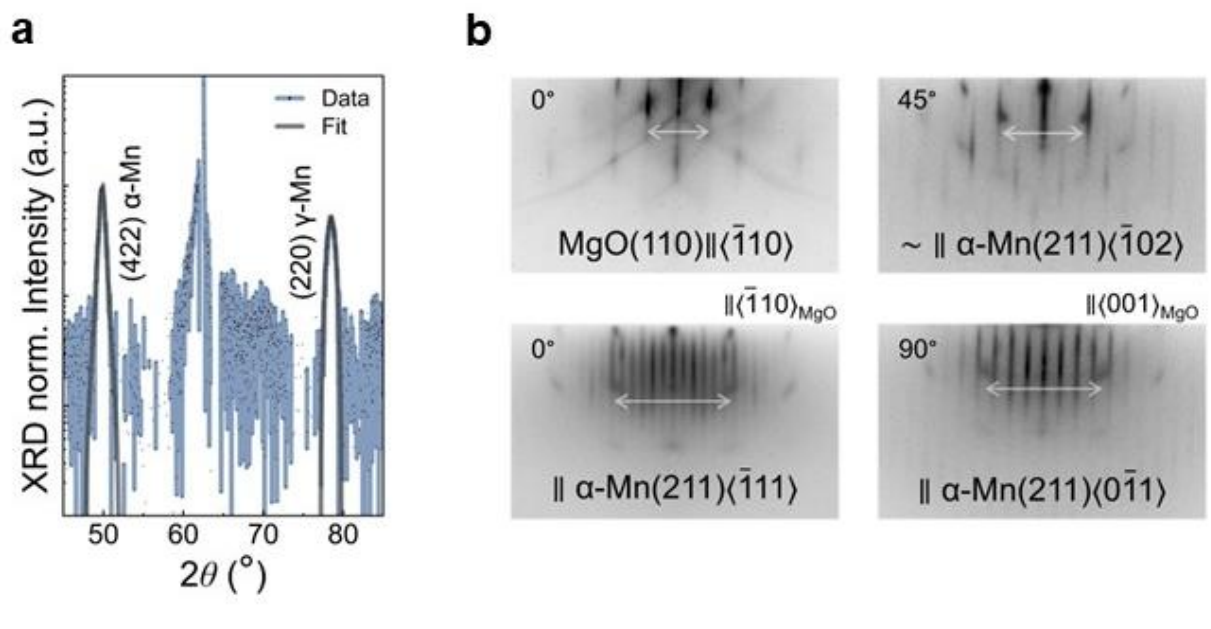

Figure S1 | Structural characterization of textured Mn on MgO(110). a, XRD *θ-2θ* scan of MgO(110)/Mn(10 nm) after subtraction of the MgO(110) reference diffractogram. The grey continuous lines are Lorenz fits of the residual diffraction peaks. **b**, RHEED patterns of the MgO(110) prior to Mn deposition and of the 20-nm-thick textured Mn film with the electron beam of energy 30 keV oriented along different crystallographic axes of Mn (with reference to α-Mn(211)). The number of degrees in the images indicates the azimuthal rotation of the sample during the RHEED acquisition.

Atomic force microscopy (AFM)

We characterized the topography of 10-nm-thick uncapped Mn films of different crystal qualities using ex situ atomic force microscopy (AFM), as presented in **Fig. S2**. The measured root-mean-square (RMS) roughness values from the topography images are $R_q = (0.19 \pm 0.03)$ nm for epitaxial Mn (**Fig. S2a**), (0.35 ± 0.05) nm for polycrystalline Mn (**Fig. S2b**), and (0.79 ± 0.02) nm for amorphous Mn deposited at an Ar sputtering pressure of 6 mTorr (**Fig. S2c**). These results indicate a clear trend of decreasing surface roughness with increasing crystalline order. The corresponding amplitude or deflection images in **Figs. S2d-f** further highlight the differences in surface morphology among the samples.

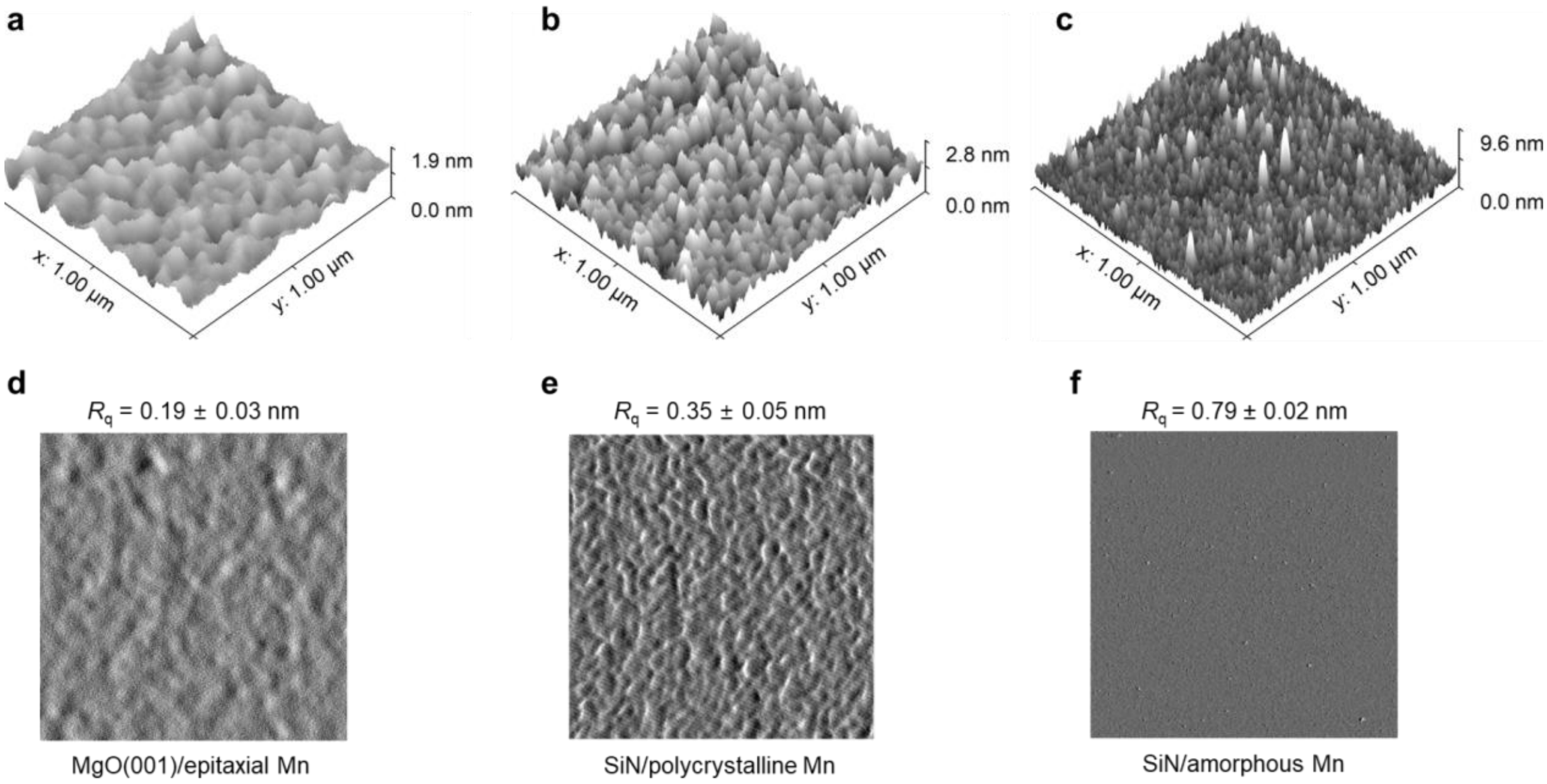

Figure S2 | Structural characterization of uncapped Mn films using AFM. a-c, Topography and **d-f**, amplitude images of 10-nm-thick (a,d) (001) epitaxial, (b,e) polycrystalline, and (c,f) amorphous (4 mTorr of Ar sputtering pressure) Mn films. The root mean square roughness of the films was extracted from the (1×1) μm^2 topography images.

In addition, the AFM analysis reveals that the RMS roughness of amorphous Mn films systematically increases with Ar sputtering pressure, as shown in **Fig. S3**. We note that all AFM measurements were performed immediately after exposure of the film to ambient atmosphere, as oxidation of Mn upon air exposure can influence the surface morphology. The data point at 40 mTorr shows a somewhat lower RMS roughness compared to the films deposited at 10-30 mTorr. This may arise from a non-representative scanned area, given that the corresponding conductivity value remains consistent with the overall trend (see **Fig. S4a**).

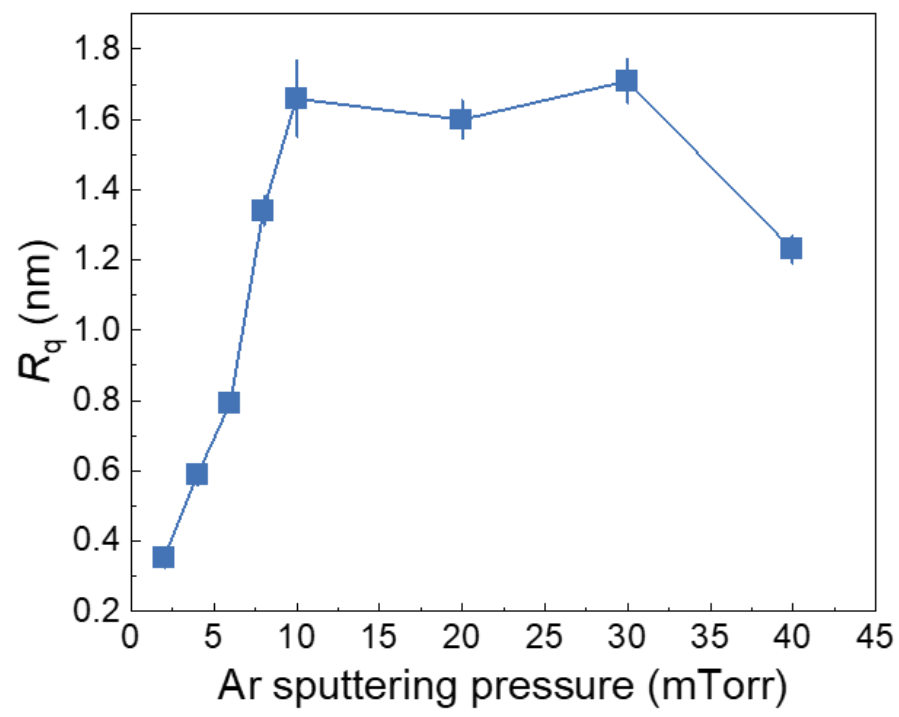

Figure S3 | Root-mean-square roughness R_q of amorphous Mn(10 nm) films as a function of Ar sputtering pressure. Error bars represent standard deviation from n = 5 measurements taken at different locations on the same sample.

Transmission electron microscopy (TEM)

Cross-sectional high-angle annular dark-field scanning transmission electron microscopy (HAADF-STEM) provides real-space confirmation of the structure of Mn films. **Figure S4** shows representative large-, medium-, and small-scale images of epitaxial, polycrystalline, and amorphous films, together with the corresponding Fourier transforms (FTs) of the medium-scale images.

In the epitaxial film, atomic-resolution HAADF-STEM images and corresponding FTs reveal a well-ordered lattice viewed along the α-Mn [011] zone axis, consistent with a film oriented with the [001] direction out of plane. These observations independently confirm the single-crystalline α-Mn phase identified by RHEED and X-ray diffraction.

In the polycrystalline film, cross-sectional images show a granular microstructure composed of crystalline grains with lateral dimensions larger than the film thickness, separated by well-defined grain boundaries. High-resolution imaging of representative grains exhibits lattice periodicities consistent with α-Mn (see, e.g., a grain imaged along the α-Mn [111] direction). The corresponding FTs display well-defined diffraction spots from individual grains with multiple discrete orientations. The presence of high-order diffraction spots indicates long-range crystalline order within each grain.

In the amorphous films, cross-sectional images evidence the absence of long-range crystalline order. The FTs of these films are characterized by a broad diffraction halo at the primary reflection order and negligible secondary reflections, consistent with an amorphous-like structure. Distinct nanoscale correlation structures are observed within the amorphous films depending on the Ar sputtering pressure. The 4 mTorr film shows a continuous diffuse diffraction halo and relatively uniform nanometric contrast, indicating a relatively narrow and homogeneous distribution of correlation lengths below ~4 nm. HAADF-STEM images show that the correlation length is about 1 nm in most of the film. In contrast, the 40 mTorr film exhibits a spotty diffraction halo and heterogeneous nanocrystalline contrast spanning ~1–10 nm, consistent with a broader distribution of structural correlation lengths and increased microstructural disorder.

Taken together, TEM establishes a consistent structural hierarchy across the Mn films: epitaxial films are single-crystalline α-Mn; polycrystalline films consist of multiphase Mn grains including α-Mn; and amorphous films comprise nanostructurally disordered Mn with pressure-dependent correlation length. These structural trends are fully consistent with the diffraction and transport-derived disorder metrics discussed here and in the main text.

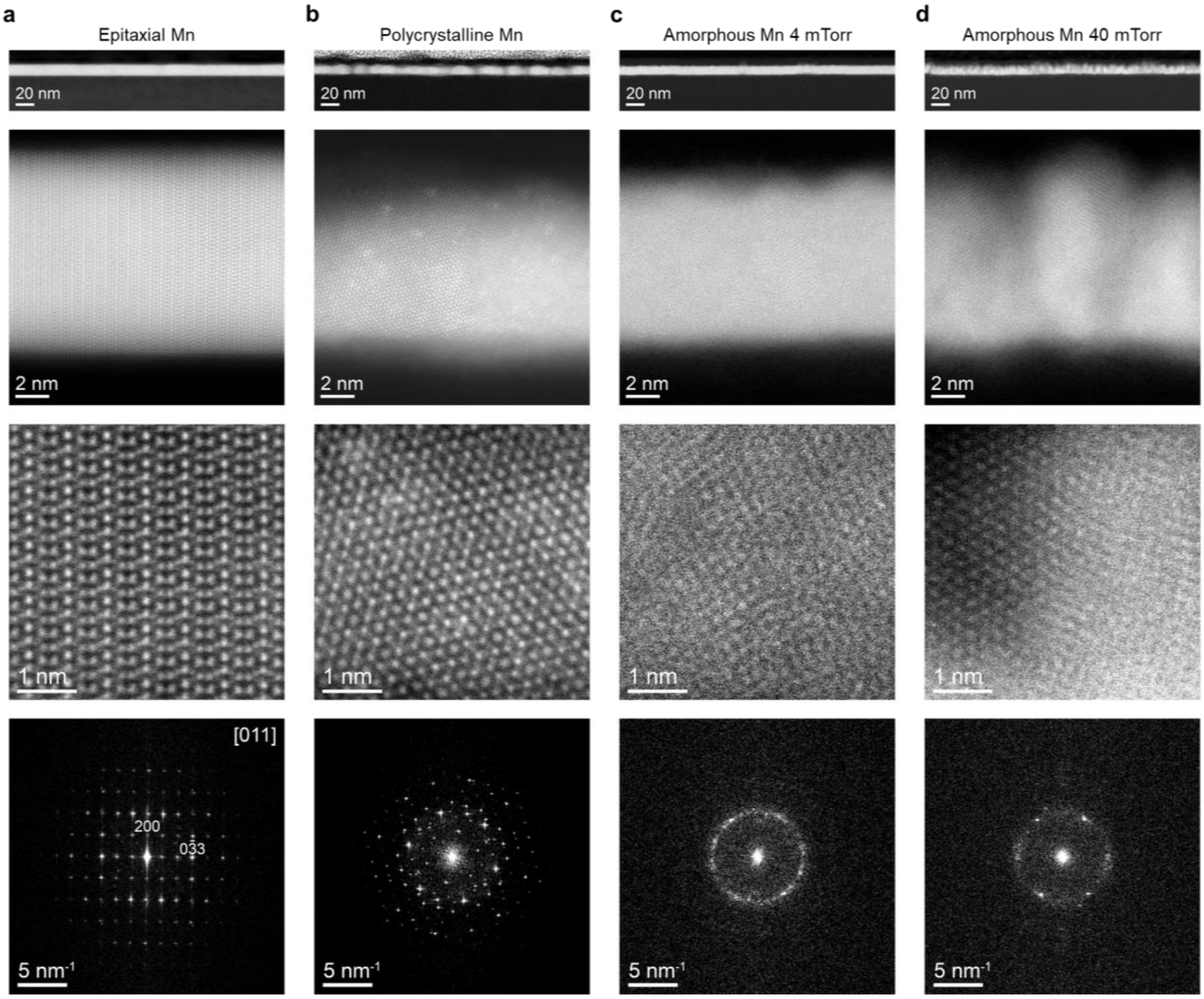

Figure S4 | Transmission electron microscopy of Mn thin films with varying structural order. From top to bottom: low-magnification HAADF-STEM images providing an overview of the Mn films; medium-magnification images highlighting microstructural details; atomically resolved images showing structural features at the atomic scale; Fourier transforms of the medium-magnification images. **(a)** Epitaxial α-Mn, **(b)** polycrystalline Mn, **(c)** nanocrystalline-amorphous Mn grown at 4 mTorr, and **(d)** 40 mTorr.

Ar sputtering-pressure dependent electrical properties of amorphous Mn films

To further demonstrate how sputtering pressure tunes microstructural disorder and electrical transport, we report electrical conductivity and resistivity measurements as a function of Ar pressure, thickness, and temperature. **Figure S5a** shows that the increase in pressure is accompanied by a decrease in film conductivity, consistent with enhanced atomic-scale disorder, surface roughness, and overall reduced metallicity. The thickness dependence of the resistivity (**Fig. S5b**) exhibits a pressure-dependent critical thickness above which the resistivity saturates, indicating a crossover from thickness-limited transport to a regime governed by intrinsic microstructural disorder. The shift of this critical thickness with sputtering pressure shows that the effective disorder correlation length relevant for charge transport is tuned by the growth conditions. Temperature-dependent resistivity further corroborates this interpretation. The low-pressure film (**Fig. S5c**) shows decreasing resistivity and a temperature coefficient of resistance (TCR) approaching zero near room temperature, consistent with a crossover from localized to delocalized charge carrier dynamics. In contrast, the high-pressure film (**Fig. S5d**) retains a large resistivity and negative TCR over the entire temperature range, indicating persistent hopping-dominated transport.

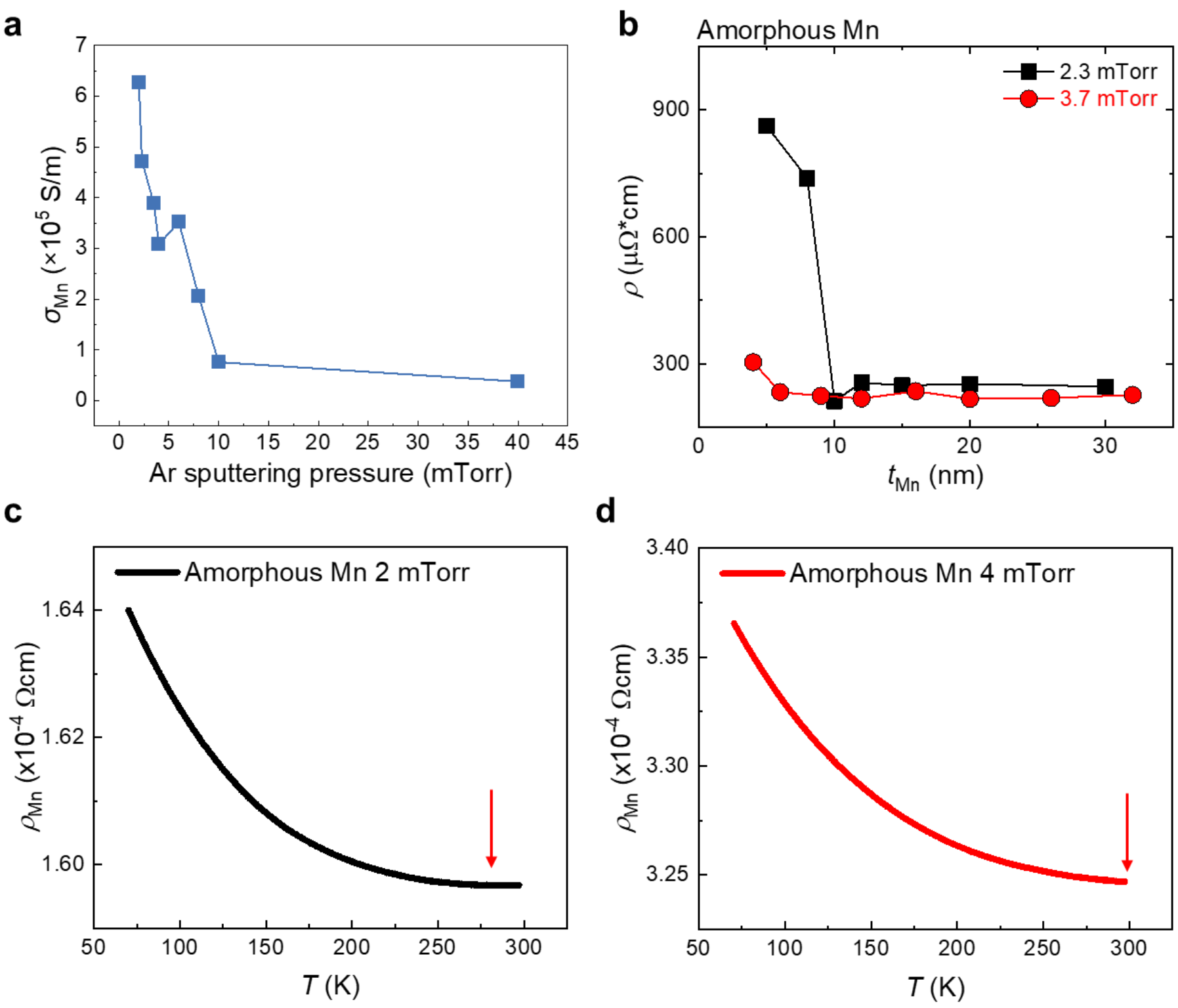

Figure S5 | Ar pressure-dependent electrical properties of amorphous Mn. a, electrical conductivity σ_{Mn} of 10-nm-thick sputtered Mn as a function of Ar pressure. **b**, Longitudinal resistivity ρ as a function of Mn thickness t_{Mn} for films grown at 2.3 and 3.7 mTorr. **c**, ρ(T) for the 10-nm-thick film grown at 2 mTorr. **d**, ρ(T) for the 10-nm-thick film grown at 4 mTorr.

Supplementary Note 2. Magnetic properties of Mn films

We investigated the temperature-dependent magnetic properties of a 10-nm-thick epitaxial Mn film grown on 5×5 mm² MgO(001) substrate using superconducting quantum interference device (SQUID) magnetometry. **Figure S6** presents the magnetic moment as a function of the in-plane magnetic field (applied along Mn[110]||MgO[100]) measured at different temperatures. The dataset includes measurements from the MgO(001)/Mn(10 nm) sample (black curves), a bare MgO(001) substrate as a reference (red curves), and the difference between the two (blue curves). The uncertainty of the difference signal was obtained by propagating the errors of the MgO/Mn and bare MgO measurements as $\Delta M = \sqrt{(\Delta x)^2 + (\Delta y)^2}$, where Δx and Δy denote the uncertainties of the magnetic moments of the MgO/Mn sample and MgO substrate, respectively. The bare substrate displays a small residual magnetic moment, attributable to dilute magnetic impurities likely introduced during the substrate manufacturing process. Notably, this signal increases significantly at 5 K, which can be attributed to the enhanced response of paramagnetic or superparamagnetic impurities at low temperature due to the suppression of thermal fluctuations.

At room temperature, the Mn film shows paramagnetic behaviour, as evidenced by the absence of magnetic hysteresis and the low saturation magnetic moment of $(1.9 \pm 0.5) \times 10^{-9}$ Am^2. This value is considerably smaller than that of a (001)-oriented Mn(10 nm)/Fe(5 nm) reference bilayer grown on MgO(001), which exhibits a saturation moment of $197 \pm 1 \times 10^{-9}$ Am^2. Upon cooling, the saturation magnetic moment of the Mn film decreases progressively and vanishes near 100 K, consistent with a paramagnetic-to-antiferromagnetic phase transition. This transition temperature aligns closely with the Néel temperature of bulk α-Mn (95 K), confirming that the epitaxial Mn layer grows in the α phase. This conclusion agrees with electron and X-ray diffraction measurements (see **Fig. 1a and 1b**). Additionally, the onset of negative anisotropic magnetoresistance observed below 100 K (see Supplementary Note 2) further corroborates the emergence of antiferromagnetic ordering in our epitaxial Mn film.

To further provide an independent verification of the magnetic state in polycrystalline Mn, we performed additional SQUID magnetometry measurements on a 10 nm polycrystalline Mn film grown on Si_3N_4 (**Fig. S7**) and directly compared the results with epitaxial α-Mn. The epitaxial Mn film exhibits a clear paramagnetic signal at room temperature that progressively decreases upon cooling and vanishes near the Néel temperature, consistent with the onset of AFM order. In contrast, the polycrystalline Mn film shows no detectable temperature-dependent magnetic signal between 10 K and 300 K, with the measured moment remaining below ~1 μemu, close to the instrumental resolution limit.

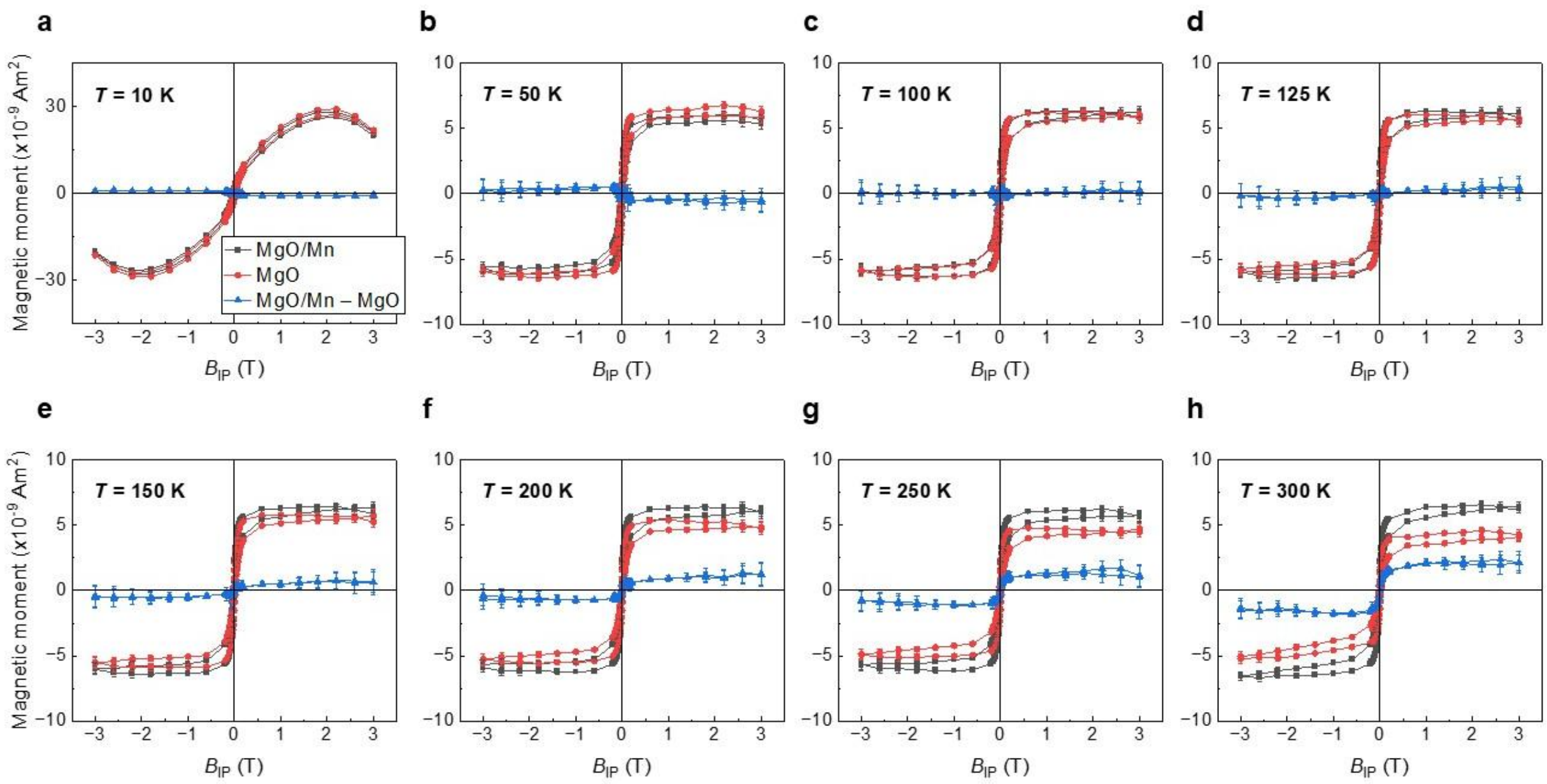

Figure S6 | Magnetic characterization of epitaxial Mn using SQUID. a-h, Magnetization curves (magnetic moment vs. in-plane magnetic field B_{IP}) measured with B_{IP} applied along MgO[100]‖Mn[110] for 10-nm-thick epitaxial Mn(001) single layer (black curves), MgO(001) substrate reference (red curves), and their difference (blue curves). Different panels correspond to different temperatures. Error bars of the Mn signal correspond to propagated uncertainties from MgO/Mn and MgO measurements.

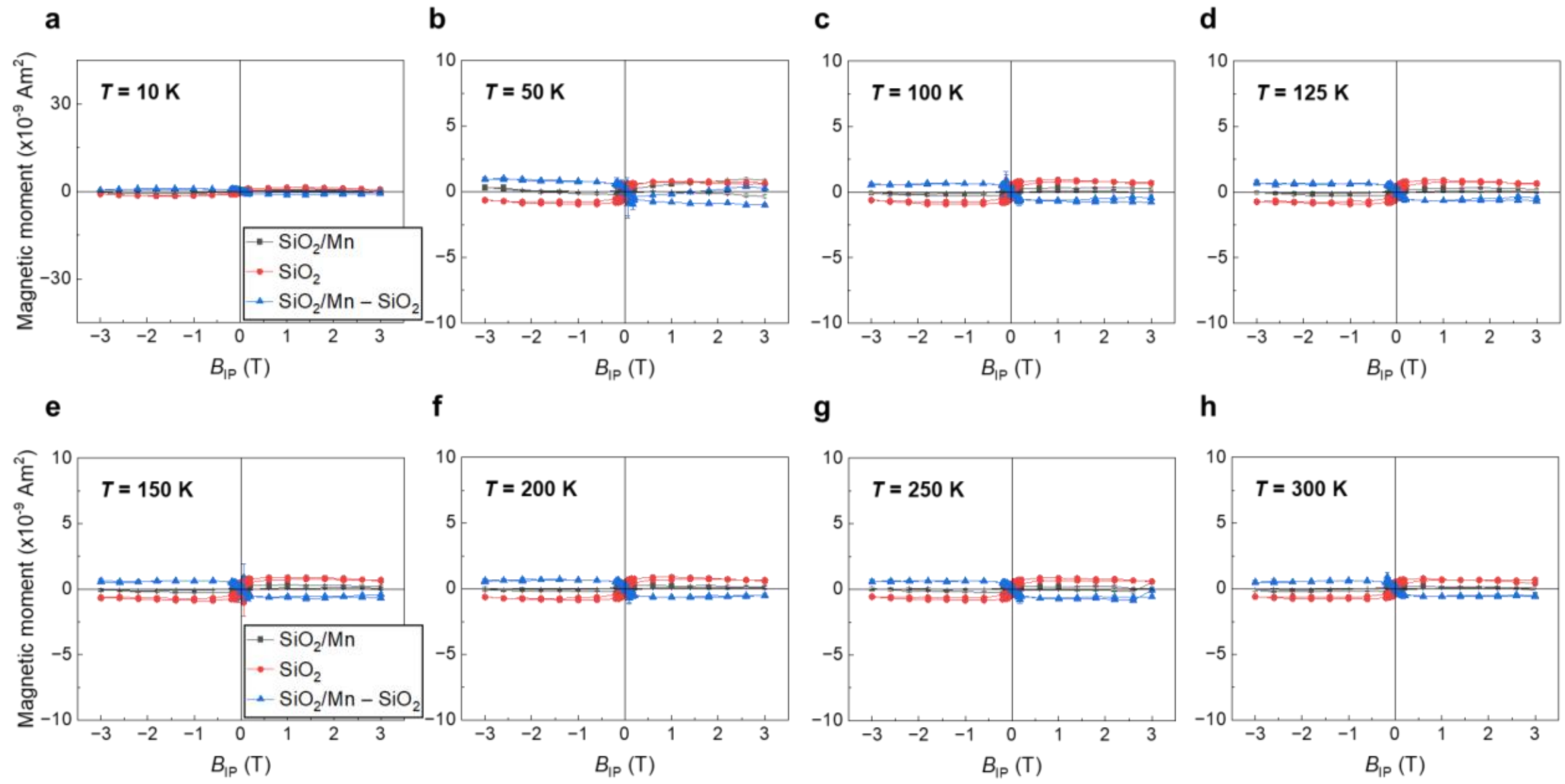

Figure S7 | Magnetic characterization of polycrystalline Mn using SQUID. a-h, Magnetization curves (magnetic moment vs. in-plane magnetic field B_{IP}) measured for 10-nm-thick polycrystalline Mn single layer (black curves), SiO_2 substrate reference (red curves), and their difference (blue curves). Different panels correspond to different temperatures. Error bars of the Mn signal correspond to propagated uncertainties from SiO_2/Mn and SiO_2 measurements.

Supplementary Note 3. Magnetoresistance at low temperature

Figure S8 shows the longitudinal and transverse magnetoresistance curves of epitaxial α-Mn(001) measured at 100 K and 50 K. The distinct behaviours observed at different temperatures reflect transitions in the dominant transport mechanisms, ranging from orbital Hall effect in the paramagnetic phase above the Néel temperature T_N to antiferromagnetic ordering and multi-band transports effects at lower temperatures.

As discussed in the main text, at 300 K, above T_N, the magnetoresistance is dominated by the orbital Hall effect due to the momentum-dependent orbital texture in the centrosymmetric bcc structure of α-Mn. At low temperature below T_N, the orbital HMR signals are suppressed, and AFM ordering dominates the transport properties. Negative AMR emerges, reflecting the interaction between the Néel vector, the charge current, and the applied magnetic field. In particular, since the Néel vector aligns perpendicular to the applied magnetic field, when the magnetic field is applied parallel to the charge current ($\boldsymbol{B} \parallel \boldsymbol{x}$), electronic scattering is minimized leading to lower resistivity. On the contrary, a magnetic field perpendicular to the charge current ($\boldsymbol{B} \parallel \boldsymbol{y}$ or $\boldsymbol{z}$) enhances electronic scattering and increases resistivity [18,19]. The hysteretic behaviour of the longitudinal magnetoresistance at low fields can be attributed to the presence of complex AFM domains with randomly oriented Néel vectors and associated domain wall scattering [20,21]. We note that the absence of hysteretic behaviour in the negative magnetoresistance of the more disordered films (textured and polycrystalline) rules out the possibility of ferromagnetic impurity-induced hysteresis, as all films evaporated from the same Mn source. In addition to this directional dependence, the AMR response in antiferromagnets is linked to the symmetry of the electronic band structure and spin-dependent scattering [22]. The magnetic phase transition of α-Mn at T_N is accompanied by a structural transition from the paramagnetic bcc structure to the AFM tetragonal structure [23]. The AFM spin arrangement induces anisotropic electronic bands [23], which contribute to the observed transport properties [24–26], particularly in the transverse magnetic field configuration, resulting in a nonzero Hall-like response as a function of B_z. Note that two scenarios can in principle explain the disappearance of the orbital HMR at T_N: (i) AFM–related transport features emerging below T_N can obscure the orbital HMR, and (ii) the orbital HMR vanishes entirely at T_N due to enhanced orbital relaxation. These two effects may also coexist. The appearance of a finite longitudinal magnetoresistance for $\boldsymbol{B} \parallel \boldsymbol{y}$ and the simultaneous decrease of the transverse MR when $\boldsymbol{B} \parallel z$, however, tends to exclude scenario (i). This leaves (ii) as the more plausible scenario.

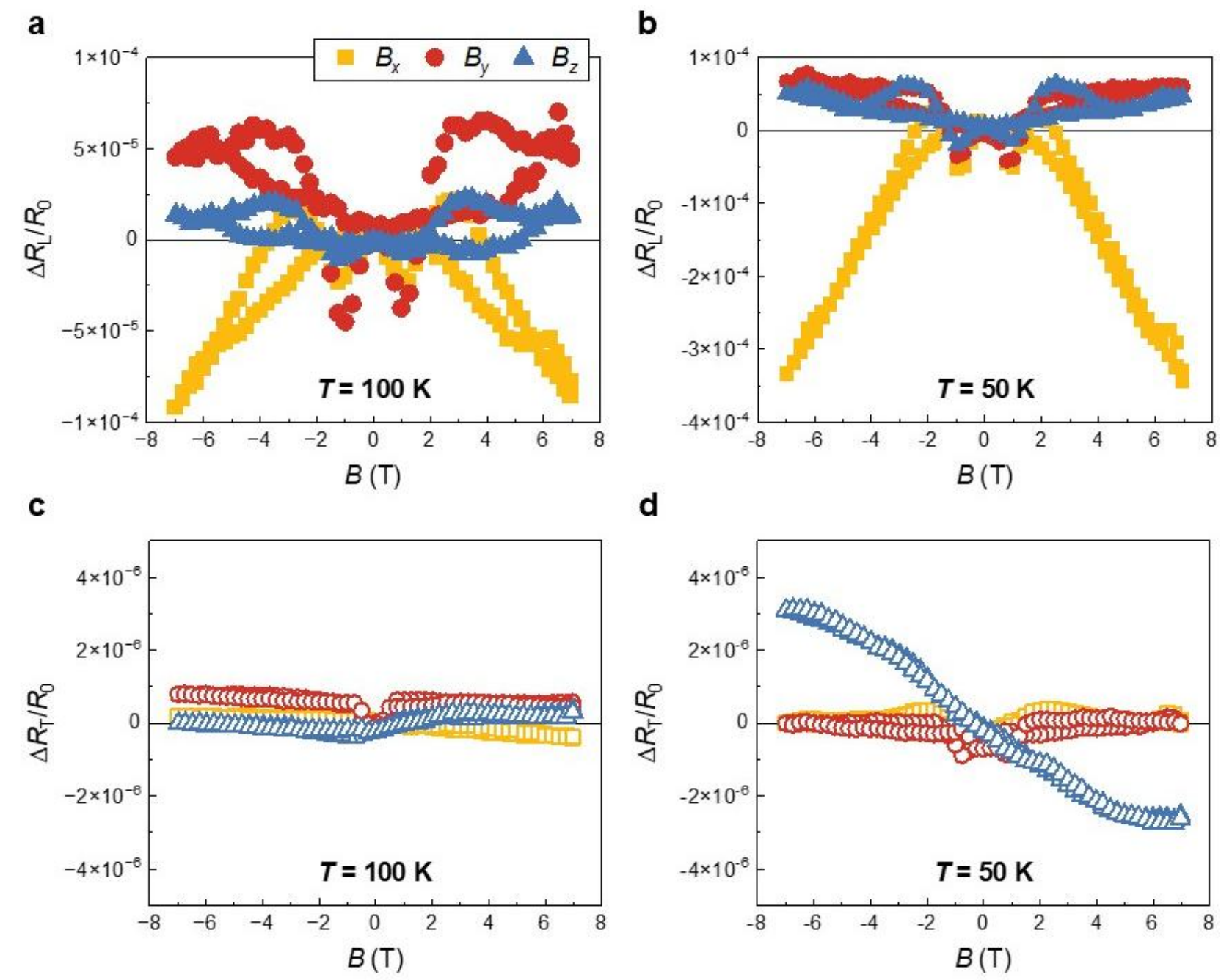

Figure S8 | Field dependence of magnetoresistance in epitaxial Mn(001). **a,b**, Longitudinal ($\Delta R_L/R_0$) and **c,d**, transverse ($\Delta R_T/R_0$) magnetoresistances measured under magnetic fields oriented along the *x*, *y*, and *z* axes at 100 K (a,c), and 50 K (b,d).

Supplementary Note 4. Additional HMR measurements

Expanded dataset for orbital relaxation scaling

To verify the orbital relaxation scaling in the good metal regime (defined by the positive TCR at room temperature), we performed two independent sets of additional experiments, all focusing on epitaxial α-Mn films:

(i) Thickness dependence of epitaxial α-Mn (**Fig. S9**): We grew an additional epitaxial α-Mn film with a thickness of 20 nm, in comparison to the original 10-nm-thick sample. XRD measurements (**Fig. S9a**) confirm the same α-Mn(001) phase for both films. Using the orbital Hanle fitting procedure described in the main text, we extract the orbital relaxation time at room temperature. Upon increasing the conductivity σ from 5.22×10^3 to $6.09 \times 10^3\ \Omega^{-1}\mathrm{cm}^{-1}$, the orbital relaxation time τ_L increases from 0.98 ± 0.04 ps to 1.32 ± 0.10 ps. This clear increase of τ_L with conductivity within the same crystal phase directly supports EY-like behavior.

(ii) Substrate-engineered conductivity of epitaxial α-Mn **(Fig. S10):** We next investigated the robustness of the EY-like scaling within epitaxial α-Mn by tuning the conductivity through controlled modification of the MgO(001) substrate's surface. **Figure S10** compares three epitaxial α-Mn films, Mn_{LowT}, Mn_{RT1}, and Mn_{RT2}, all with the same thickness and crystal phase, but grown under different interfacial conditions. Mn_{RT2} was prepared by depositing Mn at room temperature after pre-annealing the MgO(001) substrate in a tube furnace under an oxygen atmosphere, followed by degassing of the substrate in UHV prior to Mn growth. In contrast, no tube-furnace treatment was performed for Mn_{RT1} and Mn_{LowT}. Mn_{LowT} was grown at lower substrate temperature as indicated in the main text (see **Methods**). XRD measurements confirm that all three films remain in the epitaxial α-Mn phase (**Fig. S10a**). This controlled variation of growth and interface conditions leads to a systematic increase in conductivity from 5.22×10^3 to $8.03 \times 10^3\ \Omega^{-1}\mathrm{cm}^{-1}$ from Mn_{LowT} to Mn_{RT1}, and Mn_{RT2} (see Table S1). Importantly, the extracted orbital relaxation time τ_L increases monotonically from 0.98 to 1.37 ps across these samples. This trend reproduces the EY-like scaling observed in Fig. 3(b) of the main text within a single crystal phase, demonstrating that the scaling does not rely on changing structural classes.

When all metallic-regime data points, including the additional epitaxial samples introduced above, are combined, the scaling of orbital relaxation time yields $\gamma = 0.51 \pm 0.07$, as shown in **Fig. S11** and discussed in the main text.

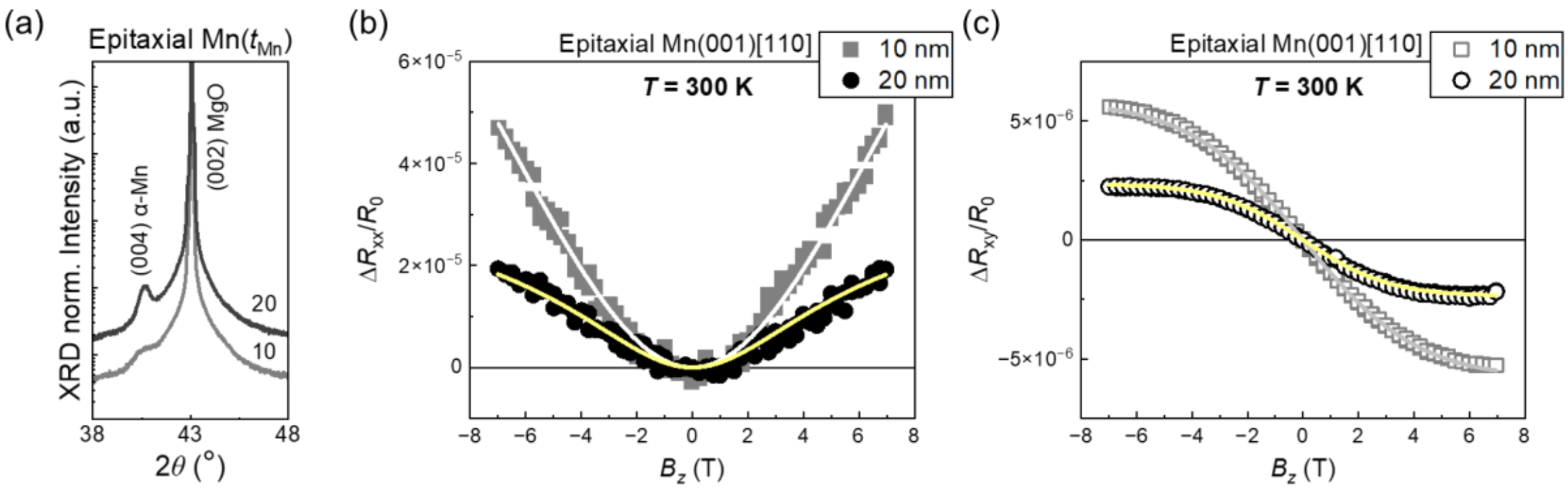

Figure S9 | Thickness dependence of orbital relaxation in epitaxial α-Mn. (a) XRD *θ/2θ* scans of single-crystal epitaxial α-Mn films with thicknesses of 10 nm and 20 nm grown on MgO(001), confirming the α-Mn(001) phase. **(b)** Longitudinal and **(c)** transverse Hanle magnetoresistance measured at room temperature for the two thicknesses. Solid lines are fits to the orbital Hanle model used in the main text. The extracted transport parameters are summarized in Table R1.

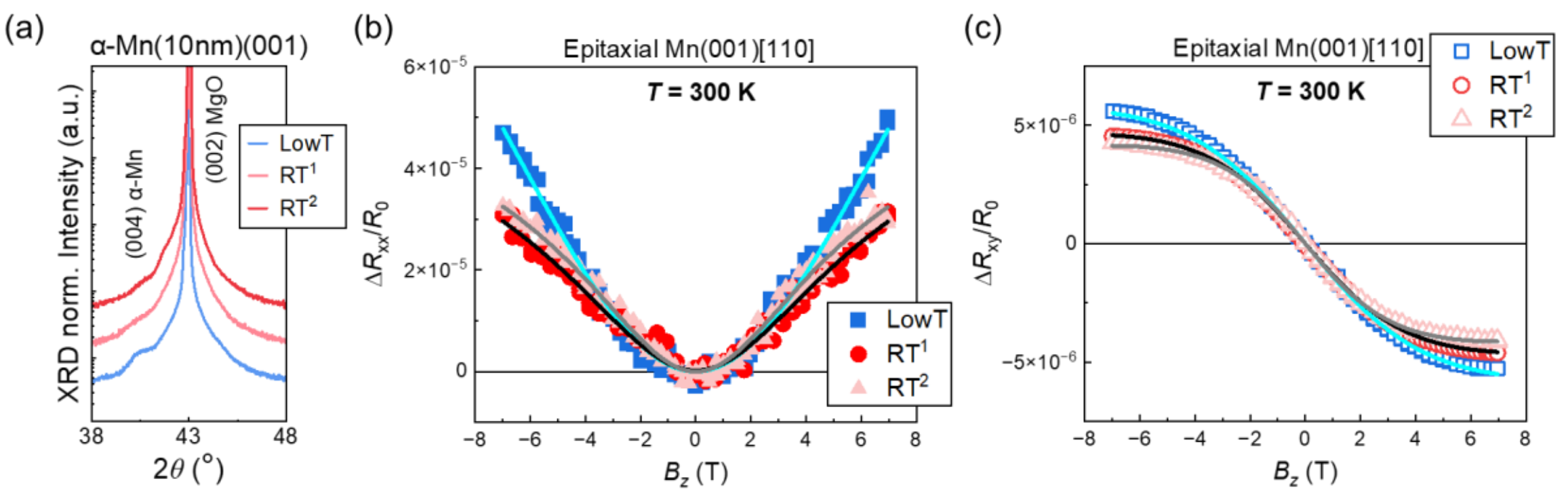

Figure S10 | Growth-temperature dependence of orbital relaxation in epitaxial α-Mn. (a) XRD *θ/2θ* scans of 10 nm epitaxial α-Mn films grown at different substrate temperatures, confirming the presence of α-Mn phase for all the films. **(b)** Longitudinal and **(c)** transverse Hanle magnetoresistance measured at room temperature. Solid lines represent fits to the orbital Hanle model. Extracted fitting parameters are listed in Table R1.

Samples	σ $[10^3 \Omega^{-1} cm^{-1}]$	σ_L $[\hbar/e\ \Omega^{-1}\ cm^{-1}]$	τ [ps]	θ
Epi-Mn_{LowT} (10 nm)	5.22	52 ± 10	0.98 ± 0.04	0.019 ± 0.001
Epi-Mn_{LowT} (20 nm)	6.09	26 ± 12	1.32 ± 0.13	0.008 ± 0.005
Epi-Mn_{RT1} (10 nm)	5.95	31 ± 12	1.18 ± 0.02	0.010 ± 0.002
Epi-Mn_{RT2} (10 nm)	8.03	62 ± 11	1.37 ± 0.02	0.015 ± 0.005

Table S1 | Fitting parameters extracted from Hanle analysis. Extracted electrical conductivity (σ), orbital Hall conductivity (σ_L), orbital Hall angle (θ_{OH}), and orbital relaxation time (τ_L) extracted from Hanle fitting for epitaxial Mn films.

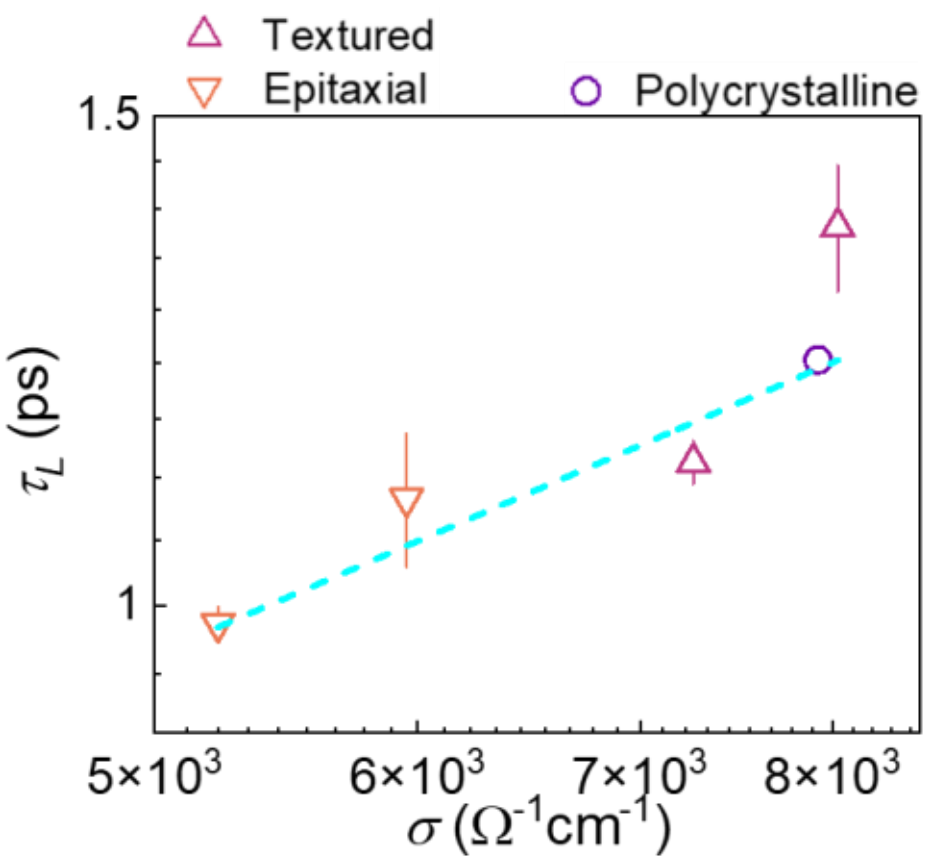

Figure S11 | Scaling of orbital relaxation time in metallic Mn at room temperature. Orbital relaxation time τ_L as a function of longitudinal conductivity σ for epitaxial, textured, and high-temperature-grown polycrystalline Mn, including all additional datasets shown in Figs. S9–S10. The solid line is a power-law fit $\tau_L \propto \sigma^{\gamma}$, yielding $\gamma \approx 0.5$. Error bars reflect fitting uncertainties.

Discussion of possible interfacial contributions to the observed HMR

We note that an orbital Rashba–Edelstein effect (OREE) [30] at the Mn surface or at the Mn/substrate interface cannot be completely ruled out in principle. However, several experimental observations consistently indicate that such interfacial contributions are unlikely to be the dominant mechanism in our system.

First, we observe that the HMR signal is essentially independent of both the substrate and the capping layer. Specifically, comparable HMR responses are obtained for Mn films grown on MgO and on Si_3N_4 substrates, and for devices capped with either AlO_x or SiN. If the signal were primarily governed by an interfacial OREE (or even a spin REE) a strong dependence on the specific interface (substrate or capping material) would be expected. The absence of such dependence suggests that the dominant contribution does not originate from a particular interface.

Second, we exploit thickness-dependent measurements to test an interfacial scenario. For sputtered amorphous Mn films grown under different Ar pressures (**Fig. S12**), we observe a nonmonotonic dependence of the HMR amplitude on film thickness, with a pronounced peak at a thickness between 5 and 10 nm. Such behavior is incompatible with a simple interfacial Rashba mechanism, for which one would generally expect a monotonic decrease of the signal with increasing film thickness due to bulk shunting effects. Moreover, both the position of the peak and the overall thickness dependence shift systematically with the sputtering pressure, indicating a strong sensitivity to the film microstructure.

We further investigated epitaxial Mn films with thicknesses of 10 nm and 20 nm (**Fig. S9**). In both cases, clear and reproducible Hanle signatures are observed in the transverse magnetoresistance measured under out-of-plane magnetic fields, indicating robust orbital accumulation and precession in the films. Quantitative analysis using the same fitting procedure as in the main text yields orbital relaxation times of τ_L = 0.98 ps for the 10 nm film and τ_L = 1.32 ps for the 20 nm film, with all fitting parameters summarized in Table S1. Importantly,

increasing the film thickness leads to a simultaneous increase in both the longitudinal conductivity and the orbital relaxation time. This trend follows the same Elliott–Yafet (EY)–like scaling between τ_L and conductivity discussed in Fig. 3(b) of the main text and is reproduced here within a single crystallographic phase by varying only the film thickness. The fact that τ_L increases systematically with conductivity, while the interfacial environment remains unchanged, strongly indicates that orbital relaxation is governed by bulk momentum-scattering processes rather than by interface-specific effects. If the observed relaxation were dominated by an interfacial Rashba mechanism, one would instead expect τ_L to be largely insensitive to film thickness once the thickness exceeds the interfacial region. The observed thickness-dependent enhancement of τ_L therefore provides additional evidence that the dominant relaxation mechanism in epitaxial Mn is bulk-controlled, consistent with the EY–like interpretation presented in the main text.

Taken together, the weak dependence on substrate and capping layers, the negligible expected spin contribution, and the nonmonotonic thickness dependence of the HMR all suggest that the bulk orbital Hall effect provides a more consistent interpretation of our results. While a minor interfacial contribution cannot be completely excluded, our data indicate that it does not play a dominant role in the observed magnetoresistance.

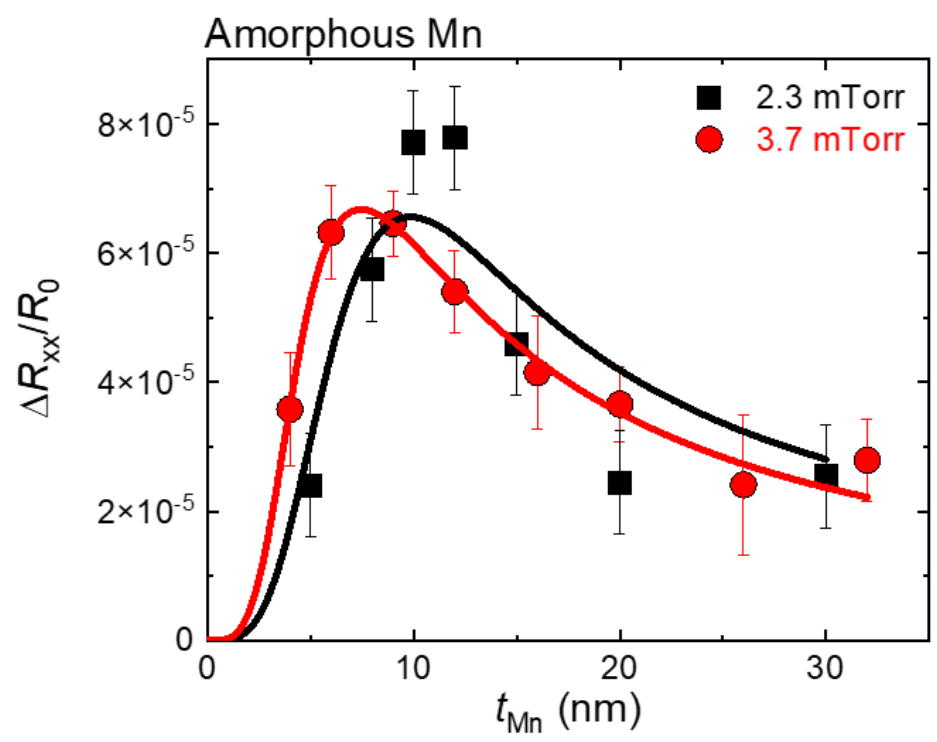

Figure S12 | Thickness-dependent Hanle magnetoresistance ($\Delta R_{xx}/R_0$) in amorphous Mn films deposited with different Ar pressure. Error bars represent uncertainties from nonlinear least-squares fits.

Supplementary References

[1] Lawson, A. C., Larson, A. C., Aronson, M. C., Johnson, S., Fisk, Z., Canfield, P. C., Thompson, J. D. & Von Dreele, R. B. Magnetic and crystallographic order in α-manganese. *J. Appl. Phys.* **76**, 7049–7051 (1994).

[2] Materials Project. Data on Mn (2020). https://doi.org/10.17188/1309841.

[3] Jing, C., Cao, S. X. & Zhang, J. C. Lattice constant dependence of magnetic properties in bcc and fcc Fe_xMn_{1-x} alloys. *Phys. Rev. B* **68**, 224407 (2003).

[4] Tian, D., Wu, S. C., Jona, F. & Marcus, P. M. Epitaxy of Mn on Pd (001). *Solid State Commun.* **70**, 199–204 (1989).

[5] Dhananjayan, N. & Banerjee, T. *Structure of Electro-Deposited Manganese*, Ch. 1 (CSIR-NML, 1969).

[6] Bacon, G. E. & Cowlam, N. A study of some alloys of γ-manganese by neutron diffraction. *J. Phys. C: Solid State Phys.* **3**, 675 (1970).

[7] Bacon, G. E., Dunmur, I. W., Smith, J. H., Street, R. & Sucksmith, S. The antiferromagnetism of manganese-copper alloys. *Proc. R. Soc. Lond. A* **241**, 223–238 (1957).

[8] Shiomi, Y. Magnetotransport properties of γ-FeMn thin films grown by high-temperature sputtering. *AIP Adv.* **8**, 085018 (2018).

[9] Hori, T., Tsuchiya, Y., Ishii, Y. & Hojou, K. Crystal distortion and magnetic structure of γ-MnPd alloys. *Mater. Trans.* **43**, 436–438 (2002).

[10] Nosova, G. I. FCC → FCT martensitic transformation in two-phase Mn–Cu alloys. *Russ. Metall.* **2017**, 216–220 (2017).

[11] Duschanek, H., Mohn, P. & Schwarz, K. Antiferromagnetic and ferromagnetic γ-manganese: generalisation of the fixed-spin-moment method. *Phys. B Condens. Matter* **161**, 139–142 (1989).

[12] Dalla Longa, F., Kohlhepp, J. T., de Jonge, W. J. M. & Koopmans, B. Laser-induced ultrafast spin dynamics in epitaxial Co/Mn exchange-coupled bilayers. *J. Appl. Phys.* **99**, 08F304 (2006).

[13] Lin, W. C., Lin, L. C., Chen, T. Y., Wang, B. Y., Song, K. J. & Lin, M. T. Growth, structure and magnetism of γ-phase Mn ultrathin films on Cu_3Au(100). *J. Appl. Phys.* **97**, 10K112 (2005).

[14] Schirmer, B., Feldmann, B., Sokoll, A., Gauthier, Y. & Wuttig, M. Tetragonal distortion of Mn films on Cu_3Au(100). *Phys. Rev. B* **60**, 5895–5903 (1999).

[15] Jin, X., Zhang, M., Dong, G. S., Xu, M., Chen, Y., Wang, Xun, Zhu, X. G. & Shen, X. L. Stabilization of face-centred-cubic Mn films via epitaxial growth on GaAs(001). *Appl. Phys. Lett.* **65**, 3078–3080 (1994).

[16] Moiseev, V. P. & Popova, O. S. X-ray structure study of electrolytic manganese deposits. *Russ. J. Phys. Chem.* **33**, 344 (1959).

[17] Endoh, Y. & Ishikawa, Y. Antiferromagnetism of γ-iron manganese alloys. *J. Phys. Soc. Jpn.* **30**, 1614–1627 (1971).

[18] Jungwirth, T., Marti, X., Wadley, P. & Wunderlich, J. Antiferromagnetic spintronics. *Nat. Nanotechnol.* **11**, 231 (2016).

[19] Baltz, V., Manchon, A., Tsoi, M., Moriyama, T., Ono, T. & Tserkovnyak, Y. Antiferromagnetic spintronics. *Rev. Mod. Phys.* **90**, 015005 (2018).

[20] Kriegner, D., Výborný, K., Olejník, K., Reichlová, H., Novák, V., Marti, X., Gazquez, J., Saidl, V., Němec, P., Volobuev, V. V., Springholz, G., Holeš, V. & Jungwirth, T. Multiple-stable anisotropic magnetoresistance memory in antiferromagnetic MnTe. *Nat. Commun.* **7**, 11623 (2016).

[21] Wang, H., Lu, C., Chen, J., Liu, Y., Yuan, S. L., Cheong, S.-W., Dong, S. & Liu, J.-M. Giant anisotropic magnetoresistance and nonvolatile memory in canted antiferromagnet Sr_2IrO_4. *Nat. Commun.* **10**, 2280 (2019).

[22] Martí, X., Fina, I., Frontera, C., Liu, J., Wadley, P., He, Q., Paull, R. J., Clarkson, J. D., Kudrnovský, J., Turek, I., Kuneš, J., Yi, D., Chu, J. H., Nelson, C. T., You, L., Arenholz, E., Salahuddin, S., Fontcuberta, J., Jungwirth, T. & Ramesh, R. Room-temperature antiferromagnetic memory resistor. *Nat. Mater.* **13**, 367 (2014).

[23] Hobbs, D., Hafner, J. & Spišák, D. Understanding the complex metallic element Mn. I. Crystalline and noncollinear magnetic structure of α-Mn. *Phys. Rev. B* **68**, 014407 (2003).

[24] Boakye, F., Adam, K. G. & Grassie, A. D. C. Effect of substrate temperature on the Hall coefficient of α-Mn thin films. *Mater. Sci. Eng. B* **45**, 50 (1997).

[25] Foner, S. Hall effect in titanium, vanadium, chromium and manganese. *Phys. Rev.* **107**, 1513 (1957).

[26] McAlister, S. P., Lupton, L. R. & Hurd, C. M. Hall and magnetoresistance effects in mictomagnetic AuMn. *Solid State Commun.* **25**, 11 (1978).

[27] Sailler, S., Sala, G., Reustlen, D., Schlitz, R., Kang, M-G., Gambardella, P., Goennenwein, S. T. B. & Lammel, M. Competing ordinary and Hanle magnetoresistance in Pt and Ti thin films. *Phys. Rev. B* **111**, 104421 (2025).
[28] Salemi, L. & Oppeneer, P. M. First-principles theory of intrinsic spin and orbital Hall and Nernst effects in metallic monoatomic crystals. *Phys. Rev. Mater.* **6**, 095001 (2022).
[29] Go, D., Lee, H. W., Oppeneer, P. M., Blügel, S. & Mokrousov, Y. First-principles calculation of orbital Hall effect by Wannier interpolation: role of orbital dependence of the anomalous position. *Phys. Rev. B* **109**, 174435 (2024).
[30] Salemi, L., Berritta, M., Nandy, A. K. & Oppeneer, P. M. Orbitally dominated Rashba-Edelstein effect in noncentrosymmetric antiferromagnets. *Nat. Commun.* **10**, 5381 (2019).
