# Supplementary material for: Orbital Hall conductivity and relaxation in thin films with variable disorder
Source: Nat Phys. 2026 Jun 15;22(8):1238–44. doi: 10.1038/s41567-026-03334-z (PMC13731138; doi:10.1038/s41567-026-03334-z)
Supplement: Supplementary file 2 [file 41567_2026_3334_MOESM2_ESM.pdf]

nature portfolio

Peer Review File

Orbital Hall conductivity and relaxation in thin films with variable disorder

Corresponding Author: Professor Min-Gu Kang

Version 0:

Reviewer comments:

Reviewer #1

(Remarks to the Author)
The generation, control and transport of orbital currents have gained considerable attention owing to its prospect for the future development of orbitronics devices. Previous experimental and theoretical results have clarified orbital torques, current-induced magnetization switching in non-magnet/magnet heterostructures, orbital magnetoresistive effects and orbital angular momentum pumping and dissipation from a magnet into a non-magnetic layer etc. This paper deals with the generation, propagation and relaxation of orbital angular momentum with varying degrees of disorder, from strong disorder to the good metal limit. The findings of this paper are timely and important and can be considered for publication in Nature Physics, provided the authors have satisfactorily addressed the questions below:
(1) Page 3, line 91: “The degree of disorder in amorphous samples was varied by changing the Ar sputtering pressure, with higher pressure leading to increased resistivity and surface roughness, indicative of enhanced atomic-scale disorder”. Atomic force microscopy will only give an idea of the surface quality and not defects/dislocations or other disorders. It is not clear what does the authors mean when they refer to “degree of disorder”. It seems only the static disorder related to surface quality is considered here and quantified by AFM.
(2) The magnetization data shows paramagnetic nature of Mn at room temperature. It is not clear how the error bars in the magnetization data for Mn (obtained by subtracting magnetization of MgO/Mn to bare MgO) were calculated.
(3) Since the devices used for measurements appear to be uncapped, it is necessary to clarify any role of surface oxidation towards the Hanle magnetoresistive behavior seen in the Hall bar samples.
(4) Page 4, line 132: “Crystalline and textured samples display broader Hanle curves, consistent with fast orbital dephasing. In contrast, polycrystalline and amorphous samples exhibit sharper Hanle-like dips, indicative of longer orbital relaxation times. These measurements suggest that orbital dephasing scales with the degree of structural order.” While this is an observation from the experiments, a qualitative or quantitative theoretical description as to why this should occur needs to be justified.
(5) Figure 3 (a): Since the orbital Hall conductivity is obtained from the fit of the experimental data, error bars can be included for clarity.
(6) It is not clear whether the polycrystalline Mn samples do show any emergence of antiferromagnetic order. Extended Data Figure 1 shows resistivity minima in polycrystalline Mn at ~ 225K. Considering the resistivity minima to be a signature of onset of antiferromagnetic order (as stated by the authors), no corresponding sharp drop is seen temperature dependence of orbital Hall conductivity.
(7) Cross-section transmission electron microscopy showing the crystalline nature of Mn and comparison of the crystal structure with the data from X-ray and RHEED might help to substantially improve the quality of the manuscript.

Reviewer #2

(Remarks to the Author)
Overall comments.

Overall, this manuscript provides a careful and systematic experimental study of orbital transport in disordered metallic systems. By controlling disorder, the authors extract both the orbital Hall conductivity (OHC) and the orbital relaxation time with good precision, extending the investigation into the bad-metal regime that has been largely unexplored in prior work. From this dataset they report (i) an approximately universal scaling in which the OHC increases linearly with longitudinal conductivity, (ii) a counter-intuitive bad-metal regime where stronger disorder is accompanied by a longer orbital relaxation time, and (iii) unravel new orbital relaxation channel which is related to phonon dynamics. These are valuable empirical observations that help map out orbital transport across a wide disorder range.
That said, the overall impact is limited for several reasons. First, the claimed universal scaling is not convincingly established: its generality across temperatures and structural regimes remains unclear. Second, the main qualitative trend

that orbital transport can improve as sample quality degrades has been reported in earlier study [Nat. Mat., 24, 1749-1755 (2025)], so the central message here does not appear conceptually new. Third, the phonon-based interpretation of orbital generation and relaxation is novel and interesting; however, the manuscript lacks a sufficiently developed theoretical explanation that would convincingly support the experimental observations.
For these reasons, I find the experimental results promising and potentially impactful, but the manuscript does not seem to offer sufficient conceptual novelty to meet the expectations for a Nature Physics.

Questions to authors.

[1] On the linear scaling of OHC with conductivity.
The authors argue that, upon moving from epitaxial to amorphous samples, both the orbital generation and relaxation mechanisms change qualitatively (e.g., from an intrinsic orbital Hall effect (OHE) picture to phonon-activated hopping–induced orbital generation). Given that the crystal structures are also substantially different across these regimes, it is not obvious why the OHC would still follow the same linear scaling with conductivity.
For example, comparing Fig. 4(b) and Fig. 4(f), the former is interpreted as phonon-mediated hopping–induced OHE, whereas the latter is attributed to intrinsic OHE. The conductivity ranges are relatively close (5.97- 6.0 * 10^3 ohm^-1 cm^-1) vs. (5.22- 5.30 * 10^3 ohm^-1 cm^-1), yet the OHC behaves very differently: in Fig. 4(b) it varies strongly (13- 40 hbar/e ohm^-1 cm^-1), while in Fig. 4(f) it remains nearly constant (40- 50 hbar/e ohm^-1 cm^-1). This seems to suggest that the OHC– conductivity linearity may hold at some temperatures or in some regimes, but not universally.
Therefore, I have a question:
Could the authors clarify the scope of their claim? Specifically, do they argue that a linear OHC–conductivity scaling is universal even when the lattice structure and the orbital generation mechanisms differ substantially, or do they mean that such linear scaling emerges only within the phonon-mediated hopping regime? If the former is intended, I believe stronger evidence would be necessary to substantiate this universality—for instance, by demonstrating that the scaling is temperature-independent across the full range studied (rather than holding only at room temperature or in a limited regime), by showing that a similar linear trend persists in Mn films with thicknesses different from those used here, or by providing a clear microscopic theoretical justification.

[2] Extrinsic OHE.
For the extrinsic orbital Hall effect observed in the amorphous regime, can the authors clarify which mechanism they attribute it to (phonon-mediated skew scattering vs side jump)?

[3] Microscopic mechanism of phonon-induced orbital relaxation.
While the phonon-activated relaxation interpretation is interesting, it currently reads as speculative. Please provide a clearer theoretical model or independent evidence supporting this claim.

[4] On the Elliott–Yafet (EY) mechanism.
In Fig. 3(b), the assignment of the epitaxial and textured samples to an EY-type relaxation appears to rely on only two data points, which seems insufficient to draw a firm conclusion. Could the authors provide stronger evidence to substantiate the EY interpretation in the epitaxial and textured samples?

Reviewer #3

(Remarks to the Author)
This manuscript presents a detailed and fundamental investigation of the orbital Hall conductance (OHC) in Mn compounds exhibiting different degrees of disorder and transport regimes, ranging from epitaxial metallic and textured films to polycrystalline and amorphous samples, all grown under various sputtering conditions. In this study, Mn is chosen as an ideal prototypical material owing to its very small spin Hall conductivity and its low equilibrium orbital angular momentum (singlet state).

Using orbital Hanle magnetoresistance measurements performed over a wide temperature range, the authors rigorously demonstrate: (i) the existence and quantitative determination of the OHC in Mn, leading to orbital current generation and orbital polarization at the sample edges; (ii) the dependence of the OHC on disorder; and (iii) the dependence of the orbital relaxation (or decoherence) time on both disorder and phonon scattering rates (temperature).

The main results of this work include the demonstration of an intrinsic OHC (i.e., conductivity-independent) in the epitaxial and textured Mn series, and a conductivity-dependent OHC in amorphous samples, where variable-range hopping transport dominates. In contrast, the orbital decoherence time exhibits a more complex behavior: it increases with hopping conductivity as disorder is varied, while it decreases when phonon scattering is enhanced at higher temperatures. This behavior points to a crossover in the dominant orbital decoherence mechanisms depending on the underlying scattering process.

In my view, the breadth of systems investigated and the depth of the experimental analysis are highly impressive, and the conclusions drawn are both robust and of significant value to the rapidly emerging field of orbitronics. As this field continues to grow, the results presented here will undoubtedly prove to be important and broadly useful to the scientific community.

I therefore conclude that this work merits rapid publication in Nature Physics, provided that the authors adequately address the comments and questions raised below.

1) One of my my main concern relates to the quality of the Mn top surface investigated in the Hanle transport measurements. The manuscript does not address the possibility of slight oxidation or surface contamination of the Mn Hall bar, which could significantly alter—or even compromise—the measured magnetoresistance properties. This issue is neither discussed in the main text nor in the Supplementary Information.
Could the authors please comment on this important point and clarify how surface oxidation or contamination is controlled or ruled out?

2) How would the results and interpretation of the Hanle magnetoresistance be affected if orbital (or even spin) Rashba effects were present at the Mn top surface or at the bottom interface with the MgO substrate?
In particular, how do the authors exclude a possible orbital Rashba contribution, which could also give rise to magnetoresistance signals similar to those reported here (see, for example, Ref. [19])?

3) The manuscript does not describe the experimental protocol used to acquire the temperature dependence of the Hanle magnetoresistance. As emphasized by the authors, the temperature-induced changes in conductivity are relatively modest, requiring careful experimental procedures.
Could the authors specify how the temperature sweeps were performed (e.g., heating versus cooling), and whether any hysteresis or thermal stabilization effects were checked?

4) The orbital decoherence time in Mn extracted in this study is compared to typical spin-flip times in other 3d transition metals such as Ni or Cu, which are stated to be larger than 10 ps (Note that the two cited references [48-49] are not relevant to the sentence). While this assumption may be valid for Cu—where 3s–4d electronic scattering is absent—it is much less applicable to Fe, Co, or Ni, for which spin-flip times are generally expected to lie in the picosecond range or below.

Indeed, recent THz time-domain spectroscopy (THz-TDS) measurements report typical spin-flip times of approximately 300 fs for Fe and Ni (G. Bierhance et al., arXiv:2506.22327), and below 500 fs for CoFeB (R. Rouzegar et al., Phys. Rev. B 106, 144427 (2022)). These values are reported to be largely insensitive to surface quality or interfacial conditions, irrespective of the adjacent metallic layer.

May the author can comment on this issue ?

Version 1:

Reviewer comments:

Reviewer #1

(Remarks to the Author)
The authors have satisfactorily answered all the concerns. Considering their detailed and accurate responses which justifies the importance and novelty of this work, I suggest publication of the manuscript in Nature Physics.

Reviewer #2

(Remarks to the Author)
The authors have addressed most of the concerns raised in the initial review and clarified the scope of their claims. In particular, they now demonstrate more clearly that the OHC–conductivity scaling emerges in the amorphous, hopping-dominated regime. While the experimental analysis is careful and the dataset is valuable, and although further theoretical development would still be desirable, the central claim is now more precisely formulated.

However, it remains unclear whether the level of conceptual novelty meets the standards expected for Nature Physics. Closely related scaling behavior has already been established for the spin Hall effect across metallic and disordered regimes [Commun. Phys. 5, 12 (2022)]. In this context, the present results appear largely as an orbital counterpart of an already known phenomenology, without yet demonstrating qualitatively new physics beyond the spin case. The observation of a non-monotonic orbital relaxation time is interesting, but it is not yet clear whether this reflects a genuinely orbital-specific effect. As a side point, given that orbital transport is known to be sensitive to parameters such as film thickness, temperature, and interfacial conditions, it would be important to establish whether the proposed scaling remains robust across different amorphous samples. For example, it would be useful to clarify whether a similar scaling behavior persists for amorphous Mn films with different thicknesses.

Reviewer #3

(Remarks to the Author)
The authors have made all the necessary modifications and improvements in this revised version for the paper to be published in Nature Communications journal.

Open Access This Peer Review File is licensed under a Creative Commons Attribution 4.0 International License, which permits use, sharing, adaptation, distribution and reproduction in any medium or format, as long as you give appropriate credit to the original author(s) and the source, provide a link to the Creative Commons license, and indicate if changes were made.
In cases where reviewers are anonymous, credit should be given to 'Anonymous Referee' and the source.
The images or other third party material in this Peer Review File are included in the article's Creative Commons license, unless indicated otherwise in a credit line to the material. If material is not included in the article's Creative Commons license and your intended use is not permitted by statutory regulation or exceeds the permitted use, you will need to obtain permission directly from the copyright holder.
To view a copy of this license, visit https://creativecommons.org/licenses/by/4.0/

Reviewer comments:

Reviewer #1 (Remarks to the Author):

The generation, control and transport of orbital currents have gained considerable attention owing to its prospect for the future development of orbitronics devices. Previous experimental and theoretical results have clarified orbital torques, current-induced magnetization switching in non-magnet/magnet heterostructures, orbital magnetoresistive effects and orbital angular momentum pumping and dissipation from a magnet into a non-magnetic layer etc. This paper deals with the generation, propagation and relaxation of orbital angular momentum with varying degrees of disorder, from strong disorder to the good metal limit. The findings of this paper are timely and important and can be considered for publication in Nature Physics, provided the authors have satisfactorily addressed the questions below:

Response) We thank the reviewer for the careful evaluation of our manuscript and for stating that our work is "*timely and important*" and "*can be considered for publication in Nature Physics*," provided that the raised points are satisfactorily addressed. We greatly appreciate the constructive nature of the comments and the opportunity to further improve the quality and clarity of our work.

We have considered the reviewer's comments and revised the manuscript accordingly, including additional analyses and clarifications where appropriate. We hope that our responses and the corresponding revisions adequately address the reviewer's questions and further strengthen the manuscript. Below, we provide detailed point-by-point responses to each comment.

(1) Page 3, line 91: "The degree of disorder in amorphous samples was varied by changing the Ar sputtering pressure, with higher pressure leading to increased resistivity and surface roughness, indicative of enhanced atomic-scale disorder". Atomic force microscopy will only give an idea of the surface quality and not defects/dislocations or other disorders. It is not clear what does the authors mean when they refer to "degree of disorder". It seems only the static disorder related to surface quality is considered here and quantified by AFM.

Response) We agree that AFM primarily provides information on surface morphology and does not directly quantify microscopic disorder in terms of crystallographic defects or dislocations. We therefore clarify that, in this work, the "degree of disorder" in sputtered amorphous Mn refers to the extent of growth-induced microstructural inhomogeneity that governs charge and orbital angular momentum transport. This disorder can be primarily assessed using transport metrics, while AFM served as a complementary surface-sensitive characterization.

In our sputtering process, the Ar pressure is a key growth parameter that controls the kinetic conditions of Mn atoms impinging on the substrate, thereby influencing film densification and nanostructure [*Acta

Materialia 183, 504-513 (2020); *Journal of Vacuum Science & Technology A* 23.4, 986-990 (2005)]. Because the films are amorphous, disorder cannot be described in terms of crystallographic defects, but rather in terms of microscopic features such as local variations in atomic density, film continuity, and characteristic structural length scales. These inhomogeneities affect charge scattering throughout the film and are directly reflected in the resistivity of the samples.

To experimentally demonstrate how sputtering pressure tunes this microstructural disorder, we added thickness-dependent transport measurements for amorphous Mn grown at 2.3 and 3.7 mTorr (**Fig. R1**). In **Fig. R1**, we observe a pressure-dependent critical thickness above which the resistivity saturates and becomes thickness independent. This behavior indicates a crossover from a thickness-limited transport regime to a regime in which charge transport is governed by microstructural disorder rather than film thickness. The existence of such a critical thickness suggests that the effective disorder correlation length relevant for charge transport, arising from spatial variations in local density or structural inhomogeneity, rather than from well-defined grains, is comparable to this thickness. Importantly, the dependence of this threshold thickness on sputtering pressure demonstrates that the disorder length scale is effectively tuned by the growth conditions.

In addition, to directly answer the referee's question, we performed cross-sectional high-angle annular dark-field scanning TEM (HAADF-STEM) measurements to examine the microstructure of amorphous films grown at different sputtering pressures of 4 and 40 mTorr (**Fig. R5c-d**). Both films lack long-range crystalline order, exhibiting only short- to medium-range nanoscale structural correlations. These correlations are typical of amorphous monatomic metal films due to the very fast nucleation and crystal growth kinetics of single-element metals. The film deposited at lower pressure shows a relatively uniform distribution of small correlated regions with typical size of about 1 nm, whereas the higher-pressure film exhibit a broader and more inhomogeneous distribution of structurally correlated regions with 1-10 nm size. This evolution is consistent with the pressure-dependent disorder inferred from transport measurements. These data therefore provide independent structural evidence that sputtering pressure systematically tunes the microstructural disorder beyond what can be captured by AFM alone.

We further corroborate this interpretation with temperature-dependent resistivity measurements (**Fig. R2**). **Figure R2(a)** shows the ρ(T) curve for the film grown at 2 mTorr. At high temperature, the resistivity decreases and the temperature coefficient of resistivity (TCR) approaches zero already around ~280 K, consistent with a crossover from a thermally-activated, hopping-dominated regime toward a more itinerant transport regime. In contrast, **Fig. R2(b)** shows the corresponding data for the film grown at 4 mTorr, where the resistivity remains higher and the TCR stays negative up to room temperature, indicating that hopping-dominated transport persists over the entire measured temperature range. This systematic difference demonstrates that sputtering pressure not only modifies the absolute resistivity but also controls the underlying transport regime. In addition, **Supplementary Fig. S3** in the original

Supplementary Note 1 explicitly shows the correlation between surface roughness and transport-based disorder metrics, further clarifying that disorder is not inferred from AFM alone.

Based on the considerations above, we revised the manuscript to clarify the definition of disorder. Specifically, on page 3, lines 91-97, we revised to "*Single crystal, textured, and polycrystalline films were grown by molecular beam epitaxy (MBE). Crystalline quality was controlled via the choice of substrate and growth temperature, enabling access to textured and polycrystalline phases with intermediate order (see Methods). Nanocrystalline-amorphous films were grown by magnetron sputtering, changing the Ar sputtering pressure to vary the degree of disorder. Increasing pressure results in higher resistivity and enhanced structural inhomogeneity characterized by the coexistence of nanocrystalline and amorphous phases, and increased surface roughness.*"

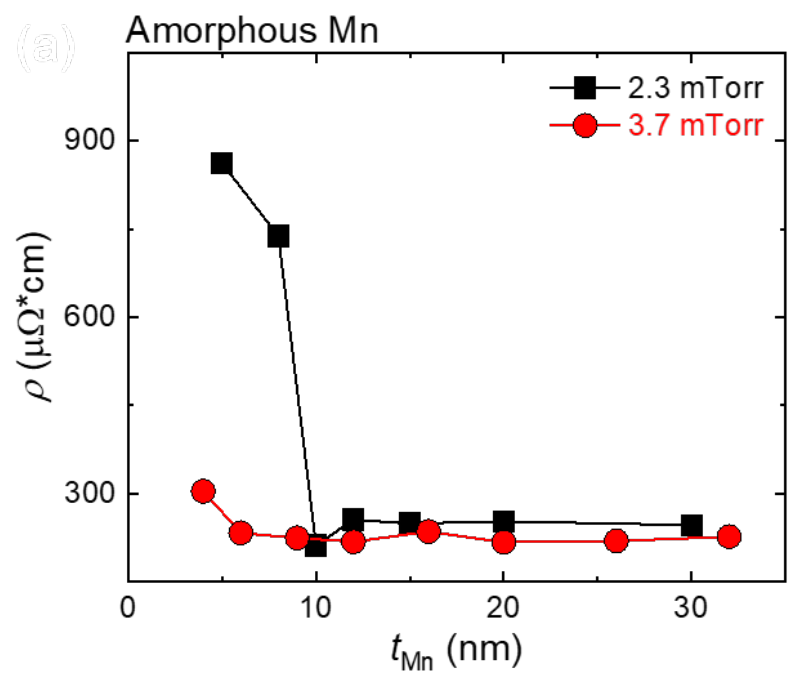

Figure R1 | Longitudinal resistivity ρ as a function of Mn thickness t_{Mn} for films grown at 2.3 and 3.7 mTorr.

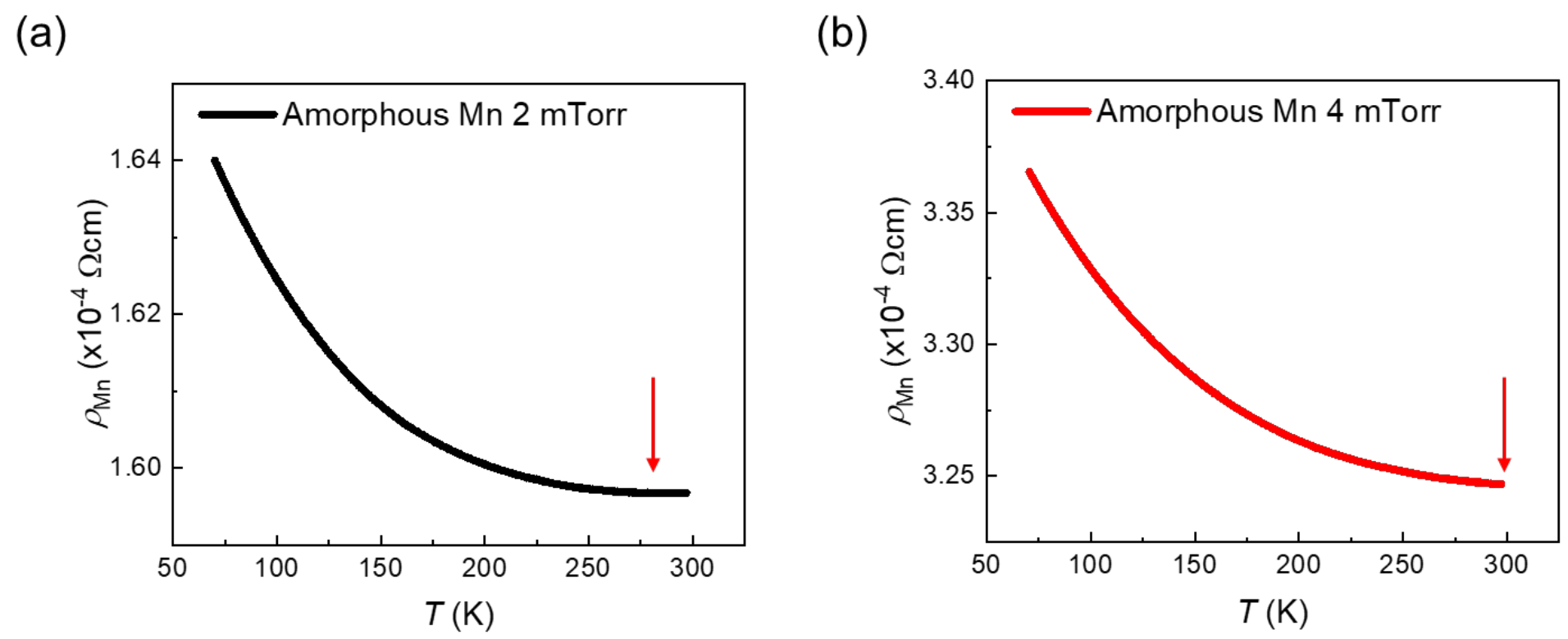

Figure R2 | Temperature-dependent resistivity of amorphous Mn films. **(a)** $\rho(T)$ for the film grown at 2 mTorr. **(b)** $\rho(T)$ for the film grown at 4 mTorr. The arrows indicate 280 K at 2mTorr and 300 K at 4mTorr.

(2) The magnetization data shows paramagnetic nature of Mn at room temperature. It is not clear how the error bars in the magnetization data for Mn (obtained by subtracting magnetization of MgO/Mn to bare MgO) were calculated.

Response) The reviewer correctly notes that the magnetization of the Mn layer was obtained by subtracting the signal of the bare MgO substrate from that of the MgO/Mn sample. The error bars shown in the Mn magnetization data were calculated using standard error propagation for subtraction.

Specifically, if Δx denotes the uncertainty of the MgO/Mn measurement and Δy denotes the uncertainty of the bare MgO reference, the resulting uncertainty of the difference Mn signal was obtained as $\Delta M = \sqrt{(\Delta x)^2 + (\Delta y)^2}$. This root-mean-square propagation accounts for the independent uncertainties of both measurements. We have now clarified this procedure in Supplementary Note 2 to avoid any ambiguity.

(3) Since the devices used for measurements appear to be uncapped, it is necessary to clarify any role of surface oxidation towards the Hanle magnetoresistive behavior seen in the Hall bar samples.

Response) We thank the reviewer for raising this important point, which allows us to clarify an essential experimental aspect of our study. We agree that uncontrolled surface oxidation could significantly affect magnetotransport measurements, and we therefore took particular care to prevent any exposure of Mn to ambient conditions throughout fabrication and measurement.

For the MBE-grown epitaxial Mn samples, an Al capping layer with a thickness of approximately 3 nm was deposited *in situ* immediately after Mn growth, without breaking vacuum. This Al layer was subsequently oxidized under ambient conditions to form a stable AlO_x protective layer. After device fabrication, we additionally performed O_2 plasma ashing to ensure complete oxidation of the Al layer. This procedure guarantees a fully insulating and chemically stable AlO_x cap. The presence of this capping layer is already visible in Fig. 1(c); however, the fabrication details were insufficiently described in the original manuscript.

For all sputtered Mn samples, an ~8 nm thick SiN capping layer was deposited immediately after Mn growth by RF sputtering at an Ar pressure of 10 mTorr. SiN is a well-established diffusion barrier and effectively prevents oxygen penetration. As a result, the Mn layer was never exposed to air at any stage of processing or measurement.

Therefore, we can confidently exclude contributions from surface oxidation to the observed Hanle magnetoresistance signals.

To avoid any ambiguity, we have now explicitly revised the Methods section and added the following sentences (page 10, lines 300-305): "*To prevent surface contamination of Mn, MBE-grown epitaxial films were protected by an Al capping layer (~3–4 nm) deposited in situ immediately after growth, which was subsequently oxidized under ambient conditions. After device fabrication, an additional O_2 treatment was applied to ensure complete oxidation of the Al layer, forming a stable AlO_x cap. For sputtered Mn films, an ~8 nm SiN capping layer was deposited by RF sputtering at an Ar pressure of 10 mTorr immediately after Mn growth.*"

(4) Page 4, line 132: "Crystalline and textured samples display broader Hanle curves, consistent with fast orbital dephasing. In contrast, polycrystalline and amorphous samples exhibit sharper Hanle-like dips, indicative of longer orbital relaxation times. These measurements suggest that orbital dephasing scales with the degree of structural order." While this is an observation from the experiments, a qualitative or quantitative theoretical description as to why this should occur needs to be justified.

Response) We thank the reviewer for this thoughtful comment. We agree that explicitly explaining the connection between the Hanle line shape and the underlying relaxation dynamics would improve the clarity of our presentation.

First, we would like to clarify that the difference in Hanle linewidths directly reflects the extracted orbital relaxation time τ_L through the standard Hanle expression $\Delta R(B) \propto 1/[1+(\omega_L \tau_L)^2]$, where ω_L is the Larmor frequency of the orbital angular momentum. Thus, broader Hanle curves quantitatively correspond to shorter relaxation times, while narrower curves reflect longer τ_L. Therefore, the evolution of the Hanle line shape observed in Fig. 2 is already quantitatively captured by our fitting procedure and by the extracted τ_L values shown in Fig. 3.

We note that this relation between Hanle line shape and relaxation dynamics has been well established in the literature. In particular, the connection between Hanle precession, diffusion, and relaxation has been explicitly discussed in the context of nonequilibrium angular momentum dynamics [M. I. Dyakonov, *Phys. Rev. Lett.* **99**, 126601 (2007)]. This work provides a solid conceptual framework supporting the use of Hanle linewidth as a quantitative probe of relaxation dynamics, which we adopt here.

Beyond this quantitative relation, the reviewer raises the important question of why τ_L itself depends on structural order. Indeed, the experimental demonstration of the relationship between τ_L and structural order is one of the main outcomes of our work. However, a microscopic theory for orbital relaxation in disordered metals is currently lacking. We hope that our results will stimulate theoretical work in this direction, particularly in strongly disordered systems with hopping-dominated conductivity, which have not yet been considered by either theory or experiments thus far. We can nonetheless provide a qualitative physical interpretation of this dependence. In crystalline and textured Mn films, electrons propagate in a well-defined periodic potential and experience a coherent local crystal field. In such an environment, the orbital angular momentum is most strongly coupled to the lattice, which naturally promotes faster orbital precession and dephasing. This scenario is consistent with the shorter τ_L and broader Hanle curves observed in these samples.

In contrast, in polycrystalline and amorphous films, the crystal field is spatially inhomogeneous. Moreover, electronic transport becomes increasingly hopping-like. In this regime, orbital dephasing can still occur due to the local crystal field at any given site. However, as electrons hop from site to site, the

crystal field changes and its effect average out. In other words, hopping events between locally disordered environments suppress coherent orbital precession and lead to slower orbital dephasing. This naturally results in longer orbital relaxation times and manifests experimentally as sharper Hanle features. In the revised manuscript, we made this physical interpretation more explicit.

To address the reviewer's concern, we added the following sentence at the end of the paragraph on page 5, lines 153-156: "*This trend can be qualitatively understood from the local crystal-field environment governing orbital dynamics: ordered crystalline films induce fast orbital precession and thus faster dephasing, whereas structural disorder and hopping tend to average out the crystal field, extending the orbital relaxation time.*"

(5) Figure 3 (a): Since the orbital Hall conductivity is obtained from the fit of the experimental data, error bars can be included for clarity.

Response) We thank the reviewer for this helpful suggestion. We agree that including information on the uncertainty of the extracted orbital Hall conductivity improves the clarity of the data presentation. The orbital Hall conductivity is defined as $\sigma_L = \sigma \times \theta_{OH}$, where σ is the longitudinal electrical conductivity obtained from direct transport measurements, and θ_{OH} is the orbital Hall angle extracted from the Hanle fitting. The uncertainty of σ_L was evaluated using standard error propagation. For a quantity defined as $z = xy$, the propagated error is given by $\Delta z = \sqrt{(y\Delta x)^2 + (x\Delta y)^2}$, assuming that the two variables are independent. Accordingly, we used $\Delta\sigma_L = \sqrt{(\theta_{OH}\Delta\sigma)^2 + (\sigma\Delta\theta_{OH})^2}$, where $\Delta\sigma$ is the uncertainty of the conductivity measurement and $\Delta\theta_{OH}$ is obtained from the fitting uncertainty of the Hanle analysis.

Following the reviewer's suggestion, we have now explicitly included these error bars in Figs. 3 and 4 of the revised manuscript. For transparency, we also clarified the error-bar definition in the figure captions by adding the following sentence: "*Error bars are calculated using standard error propagation.*"

(6) It is not clear whether the polycrystalline Mn samples do show any emergence of antiferromagnetic order. Extended Data Figure 1 shows resistivity minima in polycrystalline Mn at ~ 225K. Considering the resistivity minima to be a signature of onset of antiferromagnetic order (as stated by the authors), no corresponding sharp drop is seen temperature dependence of orbital Hall conductivity.

Response) We thank the reviewer for raising this important point and for carefully examining the relation between the resistivity minimum and the temperature dependence of the orbital Hall conductivity in polycrystalline Mn.

We would first like to clarify a key point that may have led to confusion. In the manuscript, the relation between a resistivity minimum and the onset of antiferromagnetic (AFM) order is discussed specifically for epitaxial α-Mn, where this connection is established in the literature. In particular, α-Mn thin films are known to exhibit a characteristic resistivity anomaly at the paramagnetic-to-antiferromagnetic transition, as demonstrated in previous studies [F. Boakye *et al.*, *Thin Solid Films* **221**, 224–227 (1992)]. Importantly, in our work the identification of AFM order in α-Mn does not rely on the resistivity minimum alone, but is corroborated by the simultaneous emergence of negative and anisotropic magnetoresistance below $T_N \approx 125$ K, as discussed in Supplementary Note 3.

For polycrystalline Mn, however, we did not interpret the resistivity minimum as a direct signature of antiferromagnetic ordering. As shown by our X-ray diffraction (XRD) data (**Fig. R3**), the polycrystalline films consist of multiple crystallographic phases rather than a single α-Mn phase, independent of growth temperature. The double-diffraction peak centered at $2\theta \approx 135°$ (green circle) can be indexed exclusively with the γ-Mn reference reflections (222) and (402), consistent with the presence of the (111) γ-Mn reflection at lower angle (purple circle). Combined with the observation of α-Mn crystallites in TEM, these XRD results provide evidence that the polycrystalline Mn films consist of multiple structural phases. In such a structurally heterogeneous system, the correspondence between a resistivity minimum and a well-defined AFM transition cannot be assumed. Instead, the resistivity minimum around ~225 K can originate from metallic-glass-like transport behavior and enhanced spin-fluctuation scattering, as reported previously for amorphous and polycrystalline Mn systems [F. Boakye, *J. Non-Cryst. Solids* **249**, 189 (1999)].

This distinction is further supported by magnetotransport measurements. In epitaxial α-Mn, the longitudinal magnetoresistance exhibits a clear qualitative change across T_N, where the parabolic Hanle response observed at high temperature evolves into hysteretic and anisotropic features upon cooling, consistent with the onset of AFM order. In contrast, the polycrystalline Mn samples display a parabolic Hanle-like magnetoresistance even below the resistivity-minimum temperature of ~225 K. No hysteresis or anisotropic magnetoresistance characteristic of long-range AFM ordering is observed within our measurement sensitivity.

To further provide an independent verification of the magnetic state in polycrystalline Mn, we performed additional SQUID magnetometry measurements on a 10 nm polycrystalline Mn film grown on Si_3N_4 and directly compared the results with epitaxial α-Mn (**Fig. R4**). The epitaxial Mn film exhibits a clear paramagnetic signal at room temperature that progressively decreases upon cooling and vanishes near the Néel temperature, consistent with the onset of AFM order. In contrast, the polycrystalline Mn film shows no detectable temperature-dependent magnetic signal between 10 K and 300 K, with the measured moment remaining below ~1 μemu, close to the instrumental resolution limit.

Taken together, within the sensitivity of our magnetotransport and SQUID measurements, we find no evidence for long-range AFM ordering in the polycrystalline Mn films. Accordingly, the absence of a

sharp anomaly in the temperature dependence of the orbital Hall conductivity is consistent with the magnetic behavior of these samples. While short-range correlations or phase-specific magnetic contributions cannot be entirely excluded in a multiphase system, our data indicate that the resistivity minimum observed in polycrystalline Mn is not associated with a well-defined bulk AFM transition.

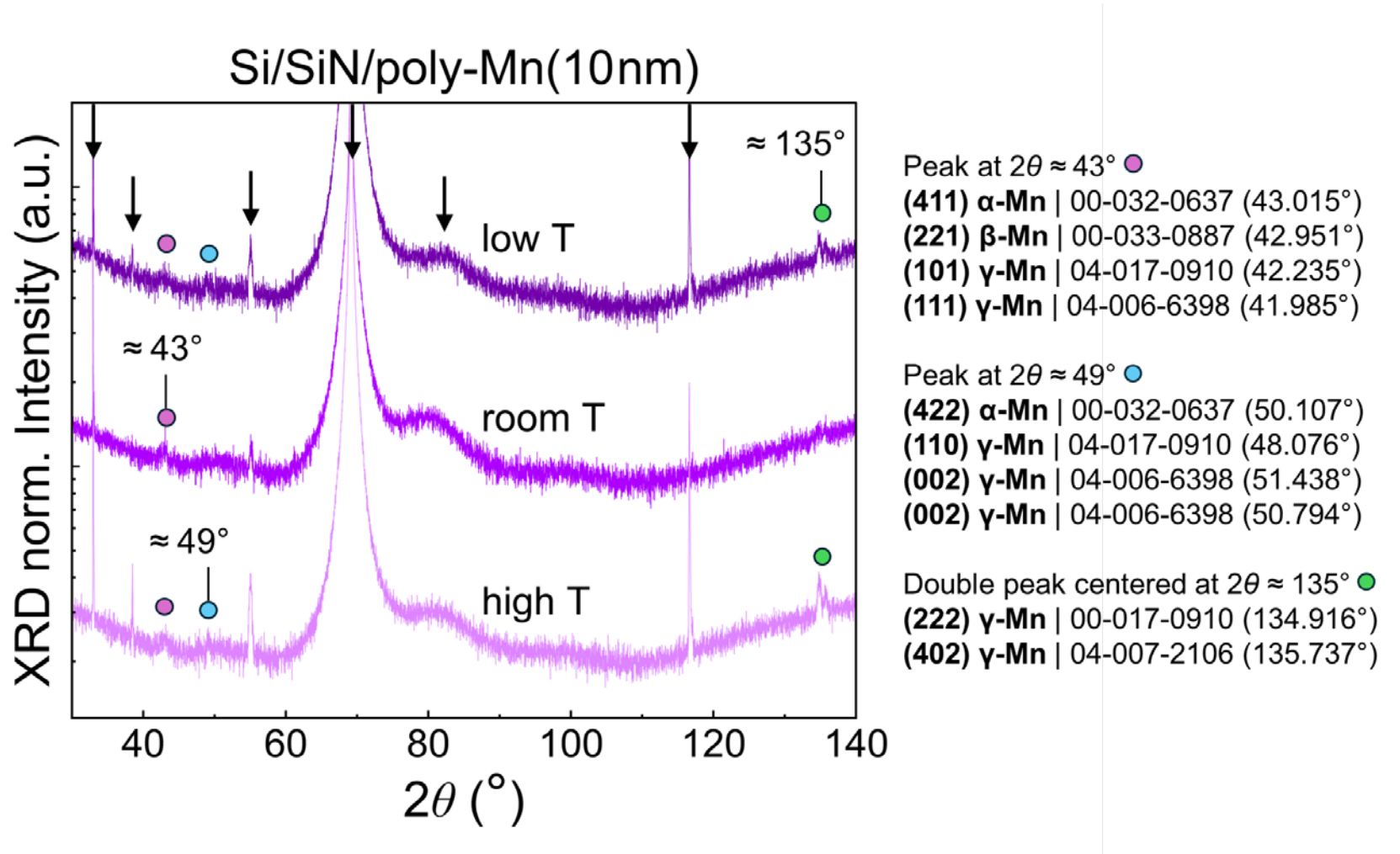

Figure R3 | XRD $\theta/2\theta$ scans of polycrystalline Mn films grown at different Si/SiN substrate temperatures. The diffractograms show multiple diffraction peaks corresponding to different Mn phases. Circular markers indicate peaks belonging to the Mn film, whereas black arrows indicate peaks belonging to the substrate. The legend includes reference peak positions obtained from the Powder Diffraction Database. Small deviations of the diffraction peak positions from the reference values may result from variations in the lattice parameters in thin films, as well as from differences in growth conditions.

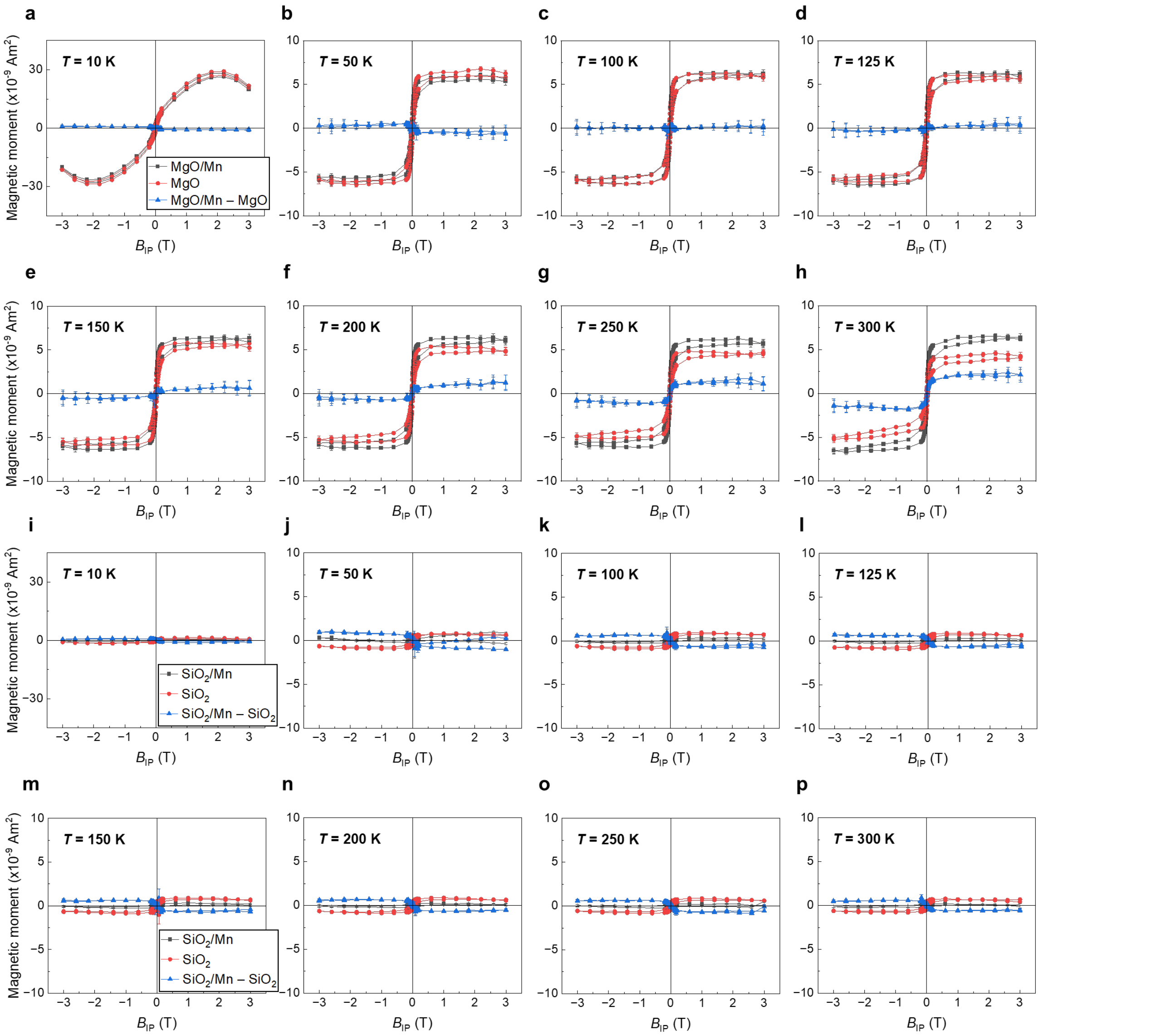

Figure R4 | Magnetic characterization of epitaxial and polycrystalline Mn using SQUID. a-h, Magnetization curves (magnetic moment vs. in-plane magnetic field B_{IP}) measured with B_{IP} applied along MgO[100]‖Mn[110] for 10-nm-thick epitaxial Mn(001) single layer (black curves), MgO(001) substrate reference (red curves), and their difference (blue curves). **i-p,** Magnetization curves (magnetic moment vs. in-plane magnetic field B_{IP}) measured with B_{IP} for 10-nm-thick polycrystalline Mn single layer (black curves), SiO_2 substrate reference (red curves), and their difference (blue curves). Different panels correspond to different temperatures

(7) Cross-section transmission electron microscopy showing the crystalline nature of Mn and comparison of the crystal structure with the data from X-ray and RHEED might help to substantially improve the quality of the manuscript.

Response) We thank the reviewer for this constructive suggestion. We fully agree that direct cross-sectional transmission electron microscopy (TEM) provides complementary structural information to

X-ray diffraction (XRD) and reflection high-energy electron diffraction (RHEED). Following this suggestion, we performed cross-sectional high-angle annular dark-field scanning TEM (HAADF-STEM) measurements on representative Mn films spanning the structural range studied in this work, including epitaxial, polycrystalline, and amorphous samples. The results are presented in **Fig. R5**, showing representative large-, medium-, and small-scale images of epitaxial, polycrystalline, and amorphous films, together with the corresponding Fourier transforms (FTs) of the medium-scale images.

In the epitaxial film, atomic-resolution HAADF-STEM images and corresponding FTs reveal a well-ordered lattice viewed along the α-Mn [011] zone axis, consistent with a film oriented with the [001] direction out of plane. These observations independently confirm the single-crystalline α-Mn phase identified by RHEED and X-ray diffraction.

In the polycrystalline film, cross-sectional images show a granular microstructure composed of crystalline grains with lateral dimensions larger than the film thickness, separated by well-defined grain boundaries. High-resolution imaging of representative grains exhibits lattice periodicities consistent with α-Mn (see, e.g., a grain imaged along the α-Mn [111] direction). The corresponding FTs display well-defined diffraction spots from individual grains with multiple discrete orientations. The presence of high-order diffraction spots indicates long-range crystalline order within each grain.

In the nanocrystalline-amorphous films, cross-sectional images evidence the absence of long-range crystalline order. The FTs of these films are characterized by a broad diffraction halo at the primary reflection order and negligible secondary reflections, consistent with an amorphous-like structure. Distinct nanoscale correlation structures are observed within the amorphous films depending on the Ar sputtering pressure. The 4 mTorr film shows a continuous diffuse diffraction halo and relatively uniform nanometric contrast, indicating a relatively narrow and homogeneous distribution of correlation lengths below ~4 nm. HAADF-STEM images show that the correlation length is about 1 nm in most of the film. In contrast, the 40 mTorr film exhibits a spotty diffraction halo and heterogeneous nanocrystalline contrast spanning ~1–10 nm, consistent with a broader distribution of structural correlation lengths and increased microstructural disorder.

These observations are consistent with the pressure-dependent disorder length scale inferred from transport measurements (Fig. R1(a)), indicating that lower sputtering pressure yields a more homogenous distribution of nanoscale structural correlations.

The new TEM observations therefore confirm a consistent structural description for the different samples investigated in this work, in agreement with the other characterization methods: (i) epitaxial films: single-crystalline α-Mn confirmed by RHEED, XRD, and TEM; (ii) polycrystalline films: multi-grain Mn containing α-Mn crystallites, consistent with TEM and multi-phase XRD; (iii) amorphous films: nanostructurally disordered Mn confirmed by TEM and XRD, with TEM- and transport-derived

correlation lengths, both pressure-dependent. Importantly, all these structural characterization methods provide results consistent with transport-derived disorder metrics.

These results substantially strengthen the structural basis of the manuscript and directly corroborate the disorder-dependent transport behavior discussed in the main text. The corresponding TEM analysis has been incorporated in the revised manuscript (Fig. 1c) and in the Supporting Information (Fig. S4), where the microstructural differences between amorphous films grown at different sputtering pressures are discussed.

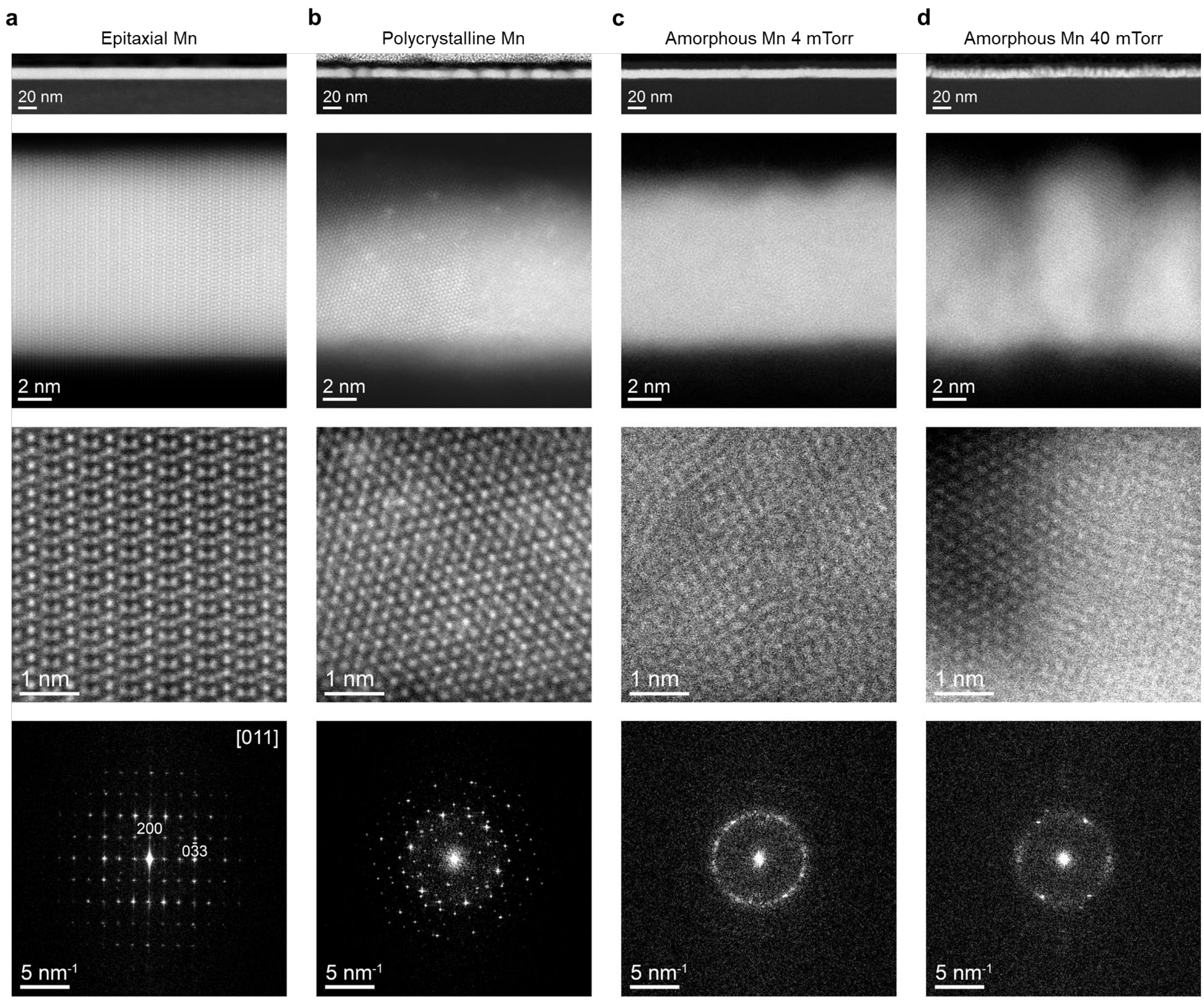

Figure R5 | Transmission electron microscopy of Mn thin films with varying structural order. From top to bottom: low-magnification HAADF-STEM images providing an overview of the Mn films; medium-magnification images highlighting microstructural details; atomically resolved images showing structural features at the atomic scale; Fourier transforms of the medium-magnification images. **(a)** Epitaxial α-Mn, **(b)** polycrystalline Mn, **(c)** nanocrystalline-amorphous Mn grown at 4 mTorr, and **(d)** 40 mTorr.

Reviewer #2 (Remarks to the Author):

Overall comments.

Overall, this manuscript provides a careful and systematic experimental study of orbital transport in disordered metallic systems. By controlling disorder, the authors extract both the orbital Hall conductivity (OHC) and the orbital relaxation time with good precision, extending the investigation into the bad-metal regime that has been largely unexplored in prior work. From this dataset they report (i) an approximately universal scaling in which the OHC increases linearly with longitudinal conductivity, (ii) a counter-intuitive bad-metal regime where stronger disorder is accompanied by a longer orbital relaxation time, and (iii) unravel new orbital relaxation channel which is related to phonon dynamics. These are valuable empirical observations that help map out orbital transport across a wide disorder range. That said, the overall impact is limited for several reasons. First, the claimed universal scaling is not convincingly established: its generality across temperatures and structural regimes remains unclear. Second, the main qualitative trend that orbital transport can improve as sample quality degrades has been reported in earlier study [Nat. Mat., 24, 1749-1755 (2025)], so the central message here does not appear conceptually new. Third, the phonon-based interpretation of orbital generation and relaxation is novel and interesting; however, the manuscript lacks a sufficiently developed theoretical explanation that would convincingly support the experimental observations. For these reasons, I find the experimental results promising and potentially impactful, but the manuscript does not seem to offer sufficient conceptual novelty to meet the expectations for a Nature Physics.

Response) We thank the reviewer for evaluating our work and for recognizing its interest and potential impact. We particularly appreciate the acknowledgment that our approach enables the quantitative extraction of both the orbital Hall conductivity and the orbital relaxation time, and that it extends orbital transport measurements into the bad-metal regime, which has remained unexplored so far. We also agree with (i)-(iii), but disagree with the partial lack of conceptual novelty attributed to these findings. Both (ii) and (iii) are conceptually novel, whereas universal scaling of orbital effects (i) has never been proved in the strong disorder regime so far. We comment below on the three points raised be the referee.

Universal scaling: We agree that universal scaling was not properly articulated in the first version of the manuscript, and thank the referee for the detailed elaboration of their criticism in point [1] of their report. In response to this criticism, we have better defined the type of universal scaling that we refer to in the manuscript, and made new samples and measurements to substantiate the different scaling regimes investigated in our work. A detailed response to this criticism is given in the reply to point [1].

Comparison with results reported in Ref. 35 [Nat. Mat., 24, 1749-1755 (2025)]. This paper concerns a study of orbital torques in $SrRuO_3$/Ti/Pt/Co/Pt heterostructures. While this is an interesting study, our work differs from Ref. 35 in several crucial points.

Reference 35 reports an effective Hall conductivity σ_{eff} that increases when reducing the conductivity σ_{xx} of $SrRuO_3$/Ti/Pt/Co/Pt, both at room temperature (by varying the oxygen pressure during sputtering) and as a function of temperature. The orbital Hall conductivity σ_{OH} (corresponding to our σ_L) derived from these measurements after subtraction of σ_{eff} measured in a reference $SrRuO_3$/Ti/NiFe sample with dominant spin Hall effect, is then argued to decrease sharply with σ_{xx} between 8-10 x 10^5 Ω^{-1} m^{-1} and then to saturate at a constant level for $\sigma_{xx} > 10 \times 10^5\ \Omega^{-1}\ m^{-1}$, see Figure 3 in Ref. 35. This trend is opposite to what we report in the strongly-disordered regime, namely σ_L increasing with σ_{xx}, as summarized in **Fig. R6**. Furthermore, the orbital relaxation time is not measured in Ref. 35. This paper infers a Dyakonov-Perel orbital relaxation mechanism from the decrease of σ_{eff} vs σ_{xx}.

These observations underscore the difference between the two studies:

1) Our work spans a much lower and broader conductivity range than Ref. 35, from 10^2 to 10^4 $\Omega^{-1}cm^{-1}$, accessing orbital generation and relaxation in the hopping-dominated disorder regime and reaching up to conventional metallic conduction within a single material system. We focus on the "bad metal" end of the conductivity spectrum, in which extrinsic effects, such as skew scattering and Dyakonov-Perel relaxation discussed in Ref. 35, do not play a role. The monotonic increase of orbital Hall conductivity with increasing longitudinal conductivity has never been observed before and is characteristic of a hopping-dominated transport regime. We note also that in the epitaxial and textured Mn films with higher conductivity and normal metallic behavior, we observe a qualitatively similar but weaker temperature dependence as in Ref. 35, namely σ_L decreases modestly as σ_{xx} increases. Thus, our study does not overlap or contradict Ref. 35, it addresses different transport regimes.

2) Beyond conductivity scaling, our approach allows us to extract the orbital relaxation times and correlate orbital relaxation with crystallinity and disorder (**Fig. R6c**). In the bad-metal regime (amorphous to polycrystalline Mn), the orbital relaxation time decreases with increasing conductivity, consistent with hopping-mediated motional narrowing of the crystal-field interaction in a strongly disordered system. This type of orbital relaxation has not been postulated before. Further, upon increasing crystallinity toward textured and epitaxial Mn, this trend reverses: higher conductivity correlates with a longer orbital relaxation time, indicative of a crossover to Elliott–Yafet-like relaxation. This crystallinity-driven transition in orbital relaxation behavior has also not been reported previously.

3) An additional distinction with Ref. 35 and other experimental reports lies in the experimental methodology. Most studies of orbital conductivity and related phenomena, including work from our group, have been performed indirectly, by measuring orbital torques or orbital pumping in layered nonmagnetic/magnetic structures, where interface transmission and orbital-to-spin conversion play a major role. Likewise, Ref. [35] infers orbital transport from torque measurements in $SrRuO_3$/Ti/Pt/Co/Pt and (spin) torque measurements in $SrRuO_3$/Ti/NiFe heterostructures, where bulk orbital generation is inherently entangled with interfacial transmission, possible contributions from Pt, and magnetic-layer responses. In contrast, our experiments are performed on a single Mn layer and use orbital Hanle

magnetoresistance as a direct probe of orbital accumulation and relaxation within the same material. This approach eliminates magnetic and interfacial contributions from other elements and allows both the orbital Hall conductivity and the orbital relaxation time to be extracted within a single, self-consistent framework. The ability to directly access the orbital relaxation time τ_L, which is not addressed in Ref. 35, constitutes a key advance of the present work.

4) We refrain from directly concluding on “orbital transport”, which is a highly controversial topic, focusing on orbital generation and relaxation instead.

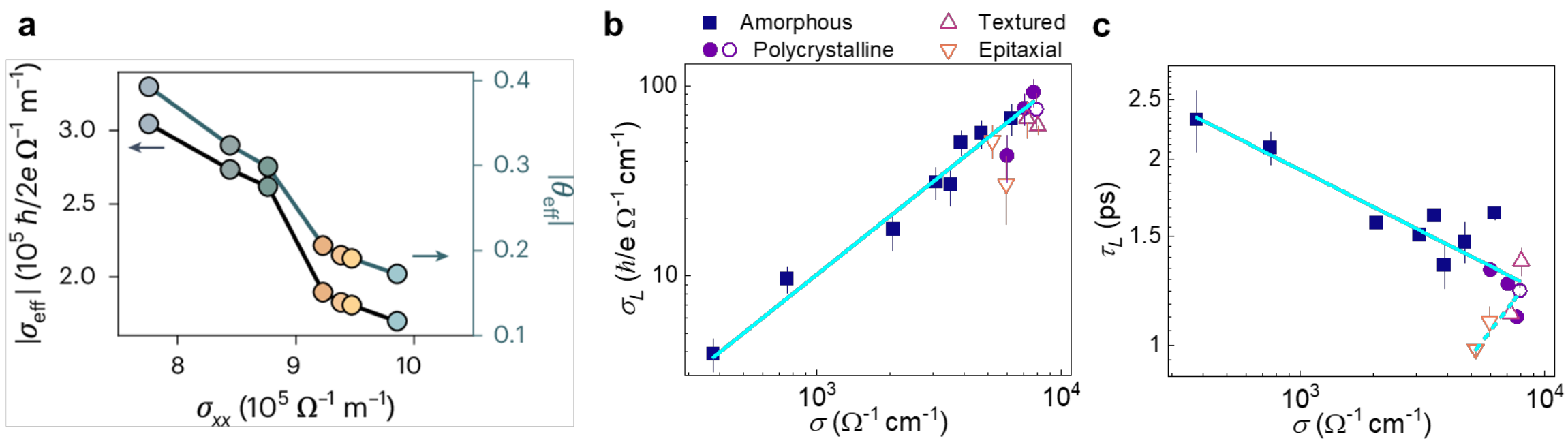

Figure R6 | Comparison of orbital Hall conductivity scaling at room temperature. **(a)** Effective orbital Hall response as a function of longitudinal conductivity at room temperature for $SrRuO_3$ thin films deposited under different oxygen pressures, reproduced from Ref. [35], Fig. 3c. **(b)** Orbital Hall conductivity σ_L and **(c)** orbital relaxation time τ_L as a function of longitudinal conductivity σ for Mn thin films with different degrees of static disorder at room temperature, reproduced from Fig. 3 of the original manuscript.

Lack of a detailed theoretical model of orbital relaxation in the disordered limit. We identify a novel disorder-dependent orbital decoherence mechanism, which, to our knowledge, has not been reported before, neither by theory nor by experiments. Our results thus call for developing theoretical models of orbital generation and relaxation in strongly disordered systems, which currently do not exist. While we agree with the referee that developing a quantitative model of orbital relaxation in the strongly disordered regime is important, we also believe that the lack of a fully developed theory for novel experimental results could be considered as a positive rather than negative factor. Physics would be extremely boring if all experiments would nicely fit in existing theoretical models! A more detailed response to this criticism is given in the reply to point [3].

Overall, we believe that our work provides a new perspective on how orbital generation and relaxation evolve across the disordered and metallic regimes, which is complementary to studies such as Ref. 35

that focus on a different conductivity regime and different relaxation mechanisms. The ability to access both orbital generation and relaxation across multiple transport regimes within a single material platform, identifying novel mechanisms related to disorder, constitutes a central strength of this work.

Questions to authors.

[1] On the linear scaling of OHC with conductivity. The authors argue that, upon moving from epitaxial to amorphous samples, both the orbital generation and relaxation mechanisms change qualitatively (e.g., from an intrinsic orbital Hall effect (OHE) picture to phonon-activated hopping–induced orbital generation). Given that the crystal structures are also substantially different across these regimes, it is not obvious why the OHC would still follow the same linear scaling with conductivity. For example, comparing Fig. 4(b) and Fig. 4(f), the former is interpreted as phonon-mediated hopping–induced OHE, whereas the latter is attributed to intrinsic OHE. The conductivity ranges are relatively close (5.97- 6.0 * 10^3 ohm^-1 cm^-1) vs. (5.22- 5.30 * 10^3 ohm^-1 cm^-1), yet the OHC behaves very differently: in Fig. 4(b) it varies strongly (13- 40 hbar/e ohm^-1 cm^-1), while in Fig. 4(f) it remains nearly constant (40- 50 hbar/e ohm^-1 cm^-1). This seems to suggest that the OHC– conductivity linearity may hold at some temperatures or in some regimes, but not universally. Therefore, I have a question: Could the authors clarify the scope of their claim? Specifically, do they argue that a linear OHC–conductivity scaling is universal even when the lattice structure and the orbital generation mechanisms differ substantially, or do they mean that such linear scaling emerges only within the phonon-mediated hopping regime? If the former is intended, I believe stronger evidence would be necessary to substantiate this universality—for instance, by demonstrating that the scaling is temperature-independent across the full range studied (rather than holding only at room temperature or in a limited regime), by showing that a similar linear trend persists in Mn films with thicknesses different from those used here, or by providing a clear microscopic theoretical justification.

Response) We thank the reviewer for highlighting this important point and allowing us to clarify it. Indeed, we do not argue that the orbital Hall conductivity follows the same scaling with longitudinal conductivity across all regimes. We fully agree that, given the substantial differences in crystal structure and orbital generation mechanisms between epitaxial, textured, polycrystalline, and amorphous Mn, different "universal" scalings should be observed in different regimes, similar to the scalings reported for the anomalous Hall and spin Hall conductivities in the hopping, intrinsic (moderately dirty), and ultraclean regimes.

By "universal," we did not intend to claim that a single linear scaling of the orbital Hall conductivity applies identically across all crystal structures and transport mechanisms. Rather, our use of the term refers to the emergence of orbital Hall conductivity within the same scaling framework that governs other transverse transport phenomena, such as the anomalous Hall effect and the spin Hall effect, in the

low-conductivity, disorder-dominated regime. In this regime, theoretical and experimental studies have shown that the transverse response follows a power-law scaling, $\sigma_{xy} \propto \sigma^{\gamma}$, with γ typically ranging from ~1.0 to ~1.8 depending on the specific hopping mechanism and degree of disorder. While this framework has been extensively tested for anomalous and spin Hall responses across a wide range of materials, its applicability to orbital Hall transport has remained essentially unexplored, particularly in the bad-metal and hopping-dominated regimes. A central claim of our work is that the orbital Hall conductivity follows this same disorder-controlled scaling framework in the hopping-transport regime, thereby extending Hall universality to orbital angular momentum for the first time. In this sense, "universal" refers to the shared scaling phenomenology within a specific transport regime, rather than to an identical behavior across fundamentally different regimes such as metallic band transport and thermally activated hopping.

We agree that, in the original manuscript, the fitting range was not well specified, which may have given the impression that a single scaling law was claimed across all transport regimes. Upon revisiting the analysis, we clarify that the power-law scaling $\sigma_L \propto \sigma^{\gamma}$ was intended in describing the hopping-dominated bad-metal regime, corresponding to the amorphous and polycrystalline Mn samples. When the fitting is restricted to this regime, we obtain an exponent $\gamma = 1 \pm 0.2$. This value is consistent with the experimentally reported range ($\gamma \approx 1.0$–1.8) for anomalous and spin Hall conductivities in disordered systems. Importantly, while the extracted exponent remains close to unity, explicitly restricting the fitting range makes the physical scope of the scaling relation unambiguous. The scaling therefore applies specifically to the hopping-transport regime, rather than implying universality across metallic and non-metallic regimes. We believe this clarification strengthens the manuscript by aligning the data analysis more closely with the underlying transport physics.

The reviewer's comparison between Figs. 4(b) and 4(d) underscores another central point of our study. Although the conductivity ranges in these panels are numerically similar, the underlying transport mechanisms are fundamentally different. In amorphous and polycrystalline Mn [Figs. 4(a,b)], charge transport is governed by thermally-activated hopping, and the orbital Hall response is therefore dominated by phonon-assisted, disorder-compatible processes. In this regime, no theory of orbital generation and transport is presently available. However, based on the theory and previous experiments on the anomalous and spin Hall effects, we expect that the Hall response can increase with conductivity as hopping becomes more efficient, consistent with the strong variation of σ_L observed in Figs. 4(a,b). In contrast, textured and epitaxial Mn [Figs. 4(c,d)] reside in the conventional metallic transport regime, where carriers propagate through extended states and orbital Hall generation is predominantly intrinsic in origin. In this case, σ_L is expected to depend only weakly on conductivity, which naturally explains the nearly constant orbital Hall conductivity observed despite modest changes in σ. The partial overlap of room-temperature data points between the hopping and metallic regimes is therefore coincidental, rather than indicative of a single universal scaling law.

To further substantiate this interpretation, we have performed additional measurements on metallic Mn samples, including epitaxial Mn films grown under different conditions, epitaxial Mn with different thicknesses, and textured Mn prepared on substrates with different surface treatments. Across these metallic samples, σ_L consistently shows behavior distinct from that of the hopping-dominated films, reinforcing the conclusion that the linear $\sigma_L \propto \sigma^\gamma$ scaling is specific to the bad-metal regime. A detailed discussion of these additional datasets and their implications for orbital relaxation mechanisms is provided in our response to Question 4.

Based on the considerations above, we have revised the presentation of our data to avoid any possible overgeneralization. In the revised manuscript, we now explicitly state that the linear scaling between the orbital Hall conductivity and the longitudinal conductivity applies to the hopping-dominated bad-metal regime, rather than to all transport regimes. Accordingly, the term "universal" is used to emphasize that, within this low-conductivity regime, orbital Hall transport follows the same scaling framework previously established for the anomalous and spin Hall effects, thereby extending this phenomenology to orbital angular momentum. To reflect this clarification, we have revised the Abstract to specify the transport regime in which the scaling holds. In addition, in the discussion of Fig. 3, we now explicitly indicate that the power-law fit is performed using the amorphous and polycrystalline samples, yielding an exponent $\gamma = 1 \pm 0.2$, and we clarify that metallic samples are discussed separately. Finally, we have adjusted the wording in the Discussion section to distinguish more clearly between hopping-dominated and metallic transport regimes, and to emphasize the regime-dependent nature of orbital Hall generation and relaxation. We believe that these revisions directly address the reviewer's concerns by clarifying the intended scope of our claims.

[2] Extrinsic OHE. For the extrinsic orbital Hall effect observed in the amorphous regime, can the authors clarify which mechanism they attribute it to (phonon-mediated skew scattering vs side jump)?

Response) We thank the reviewer for raising this important question regarding the microscopic origin of the orbital Hall response observed in the amorphous regime.

Within conventional band-transport theory, extrinsic transverse responses are indeed commonly classified in terms of skew scattering or side-jump mechanisms. However, we emphasize that this classification is formulated for systems with extended Bloch states and well-defined impurity scattering events. It therefore cannot be directly applied to amorphous Mn, where charge transport is governed by thermally activated hopping between localized states rather than band-like transport.

In disordered and hopping-dominated systems, extrinsic transverse transport is instead understood within a different theoretical framework that does not rely on skew or side-jump scattering. As discussed in Refs. [45–47, 50–62], anomalous Hall and spin Hall responses in the hopping regime arise from quantum interference effects associated with multi-site hopping processes, such as closed loops or

hopping triads. In this picture, a finite transverse response emerges from the electron's phase accumulated around a loop of localized states in the presence of spin–orbit coupling and an applied electric field. Importantly, this mechanism naturally leads to a power-law scaling of the transverse conductivity with the longitudinal conductivity, $\sigma_{xy} \propto \sigma^{\gamma}$, without invoking skew or side-jump scattering in the conventional sense. Our observation of a well-defined scaling of the orbital Hall conductivity in amorphous Mn is qualitatively consistent with such a hopping-based phenomenology, with the important distinction that spin-orbit coupling is not required to establish an orbital response. We have confirmed the plausibility of this interpretation with theoretical colleagues involved in calculating spin responses in disordered systems, who have also expressed future interest in extending their models to orbital transport and relaxation.

We therefore interpret the orbital Hall response in the amorphous regime as a hopping-mediated, disorder-driven effect that belongs to the same class of transport phenomena as hopping-induced anomalous and spin Hall effects, rather than attempting to map it onto skew-scattering or side-jump mechanisms defined for band transport. We should also stress that extrinsic contributions due to skew scattering scale with momentum relaxation and only become dominant in the ultraclean metal regime, which is on the opposite end of the conductivity range addressed in our study. For the same reason, we do not consider Dyakonov-Perel relaxation in our analysis, since this is expected to become prominent only in the high conductivity limit of good metals.

The role of phonons in the disordered regime also merits clarification. In the amorphous films studied here, phonons do not act as a primary driving force for orbital current generation. Rather, they provide the necessary incoherent thermal assistance that enables electron hopping between localized states. The orbital Hall response itself remains fundamentally electric-field-driven, arising from the transverse orbital accumulation associated with hopping loops. In this sense, the term "phonon-mediated" refers to phonon-assisted hopping in the presence of an applied electric field, and should be clearly distinguished from coherent phonon-driven effects or dynamical phonon pumping mechanisms. The relevant phonons are incoherent thermal (predominantly acoustic) phonons that facilitate charge motion, not coherent lattice excitations that directly generate orbital angular momentum.

To clarify this interpretation in the main text, we added the following sentence in the discussion of Fig. 3 (page 6, lines 194-197): "*In this strongly disordered regime, charge transport occurs via thermally activated hopping between localized electron states. The orbital Hall response is therefore interpreted as a hopping-mediated transverse accumulation of orbital momenta, analogous to hopping-induced anomalous and spin Hall effects [45–47], rather than in terms of skew or side-jump scattering of Bloch electrons.*"

[3] Microscopic mechanism of phonon-induced orbital relaxation. While the phonon-activated relaxation interpretation is interesting, it currently reads as speculative. Please provide a clearer theoretical model or independent evidence supporting this claim.

Response) We thank the reviewer for noting the interest of our interpretation. We agree that the microscopic origin of the temperature-dependent orbital relaxation inferred from our Hanle magnetoresistance analysis should be presented as an interpretation, because a microscopic theory for *orbital* relaxation in the strongly disordered regime is not yet available, despite increasing attention devoted to disorder effects, see, e.g., Refs. [26-31]. That said, our "phonon-activated" interpretation is not merely a speculative add-on, but rather a phenomenological interpretation that directly follows from the *measured* nonmonotonic behavior of the orbital relaxation time and its systematic correlation with thermally activated transport, as seen in Fig. 4.

In amorphous and polycrystalline Mn films, where the conductivity is thermally activated (negative TCR at room temperature), we find that the orbital relaxation time τ_L exhibits a nonmonotonic temperature dependence: τ_L initially increases as temperature (and conductivity) increases, but then decreases at higher temperature (Fig. 4e,f). This behavior provides independent evidence that at least two distinct orbital decoherence channels compete as thermal disorder increases. In the low-temperature limit of the hopping regime, transport is slow and electrons remain localized for longer times on a given site; in this case, orbital dephasing can occur predominantly on-site via precession in locally varying crystal-field environments. We use the hopping time scale τ_h (with $\tau_h \propto 1/\sigma$ in activated transport) as the relevant transport time, so that longer localization times (larger τ_h) naturally enhance local dephasing and reduce τ_L. As temperature increases and hopping becomes faster, the effective random local fields experienced by carriers can be partially averaged ("motional narrowing"), which leads to an increase of τ_L, precisely the trend observed in the low-to-intermediate temperature range.

At higher temperature, however, the accelerating hopping rate also increases the probability of orbital-non-conserving hopping events, i.e., orbital wavefunction mixing during inter-site transitions, which provides an additional relaxation channel that becomes dominant and drives τ_L downward (the high-temperature branch in Fig. 4e,f). Importantly, in our picture phonons do not "create" orbital angular momentum directly; rather, incoherent thermal phonons enable hopping and therefore tune the balance between (i) on-site dephasing during localization and (ii) hopping-induced orbital decoherence once hopping becomes sufficiently frequent. This is the specific meaning of "phonon-activated" should be understood as phonon-assisted hopping in an electric-field-driven transport response, not as a coherent phonon-driven orbital pumping mechanism.

We also note that this two-channel phenomenology is similar to the phenomenology of spin relaxation in systems lacking coherent band transport, where increasing hopping rate can first suppress local dephasing via motional narrowing and later introduce additional spin relaxation via hopping-related mechanisms. This behavior has been treated extensively in the context of organic/disordered conductors

and hopping transport, including motional-narrowing scenarios and the crossover between distinct spin-relaxation channels. We cite Refs. [51–54] and [60–63] (already included in our manuscript) as representative examples of this framework.

Finally, the ordered (metallic) samples provide a clear internal consistency check: in epitaxial α-Mn, τ_L increases approximately linearly with conductivity σ(T) (Fig. 4h), consistent with Elliott–Yafet-like relaxation in the metallic diffusive regime, whereas the disordered films show the qualitatively different, nonmonotonic τ_L behavior described above. This sharp contrast across transport regimes supports our interpretation that the temperature dependence of τ_L in disordered films is governed by hopping physics (and thus phonon-assisted transport), rather than being an arbitrary fitting artifact.

In summary, while we agree that a fully microscopic orbital-relaxation theory for hopping systems is still missing, our interpretation is grounded in (i) the experimentally observed nonmonotonic $\tau_L(T)$ in hopping films, (ii) its systematic correlation with activated transport, and (iii) a well-established relaxation phenomenology in hopping systems (by analogy to spin).

[4] On the Elliott–Yafet (EY) mechanism. In Fig. 3(b), the assignment of the epitaxial and textured samples to an EY-type relaxation appears to rely on only two data points, which seems insufficient to draw a firm conclusion. Could the authors provide stronger evidence to substantiate the EY interpretation in the epitaxial and textured samples?

Response) We thank the reviewer for raising this important point regarding the assignment of Elliott-Yafet (EY)-like orbital relaxation in the metallic Mn samples. We agree that, when viewed solely from Fig. 3(b) of the original manuscript, the number of data points associated with the metallic regime appeared to be limited. However, the data points included in the original analysis were actually three, including epitaxial, textured, and one polycrystalline sample showing a positive temperature coefficient of resistance (TCR). The TCR serves as a straightforward discrimination criterium to distinguish between hopping-dominated transport (TCR<0) and band-like transport (TCR>0), in agreement with other studies [see Refs. 40-42]. This wasn’t well explained in the text. More importantly, the referee’s comment motivated us to perform additional experiments designed to test the scaling behavior of the orbital relaxation time in more Mn samples with metallic behavior, and to examine whether the observed trend is robust across different growth conditions, thicknesses, and structural realizations.

To address the reviewer’s concern, we performed two independent sets of additional experiments, all focusing on epitaxial α-Mn films:

(i) Thickness dependence of epitaxial α-Mn (**Fig. R7**): We grew an additional epitaxial α-Mn film with a thickness of 20 nm, in comparison to the original 10-nm-thick sample. XRD measurements (**Fig. R7a**) confirm the same α-Mn(001) phase for both films. Using the same

orbital Hanle fitting procedure as in the main text, we extract the orbital relaxation time at room temperature. Upon increasing the conductivity σ from 5.22×10^3 to $6.09 \times 10^3\ \Omega^{-1}\mathrm{cm}^{-1}$, the orbital relaxation time τ_L increases from 0.98 ± 0.04 ps to 1.32 ± 0.10 ps. This clear increase of τ_L with conductivity within the same crystal phase directly supports EY-like behavior.

(ii) Substrate-engineered conductivity of epitaxial α-Mn **(Fig. R8):** We next investigated the robustness of the EY-like scaling within epitaxial α-Mn by tuning the conductivity through controlled modification of the MgO(001) substrate's surface. **Figure R8** compares three epitaxial α-Mn films, Mn_{LowT}, Mn_{RT1}, and Mn_{RT2}, all with the same thickness and crystal phase, but grown under different interfacial conditions. Mn_{RT2} was prepared by depositing Mn at room temperature after pre-annealing the MgO(001) substrate in a tube furnace under an oxygen atmosphere, followed by degassing of the substrate in UHV prior to Mn growth. In contrast, no tube-furnace treatment was performed for Mn_{RT1}, and Mn_{LowT} was grown at lower substrate temperature without furnace treatment. XRD measurements confirm that all three films remain in the epitaxial α-Mn phase (**Fig. R8a**). This controlled variation of growth and interface conditions leads to a systematic increase in conductivity from 5.22×10^3 to $8.03 \times 10^3\ \Omega^{-1}\mathrm{cm}^{-1}$ from Mn_{LowT} to Mn_{RT1}, and Mn_{RT2} (see Table R1). Importantly, the extracted orbital relaxation time τ_L increases monotonically from 0.98 to 1.37 ps across these samples. This trend reproduces the EY-like scaling observed in Fig. 3(b) of the main text within a single crystal phase, demonstrating that the scaling does not rely on changing structural classes.

When all metallic-regime data points, including the additional epitaxial samples introduced above, are combined, the scaling of orbital relaxation time with conductivity remains linear within uncertainty. As shown in **Fig. R9**, refitting $\tau_L \propto \sigma^\gamma$ yields $\gamma = 0.51 \pm 0.07$, essentially unchanged from the original value ($\gamma = 0.50 \pm 0.08$). Importantly, this scaling now rests on a substantially expanded dataset spanning multiple independent control parameters, film thickness, growth temperature, and substrate surface preparation, rather than on a small number of isolated samples.

Taken together, these additional experiments provide strong evidence that orbital relaxation in epitaxial and textured Mn follows an EY-like scaling in the metallic regime.

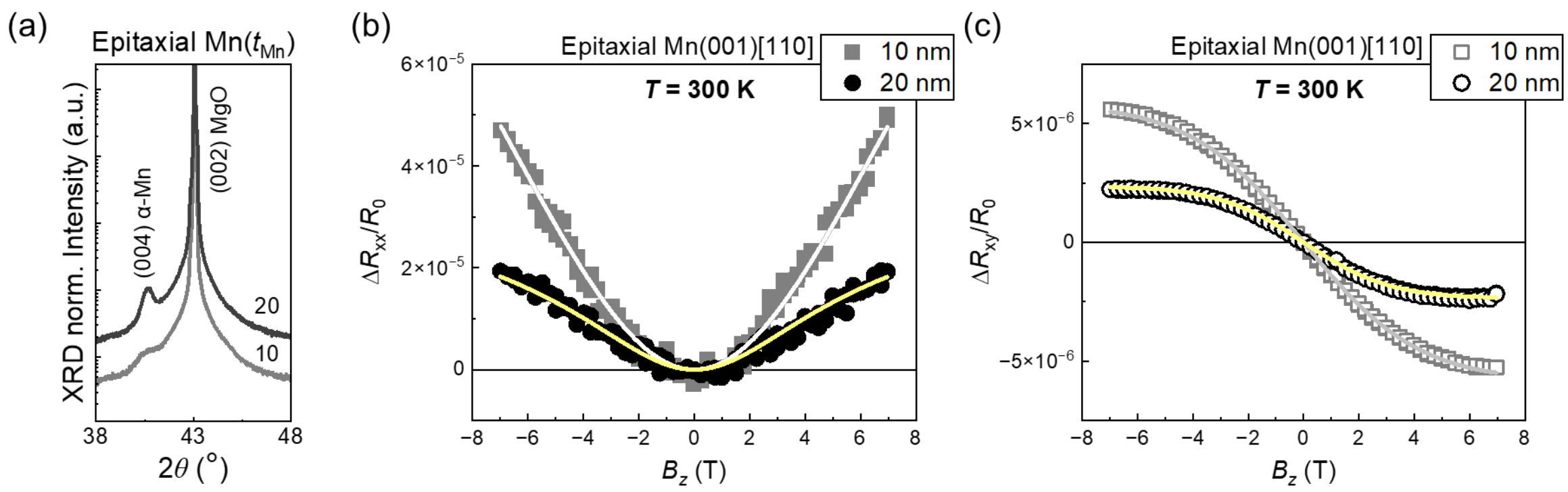

Figure R7 | Thickness dependence of orbital relaxation in epitaxial α-Mn. (a) X-ray diffraction (θ–2θ scan) of epitaxial α-Mn films with thicknesses of 10 nm and 20 nm grown on MgO, confirming the α-Mn(001) phase. **(b)** Longitudinal and **(c)** transverse Hanle magnetoresistance measured at room temperature for the two thicknesses. Solid lines are fits to the orbital Hanle model used in the main text. The extracted transport parameters are summarized in Table R1.

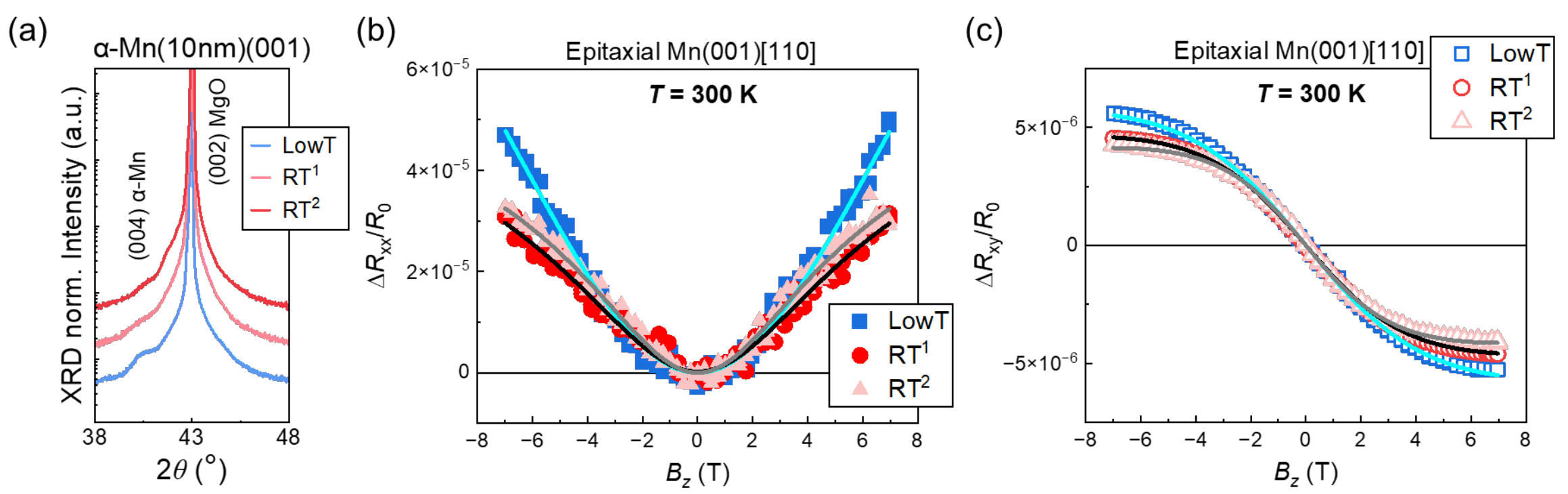

Figure R8 | Growth-temperature dependence of orbital relaxation in epitaxial α-Mn. (a) X-ray diffraction of 10 nm epitaxial α-Mn films grown at different substrate temperatures, confirming the α-Mn phase in both cases. **(b)** Longitudinal and **(c)** transverse Hanle magnetoresistance measured at room temperature. Solid lines represent fits to the orbital Hanle model. Extracted fitting parameters are listed in Table R1.

Samples	σ [$10^3\Omega^{-1}\text{cm}^{-1}$]	σ_L [$\hbar/e\ \Omega^{-1}\ \text{cm}^{-1}$]	τ [ps]	θ
Epi-Mn_{LowT} (10 nm)	5.22	52 ± 10	0.98 ± 0.04	0.019 ± 0.001
Epi-Mn_{LowT} (20 nm)	6.09	26 ± 12	1.32 ± 0.13	0.008 ± 0.005
Epi-Mn_{RT1} (10 nm)	5.95	31 ± 12	1.18 ± 0.02	0.010 ± 0.002
Epi-Mn_{RT2} (10 nm)	8.03	62 ± 11	1.37 ± 0.02	0.015 ± 0.005

Table R1 | Fitting parameters extracted from Hanle analysis. Extracted electrical conductivity (σ), orbital Hall conductivity (σ_L), orbital Hall angle (θ_{OH}), and orbital relaxation time (τ_L) extracted from Hanle fitting for epitaxial Mn films.

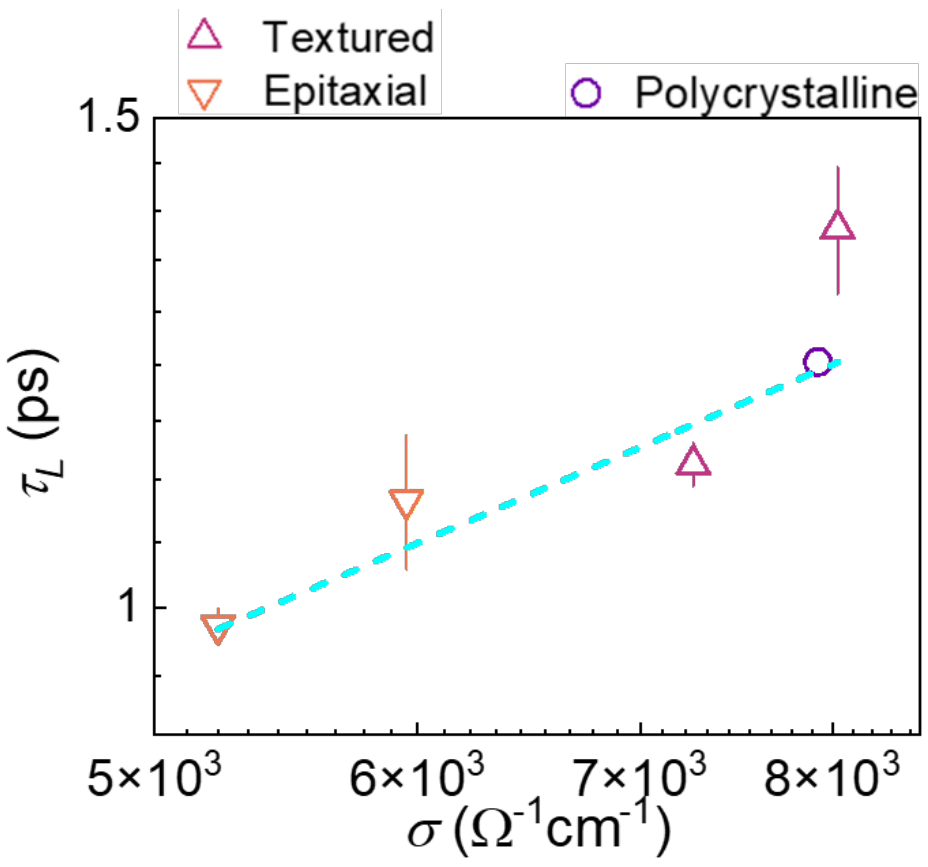

Figure R9 | Scaling of orbital relaxation time in metallic Mn at room temperature. Orbital relaxation time τ_L as a function of longitudinal conductivity σ for epitaxial, textured, and high-temperature-grown polycrystalline Mn, including all additional datasets shown in Figs. R8–R10. The solid line is a power-law fit $\tau_L \propto \sigma^{\gamma}$, yielding $\gamma \approx 0.5$. Error bars reflect fitting uncertainties.

Reviewer #3 (Remarks to the Author):

This manuscript presents a detailed and fundamental investigation of the orbital Hall conductance (OHC) in Mn compounds exhibiting different degrees of disorder and transport regimes, ranging from epitaxial metallic and textured films to polycrystalline and amorphous samples, all grown under various sputtering conditions. In this study, Mn is chosen as an ideal prototypical material owing to its very small spin Hall conductivity and its low equilibrium orbital angular momentum (singlet state). Using orbital Hanle magnetoresistance measurements performed over a wide temperature range, the authors rigorously demonstrate: (i) the existence and quantitative determination of the OHC in Mn, leading to orbital current generation and orbital polarization at the sample edges; (ii) the dependence of the OHC on disorder; and (iii) the dependence of the orbital relaxation (or decoherence) time on both disorder and phonon scattering rates (temperature). The main results of this work include the demonstration of an intrinsic OHC (i.e., conductivity-independent) in the epitaxial and textured Mn series, and a conductivity-dependent OHC in amorphous samples, where variable-range hopping transport dominates. In contrast, the orbital decoherence time exhibits a more complex behavior: it increases with hopping conductivity as disorder is varied, while it decreases when phonon scattering is enhanced at higher temperatures. This behavior points to a crossover in the dominant orbital decoherence mechanisms depending on the underlying scattering process.

In my view, the breadth of systems investigated and the depth of the experimental analysis are highly impressive, and the conclusions drawn are both robust and of significant value to the rapidly emerging field of orbitronics. As this field continues to grow, the results presented here will undoubtedly prove to be important and broadly useful to the scientific community.

I therefore conclude that this work merits rapid publication in Nature Physics, provided that the authors adequately address the comments and questions raised below.

Response) We thank the reviewer for the thoughtful assessment of our work and for noting the breadth and depth of our experimental results as well as their significant value to the emerging field of orbitronics.

We are grateful for the constructive comments and for the opportunity to further improve the manuscript. We have carefully considered all the concerns raised and revised the manuscript accordingly where appropriate. We hope that our responses and the corresponding revisions satisfactorily address the reviewer's questions and clarify the scope and significance of our results. Below, we provide detailed point-by-point responses to each comment.

1) One of my main concern relates to the quality of the Mn top surface investigated in the Hanle transport measurements. The manuscript does not address the possibility of slight oxidation or surface contamination of the Mn Hall bar, which could significantly alter—or even compromise—the measured magnetoresistance properties. This issue is neither discussed in the main text nor in the Supplementary

Information. Could the authors please comment on this important point and clarify how surface oxidation or contamination is controlled or ruled out?

Response) We thank the reviewer for raising this important point, which was also raised by Referee 1 and allows us to clarify an essential experimental aspect of our study. We agree that uncontrolled surface oxidation could significantly affect magnetotransport measurements, and we therefore took particular care to prevent any exposure of Mn to ambient conditions throughout fabrication and measurement.

For the MBE-grown epitaxial Mn samples, an Al capping layer with a thickness of approximately 3 nm was deposited *in situ* immediately after Mn growth, without breaking vacuum. This Al layer was subsequently oxidized under ambient conditions to form a stable AlO_x protective layer. After device fabrication, we additionally performed O_2 plasma ashing to ensure complete oxidation of the Al layer. This procedure guarantees a fully insulating and chemically stable AlO_x cap. The presence of this capping layer is already visible in Fig. 1(c); however, the fabrication details were insufficiently described in the original manuscript.

For all sputtered Mn samples, an ~8 nm thick SiN capping layer was deposited immediately after Mn growth by RF sputtering at an Ar pressure of 10 mTorr. As a result, the Mn layer was never exposed to air at any stage of processing or measurement.

Therefore, we exclude significant contribution from surface oxidation to the observed Hanle magnetoresistance signals.

To avoid any ambiguity, we have now explicitly revised the Methods section and added the following sentences (page 10, lines 300-305): "*To prevent surface contamination of Mn, MBE-grown epitaxial films were protected by an Al capping layer (~3–4 nm) deposited in situ immediately after growth, which was subsequently oxidized under ambient conditions. After device fabrication, an additional O_2 treatment was applied to ensure complete oxidation of the Al layer, forming a stable AlO_x cap. For sputtered Mn films, an ~8 nm SiN capping layer was deposited by RF sputtering at an Ar pressure of 10 mTorr immediately after Mn growth.*"

2) How would the results and interpretation of the Hanle magnetoresistance be affected if orbital (or even spin) Rashba effects were present at the Mn top surface or at the bottom interface with the MgO substrate? In particular, how do the authors exclude a possible orbital Rashba contribution, which could also give rise to magnetoresistance signals similar to those reported here (see, for example, Ref. [19])?

Response) We thank the reviewer for this insightful question, which allows us to further examine the possible role of interfacial effects in our experiments and to clarify the physical origin of the observed Hanle magnetoresistance (HMR). At the outset, we note that an orbital Rashba–Edelstein effect (OREE) [Salemi, L., *et al.*, *Nat. Commun.* **10**, 5381 (2019)] at the Mn surface or at the Mn/substrate interface

cannot be completely ruled out in principle. However, several experimental observations consistently indicate that such interfacial contributions are unlikely to be the dominant mechanism in our system.

First, we observe that the HMR signal is essentially independent of both the substrate and the capping layer. Specifically, comparable HMR responses are obtained for Mn films grown on MgO and on Si_3N_4 substrates, and for devices capped with either AlO_x or SiN. If the signal were primarily governed by an interfacial OREE (or even a spin REE) a strong dependence on the specific interface (substrate or capping material) would be expected. The absence of such dependence suggests that the dominant contribution does not originate from a particular interface.

Second, we exploit thickness-dependent measurements to test an interfacial scenario. For sputtered amorphous Mn films grown under different Ar pressures (**Fig. R10**), we observe a nonmonotonic dependence of the HMR amplitude on film thickness, with a pronounced peak at a thickness between 5 and 10 nm. Such behavior is incompatible with a simple interfacial Rashba mechanism, for which one would generally expect a monotonic decrease of the signal with increasing film thickness due to bulk shunting effects. Moreover, both the position of the peak and the overall thickness dependence shift systematically with the sputtering pressure, indicating a strong sensitivity to the film microstructure.

We further investigated epitaxial Mn films with thicknesses of 10 nm and 20 nm (**Fig. R11**). In both cases, clear and reproducible Hanle signatures are observed in the transverse magnetoresistance measured under out-of-plane magnetic fields, indicating robust orbital accumulation and precession in the films. Quantitative analysis using the same fitting procedure as in the main text yields orbital relaxation times of τ_L = 0.98 ps for the 10 nm film and τ_L = 1.32 ps for the 20 nm film, with all fitting parameters summarized in Table R2. Importantly, increasing the film thickness leads to a simultaneous increase in both the longitudinal conductivity and the orbital relaxation time. This trend follows the same Elliott–Yafet (EY)–like scaling between τ_L and conductivity discussed in Fig. 3(b) of the main text and is reproduced here within a single crystallographic phase by varying only the film thickness. The fact that τ_L increases systematically with conductivity, while the interfacial environment remains unchanged, strongly indicates that orbital relaxation is governed by bulk momentum-scattering processes rather than by interface-specific effects. If the observed relaxation were dominated by an interfacial Rashba mechanism, one would instead expect τ_L to be largely insensitive to film thickness once the thickness exceeds the interfacial region. The observed thickness-dependent enhancement of τ_L therefore provides additional evidence that the dominant relaxation mechanism in epitaxial Mn is bulk-controlled, consistent with the EY–like interpretation presented in the main text.

Taken together, the weak dependence on substrate and capping layers, the negligible expected spin contribution, and the nonmonotonic thickness dependence of the HMR all suggest that the bulk orbital Hall effect provides a more consistent interpretation of our results. While a minor interfacial contribution cannot be completely excluded, our data indicate that it does not play a dominant role in the observed magnetoresistance.

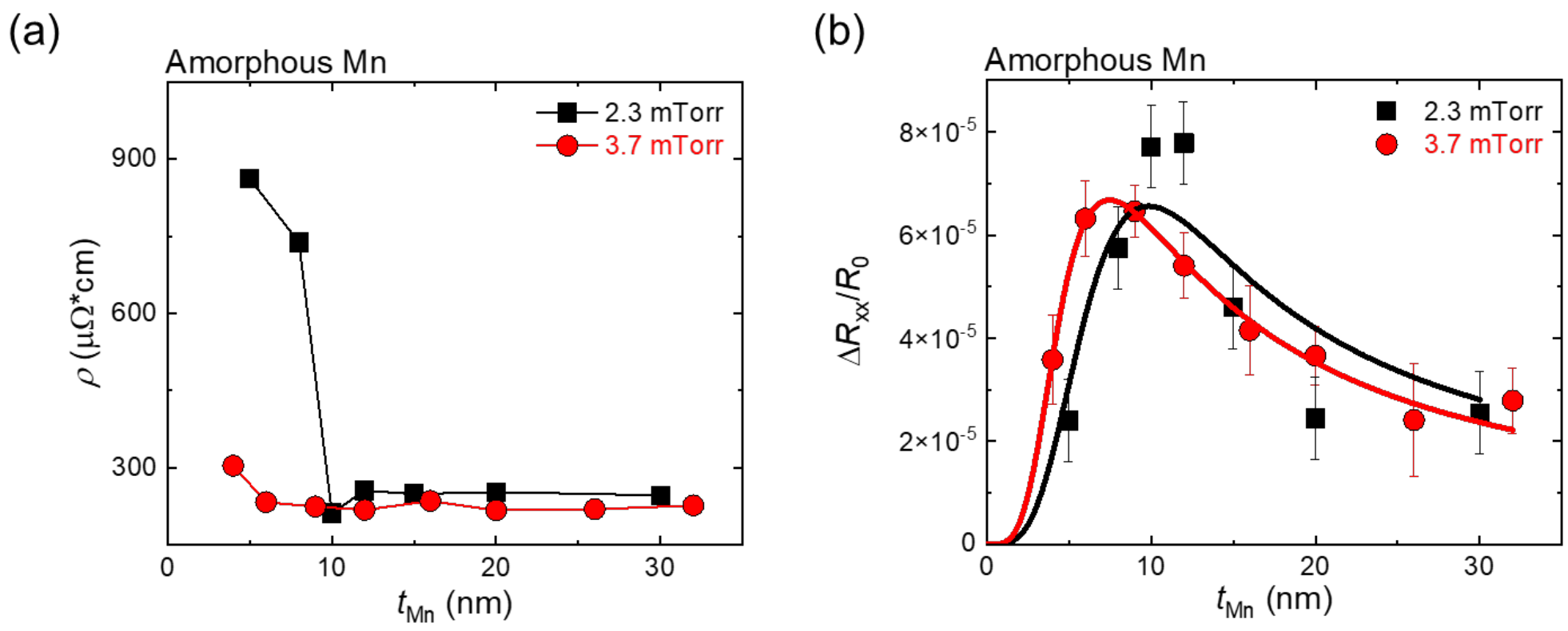

Figure R10 | Thickness-dependent resistivity and Hanle magnetoresistance ($\Delta R_{xx}/R_0$) in amorphous Mn films. **(a)** Longitudinal resistivity ρ as a function of Mn thickness t_{Mn} for films grown at 2.3 and 3.7 mTorr. **(b)** $\Delta R_{xx}/R_0$ as a function of t_{Mn} for the same samples.

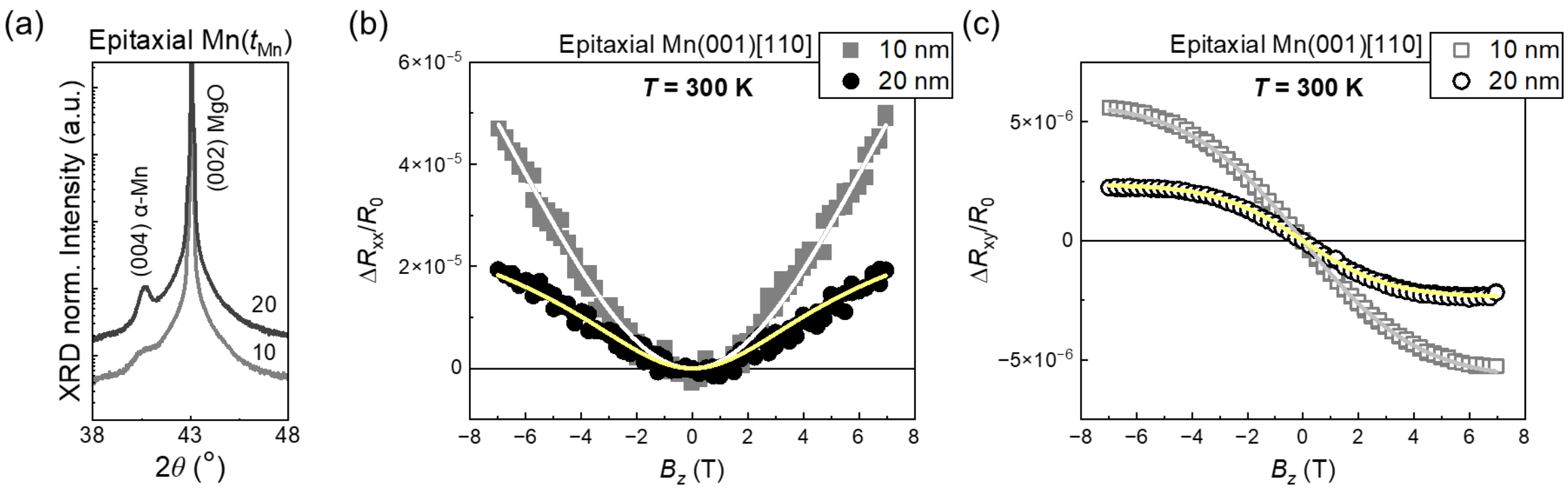

Figure R11 | Thickness-dependent Hanle magnetoresistance in epitaxial Mn. **(a)** Longitudinal Hanle magnetoresistance ($\Delta R_{xx}/R_0$) and **(b)** transverse Hanle magnetoresistance ($\Delta R_{xy}/R_0$) measured at 300 K for epitaxial Mn films with thicknesses of 10 nm and 20 nm under out-of-plane magnetic field B_z

Samples	σ $[10^3 \Omega^{-1} \text{cm}^{-1}]$	σ_L $[\hbar/e\ \Omega^{-1}\ \text{cm}^{-1}]$	τ [ps]	θ
Epi-Mn$_{LowT}$ (10 nm)	5.22	52 ± 10	0.98 ± 0.04	0.019 ± 0.001
Epi-Mn$_{LowT}$ (20 nm)	6.09	26 ± 12	1.32 ± 0.13	0.008 ± 0.005

Table R2 | Fitting parameters extracted from Hanle analysis. Extracted electrical conductivity (σ), orbital Hall conductivity (σ_L), orbital Hall angle (θ_{OH}), and orbital relaxation time (τ_L) extracted from Hanle fitting for epitaxial Mn films.

3) The manuscript does not describe the experimental protocol used to acquire the temperature dependence of the Hanle magnetoresistance. As emphasized by the authors, the temperature-induced changes in conductivity are relatively modest, requiring careful experimental procedures. Could the

authors specify how the temperature sweeps were performed (e.g., heating versus cooling), and whether any hysteresis or thermal stabilization effects were checked?

Response) We thank the reviewer for raising this relevant point regarding the experimental protocol used to acquire the temperature-dependent Hanle magnetoresistance data. We agree that, since the temperature-induced changes in conductivity are relatively modest, careful thermal control and stabilization are essential to ensure reliable measurements.

All temperature-dependent measurements were performed during heating from low temperature to room temperature. At each target temperature, the sample was first allowed to thermally stabilize for at least 10 minutes before any electrical measurement was started. After applying the measurement current, we waited an additional ~20 minutes to ensure full thermal equilibration of the device.

Magnetic-field sweeps were performed in both directions at each temperature. The initial and final points coincided within experimental resolution, and no thermal drift was detected.

These procedures ensure that the extracted temperature-dependent Hanle magnetoresistance and conductivity data are not affected by thermal lag or transient effects, and that the observed trends reflect intrinsic transport properties.

To clarify this point for the reader, we have now added the following description to the Methods section (page 10, lines 317-320): "*Temperature-dependent measurements were performed during heating. At each set temperature, the sample was stabilized for at least 10 min with no current applied and 20 min with current before starting the measurement to ensure thermal equilibrium. Magnetic-field sweeps were performed in both directions to verify the absence of thermal drift.*"

4) The orbital decoherence time in Mn extracted in this study is compared to typical spin-flip times in other 3d transition metals such as Ni or Cu, which are stated to be larger than 10 ps (Note that the two cited references [48-49] are not relevant to the sentence). While this assumption may be valid for Cu—where 3s–4d electronic scattering is absent—it is much less applicable to Fe, Co, or Ni, for which spin-flip times are generally expected to lie in the picosecond range or below.

Indeed, recent THz time-domain spectroscopy (THz-TDS) measurements report typical spin-flip times of approximately 300 fs for Fe and Ni (G. Bierhance et al., arXiv:2506.22327), and below 500 fs for CoFeB (R. Rouzegar et al., Phys. Rev. B 106, 144427 (2022)). These values are reported to be largely insensitive to surface quality or interfacial conditions, irrespective of the adjacent metallic layer.

May the author can comment on this issue?

Response) We thank the reviewer for this insightful comment regarding the comparison between the orbital relaxation time extracted in our work and previously reported spin-flip times in 3d transition

metals. We agree that our original statement was too general and that the cited reference [48-49] was not appropriate to support this comparison, particularly for ferromagnetic metals such as Fe and Ni. We have therefore revised this part of the manuscript.

First, we fully acknowledge the reviewer's point that recent THz time-domain spectroscopy (THz-TDS) measurements report ultrafast spin-flip times on the order of a few hundred femtoseconds in ferromagnetic 3d metals such as Fe and Ni [G. Bierhance et al., arXiv:2506.22327; R. Rouzegar et al., *Phys. Rev. B* **106**, 144427 (2022)]. These studies demonstrate that microscopic spin-flip processes in these materials can occur on sub-picosecond timescales. We therefore agree that our original wording implying long spin relaxation times in Ni was inaccurate and have corrected this in the revised manuscript.

However, the relaxation times obtained from THz-TDS and from magnetotransport correspond to different physical quantities and regimes. THz-TDS probes ultrafast nonequilibrium spin dynamics on femtosecond timescales excited by fs-laser-induced transient nonequilibrium electron distributions, whereas relaxation times derived from diffusive spin transport experiments represent effective decoherence times governing angular-momentum diffusion after multiple scattering events. For this reason, the two timescales are not directly comparable on a one-to-one basis.

To place the comparison on a consistent experimental footing, we therefore compare our orbital relaxation time in Mn with spin relaxation times obtained from transport measurements in transition-metal films. Spin diffusion experiments in ferromagnetic metals such as Fe and Ni typically yield spin relaxation times in the several to tens of picosecond range in the diffusive regime [J. Bass and W. P. Pratt Jr., *J. Phys.: Condens. Matter* **19**, 183201 (2007) and references therein].

This comparison class is also physically appropriate because Mn, even in the paramagnetic phase, carries local magnetic moments that act as intrinsic spin-scattering centers. Mn therefore cannot be directly equated with truly nonmagnetic weak-SOC metals such as Cu, Ag, or Al. Instead, comparison with magnetic transition metals provides a more meaningful reference. Within this framework, our observation of sub-picosecond to few-picosecond orbital relaxation times in Mn indicates that orbital angular momentum decoheres on a timescale comparable to or shorter than spin relaxation in magnetic 3d metals. This reinforces the conclusion that orbital relaxation in Mn is intrinsically very rapid.

Finally, the shortest orbital relaxation time observed in crystalline Mn films is consistent with recent first-principles scattering calculations predicting that orbital transport is strongly limited by the crystal-field potential, leading to orbital diffusion lengths of only a few unit cells [K. D. Belashchenko *et al.*, *Phys. Rev. B* **108**, 144433 (2023); M. Rang and P. Kelly, *Phys. Rev. B* **109**, 214427 (2024); M. Rang and P. Kelly, *Phys. Rev. B* **111**, 125121 (2025)]. Such limited diffusion naturally implies much shorter orbital transport and relaxation timescale compared to spin.

Based on these considerations, we have revised the manuscript accordingly. Specifically, we replaced Ref. [49] with the transport-based review of spin relaxation in metals [J. Bass and W. P. Pratt Jr., *J. Phys.: Condens. Matter* **19**, 183201 (2007)]. On page 6, lines 199-201, we now state that: "*These values are significantly smaller than spin relaxation times reported for transition-metal thin films in the diffusive transport regime [49], indicating that orbital relaxation in Mn is intrinsically faster than spin relaxation.*"

This revision removes the inappropriate comparison with THz spin-flip times and places the orbital–spin comparison on a consistent transport-based footing appropriate for magnetic metals.

Summary of Changes:

We have grown new samples and performed additional experiments to answer critical questions posed by the reviewers. Additionally, we have revised the manuscript and Supplementary Information to clarify the points raised by the reviewers. The main changes are summarized below.

(i) Additional structural characterization by transmission electron microscopy: Cross-sectional HAADF-STEM analysis of epitaxial, polycrystalline, and amorphous Mn films has been added (Fig. 1c; Supplementary Fig. S4). The TEM results confirm epitaxial α-Mn ordering, the granular multi-phase structure of polycrystalline Mn, and pressure-dependent nanoscale disorder in amorphous Mn.

(ii) Additional magnetic and transport measurements: New SQUID magnetometry measurements were performed on polycrystalline Mn films to independently examine the presence of antiferromagnetic ordering. These data, now included in Supplementary Note 1 (Supplementary Fig. S7), confirm the absence of a clear magnetic transition signal within the experimental resolution.

(iii) Expanded dataset for orbital relaxation scaling: Additional epitaxial Mn samples with varied thickness, growth temperature, and substrate preparation were grown, measured, and incorporated into Fig. 3. The EY-like scaling of τ_L with conductivity in metallic Mn is confirmed using the expanded dataset.

(iv) Clarification of disorder definition in amorphous Mn: The definition of disorder in amorphous Mn has been revised in light of the TEM analysis and the transport-relevant disorder correlation length, rather than to surface roughness alone. Additional thickness- and temperature-dependent transport measurements supporting this definition are included in Supplementary Fig. S5.

(v) Clarification of transport regimes: The scaling $\sigma_L \propto \sigma$ has been explicitly referred to the hopping-dominated low-conductivity regime, and the discussion now distinguishes more clearly between hopping and metallic transport regimes in Figs. 3-4 and in the Discussion.

(vi) Clarification of physical interpretation: The orbital Hall response in amorphous Mn is clarified as a hopping-mediated transverse transport phenomenon rather than skew or side-jump scattering; the comparison between orbital and spin relaxation times has been revised using transport-based references appropriate for transition-metal films.

(vii) Methods additions: Surface capping procedures for MBE-grown and sputtered Mn films, temperature-dependent measurement protocols, and cross-sectional HAADF-STEM procedures have been added.

(viii) Additional discussion of interfacial effects: The possible role of interfacial contributions, including orbital Rashba-Edelstein effect, has been examined and discussed. These results, now included

in Supplementary Note 4 (Supplementary Fig. S12), together with thickness-dependent HMR measurements and the weak dependence on substrate and capping layers indicate that the observed signals are dominated by bulk orbital transport.

All changes in the manuscript and Supplementary Information are highlighted in blue.

Reviewer comments:

Reviewer #2 (Remarks to the Author):

The authors have addressed most of the concerns raised in the initial review and clarified the scope of their claims. In particular, they now demonstrate more clearly that the OHC–conductivity scaling emerges in the amorphous, hopping-dominated regime. While the experimental analysis is careful and the dataset is valuable, and although further theoretical development would still be desirable, the central claim is now more precisely formulated.

However, it remains unclear whether the level of conceptual novelty meets the standards expected for Nature Physics. Closely related scaling behavior has already been established for the spin Hall effect across metallic and disordered regimes [Commun. Phys. 5, 12 (2022)]. In this context, the present results appear largely as an orbital counterpart of an already known phenomenology, without yet demonstrating qualitatively new physics beyond the spin case. The observation of a non-monotonic orbital relaxation time is interesting, but it is not yet clear whether this reflects a genuinely orbital-specific effect. As a side point, given that orbital transport is known to be sensitive to parameters such as film thickness, temperature, and interfacial conditions, it would be important to establish whether the proposed scaling remains robust across different amorphous samples. For example, it would be useful to clarify whether a similar scaling behavior persists for amorphous Mn films with different thicknesses.:

Response) We thank the reviewer for the careful evaluation of our manuscript and for recognizing the improved clarity of the revised version, as well as the quality and scope of the experimental dataset.

We agree that the scaling behavior reported in our work bears a formal similarity to that established for the spin Hall effect. However, the central point of our study is the orbital Hall effect. In particular, our measurements extend orbital Hall transport into the strongly disordered, hopping-dominated regime, where charge transport is governed by thermally activated processes rather than coherent band conduction. While similar scaling behavior has been discussed for anomalous and spin Hall effects in disordered systems, its extension to orbital Hall transport has not been demonstrated before, neither experimentally, nor theoretically.

Moreover, we identify orbital relaxation mechanisms that have not been discussed in the context of spin Hall physics in metallic conductors, such as on-site orbital relaxation and interorbital hopping, before the onset of Elliott-Yafet-type relaxation in crystalline samples. Current experimental results on orbital transport and relaxation are rather controversial, ranging from claims of strong crystal-field induced dephasing to the opposite limit of long-range orbital transport with orbital diffusion lengths exceeding spin diffusion lengths. Our data provide direct access to the orbital relaxation time within a single material system, allowing us to correlate orbital generation and relaxation across different structural and transport regimes. In general, we find that orbital relaxation is strongly temperature-dependent in the hopping regime and accelerated by crystal field dephasing in ordered samples.

Regarding the robustness of the observed scaling, we note that the dataset already includes multiple amorphous and polycrystalline samples spanning different thicknesses, growth conditions, and degrees of structural disorder, as well as additional measurements in the metallic regime (Supplementary Figs. S9–S11). Across these samples, the scaling behavior is consistently observed within the hopping-dominated regime. These results indicate that the scaling is not specific to a single sample or set of conditions, but is reproducible across different realizations of disordered Mn films within the same transport regime.

We hope that this clarification better conveys the scope and significance of our results.

Report on '*Orbital Hall conductivity in Mn thin films with variable disorder'* by Min-Gu Kang et al.

This manuscript presents a detailed and fundamental investigation of the orbital Hall conductance (OHC) in Mn compounds exhibiting different degrees of disorder and transport regimes, ranging from epitaxial metallic and textured films to polycrystalline and amorphous samples, all grown under various sputtering conditions. In this study, Mn is chosen as an ideal prototypical material owing to its very small spin Hall conductivity and its low equilibrium orbital angular momentum (singlet state).

Using orbital Hanle magnetoresistance measurements performed over a wide temperature range, the authors rigorously demonstrate: (i) the existence and quantitative determination of the OHC in Mn, leading to orbital current generation and orbital polarization at the sample edges; (ii) the dependence of the OHC on disorder; and (iii) the dependence of the orbital relaxation (or decoherence) time on both disorder and phonon scattering rates (temperature).

The main results of this work include the demonstration of an intrinsic OHC (i.e., conductivity-independent) in the epitaxial and textured Mn series, and a conductivity-dependent OHC in amorphous samples, where variable-range hopping transport dominates. In contrast, the orbital decoherence time exhibits a more complex behavior: it increases with hopping conductivity as disorder is varied, while it decreases when phonon scattering is enhanced at higher temperatures. This behavior points to a crossover in the dominant orbital decoherence mechanisms depending on the underlying scattering process.

In my view, the breadth of systems investigated and the depth of the experimental analysis are highly impressive, and the conclusions drawn are both robust and of significant value to the rapidly emerging field of orbitronics. As this field continues to grow, the results presented here will undoubtedly prove to be important and broadly useful to the scientific community.

I therefore conclude that this work merits rapid publication in *Nature Physics*, provided that the authors adequately address the comments and questions raised below.

1) One of my my main concern relates to the quality of the Mn top surface investigated in the Hanle transport measurements. The manuscript does not address the possibility of slight oxidation or surface contamination of the Mn Hall bar, which could significantly alter—or even compromise—the measured magnetoresistance properties. This issue is neither discussed in the main text nor in the Supplementary Information.
   Could the authors please comment on this important point and clarify how surface oxidation or contamination is controlled or ruled out?

2) How would the results and interpretation of the Hanle magnetoresistance be affected if orbital (or even spin) Rashba effects were present at the Mn top surface or at the bottom interface with the MgO substrate?
   In particular, how do the authors exclude a possible orbital Rashba contribution, which could also give rise to magnetoresistance signals similar to those reported here (see, for example, Ref. [19])?

3) The manuscript does not describe the experimental protocol used to acquire the temperature dependence of the Hanle magnetoresistance. As emphasized by the authors, the temperature-induced changes in conductivity are relatively modest, requiring careful experimental

procedures.

Could the authors specify how the temperature sweeps were performed (e.g., heating versus cooling), and whether any hysteresis or thermal stabilization effects were checked?

4) The orbital decoherence time in Mn extracted in this study is compared to typical spin-flip times in other 3d transition metals such as Ni or Cu, which are stated to be larger than 10 ps (*Note that the two cited references [48-49] are not relevant to the sentence*). While this assumption may be valid for Cu—where 3s–4d electronic scattering is absent—it is much less applicable to Fe, Co, or Ni, for which spin-flip times are generally expected to lie in the picosecond range or below.

   Indeed, recent THz time-domain spectroscopy (THz-TDS) measurements report typical spin-flip times of approximately 300 fs for Fe and Ni (G. Bierhance *et al.*, arXiv:2506.22327), and below 500 fs for CoFeB (R. Rouzegar *et al.*, *Phys. Rev. B* **106**, 144427 (2022)). These values are reported to be largely insensitive to surface quality or interfacial conditions, irrespective of the adjacent metallic layer.

   May the author can comment on this issue ?
